# Supplementary material for: Identification of a phosphorylation site on Ulk1 required for genotoxic stress-induced alternative autophagy
Source: Nat Commun. 2020 Apr 9;11:1754. doi: 10.1038/s41467-020-15577-2 (PMC7145817; doi:10.1038/s41467-020-15577-2)
Supplement: Supplementary file 1 — Supplementary Information [file 41467_2020_15577_MOESM1_ESM.pdf]

Identification of a phosphorylation site on Ulk1 required for genotoxic stress-induced alternative autophagy

Torii et al.

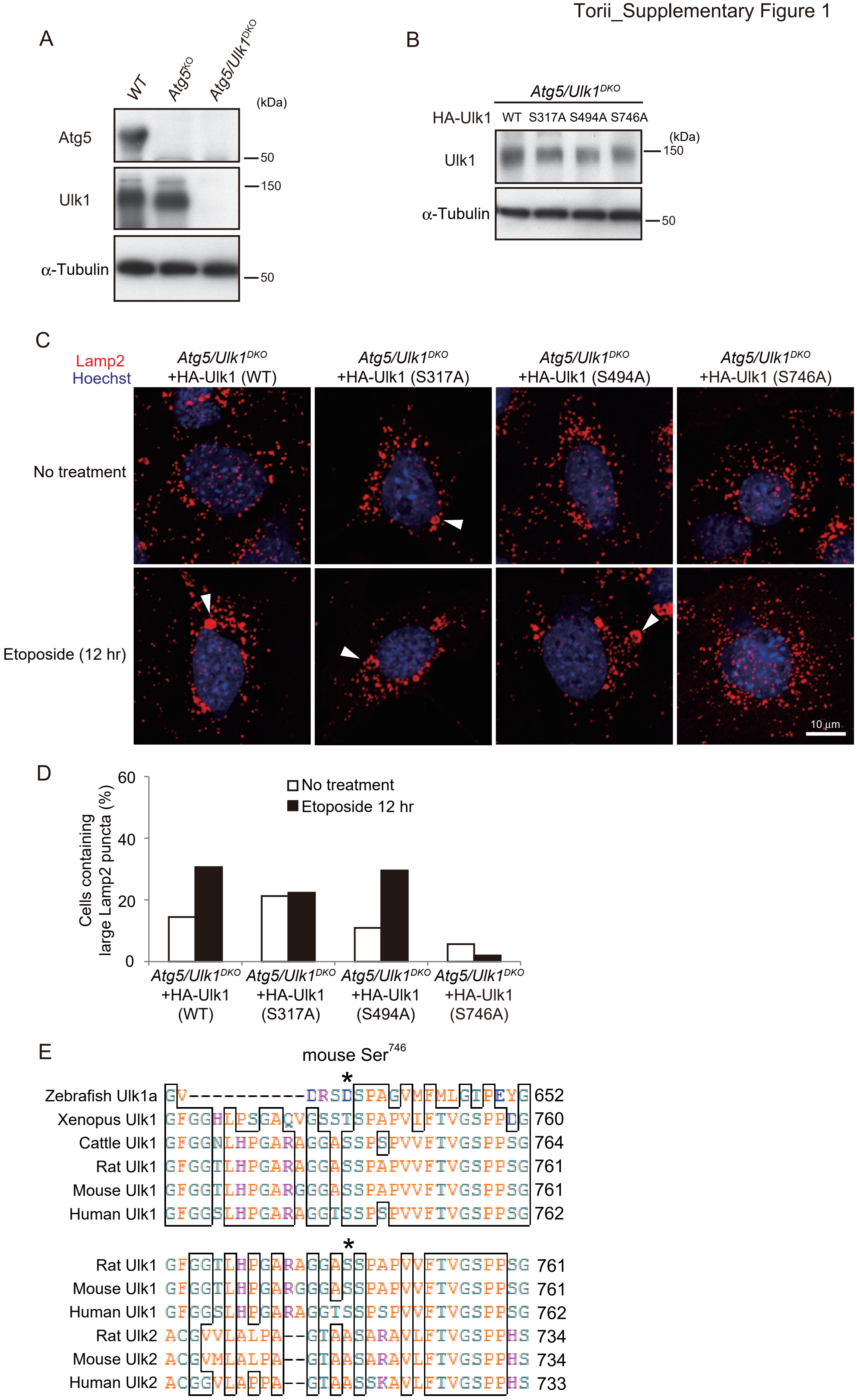
A
WT
Atg5KO
Atg5/Ulk1DKO
(kDa)
Atg5
Ulk1
α-Tubulin
50
150
50
B
Atg5/Ulk1DKO
HA-Ulk1 WT S317A S494A S746A
Ulk1
α-Tubulin
(kDa)
150
50
C
Lamp2
Hoechst
Atg5/Ulk1DKO +HA-Ulk1 (WT)
Atg5/Ulk1DKO +HA-Ulk1 (S317A)
Atg5/Ulk1DKO +HA-Ulk1 (S494A)
Atg5/Ulk1DKO +HA-Ulk1 (S746A)
No treatment
Etoposide (12 hr)
10 µm
D
Cells containing large Lamp2 puncta (%)
0
20
40
60
No treatment
Etoposide 12 hr
Atg5/Ulk1DKO +HA-Ulk1 (WT)
Atg5/Ulk1DKO +HA-Ulk1 (S317A)
Atg5/Ulk1DKO +HA-Ulk1 (S494A)
Atg5/Ulk1DKO +HA-Ulk1 (S746A)
E
mouse Ser746
*
Zebrafish Ulk1a GV-----------DRSDDSPAGVMFMLGIPEYG 652
Xenopus Ulk1 GFGGHLPSGAQVGSSTSPAPVIFTVGSPPDG 760
Cattle Ulk1 GFGGNLHPGARAGGASSPSPVVFTVGSPPSG 764
Rat Ulk1 GFGGTLHPGARAGGASSPAPVVFTVGSPPSG 761
Mouse Ulk1 GFGGTLHPGARGGGASSPAPVVFTVGSPPSG 761
Human Ulk1 GFGGSLHPGARAGGTSSPSPVVFTVGSPPSG 762
*
Rat Ulk1 GFGGTLHPGARAGGASSPAPVVFTVGSPPSG 761
Mouse Ulk1 GFGGTLHPGARGGGASSPAPVVFTVGSPPSG 761
Human Ulk1 GFGGSLHPGARAGGTSSPSPVVFTVGSPPSG 762
Rat Ulk2 ACGVVLALPA--GTAASARAVLFTVGSPPHS 734
Mouse Ulk2 ACGVMLALPA--GTAASARAVLFTVGSPPHS 734
Human Ulk2 ACGGVLAPPA--GTAASSKAVLFTVGSPPHS 733

Supplementary Figure 1. Generation of *Atg5/Ulk1^{DKO}* MEFs and crucial role of Ulk1 phosphorylation at Ser^{746} in alternative autophagy. **(A)** Confirmation using western blotting of the successful generation of *Atg5/Ulk1^{DKO}* MEFs by the crossbreeding of *Atg5^{heteroKO}* mice and *Ulk1^{KO}* mice. **(B)** The expression of Ulk1 mutants in *Atg5/Ulk1^{DKO}* MEFs was confirmed by western blot analysis. **(C, D)** The indicated MEFs were treated with or without etoposide (10 μM) for 12 hr, and immunostained with an anti-Lamp2 antibody. Nuclei were counterstained with Hoechst 33342. Representative images are shown in **(C)**. In **(C)**, arrowheads indicate autolysosomes. In **(D)**, the population of cells with large Lamp2 puncta was calculated ($n \geq 100$ cells). **(E)** Alignment of amino acid sequences surrounding Ser^{746} of mouse Ulk1 with vertebrate Ulk1 and Ulk2.

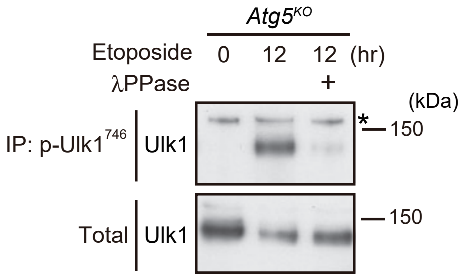

Supplementary Figure 2. Lambda protein phosphatase-dependent decrease in p-Ulk1^{746} signals. *Atg5^{KO}* MEFs were treated with etoposide (10 μM) for 12 hr. Cells were then lysed and treated with λPPase and immunoprecipitated with an anti-p-Ulk1^{746} antibody. Immune complexes and total lysates (2.8% input) were analyzed by western blotting using an anti-Ulk1 antibody. Asterisk indicates non-specific band.

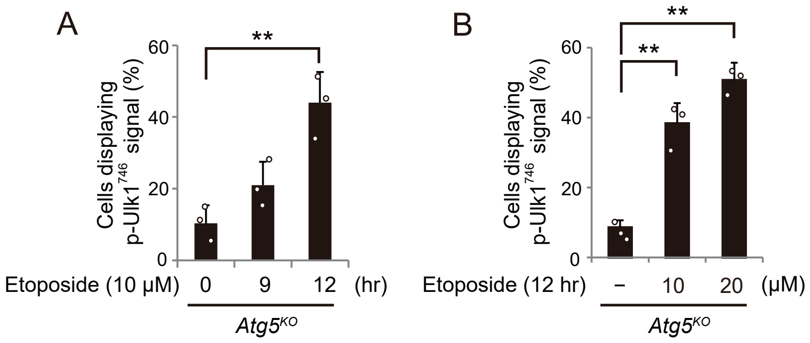

Supplementary Figure 3. Time-dependent and dose-dependent increase in p-Ulk1^{746} signals upon etoposide treatment. Quantification of cells displaying p-Ulk1^{746} signals. *Atg5^{KO}* MEFs were treated with the indicated doses of etoposide for the indicated hours, and immunostained with an anti-p-Ulk1^{746} antibody. The population of cells with p-Ulk1^{746} signals was calculated ($n \geq 100$ cells in each experiment). Data are shown as the mean ± SD ($n = 3$). In (A), *Atg5^{KO}* no treatment vs. Etoposide 12 hr: p=0.0023. In (B), *Atg5^{KO}* no treatment vs. Etoposide 10 µM: p=0.0005. Other exact p values cannot be described since the value is too large (p>0.9999) or small (p<0.0001). Comparisons were performed using one-way ANOVA followed by the Tukey post-hoc test. **$p < 0.01$. Source data are provided as a Source Data file.

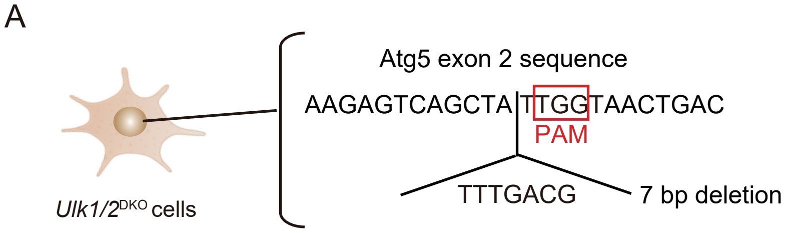

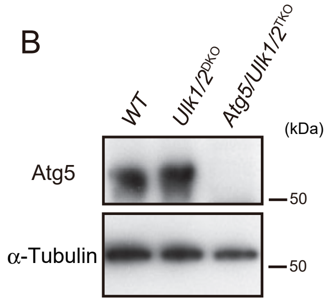

Supplementary Figure 4. Generation of *Atg5/Ulk1/2*^{TKO} MEFs. (A) Schematic design of the Atg5 guide RNA. Using this sequence, we generated *Atg5/Ulk1/2*^{TKO} MEFs from *Ulk1/2*^{DKO} MEFs. **(B)** The deletion of Atg5 was confirmed by western blot analysis.

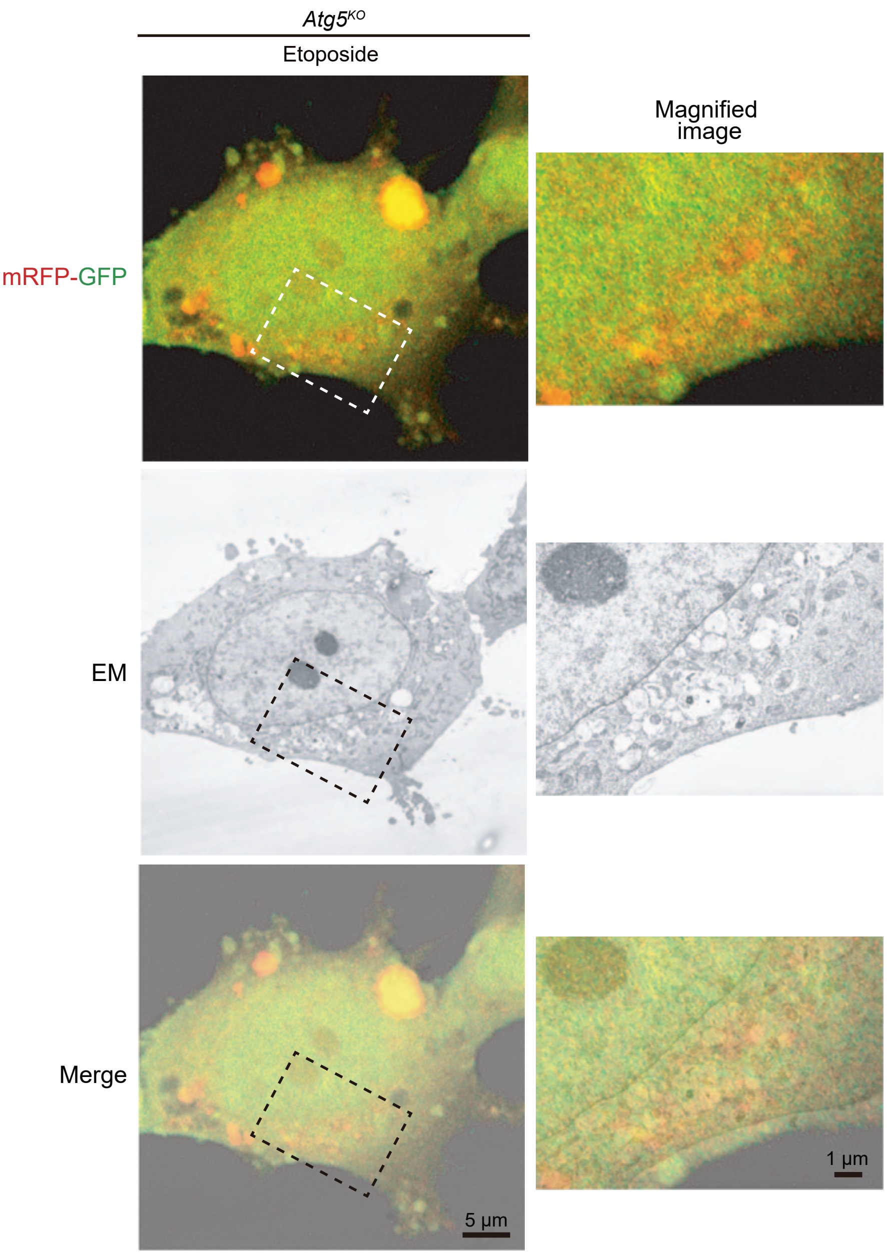

Supplementary Figure 5. CLEM analysis of mRFP-GFP-expressing *Atg5^KO^* MEFs upon etoposide treatment. Cells were treated with etoposide (10 μM) for 12 hr and observed using fluorescence and electron microscopy. Red puncta were merged with the autophagic vacuoles. Magnified images are shown at the right.

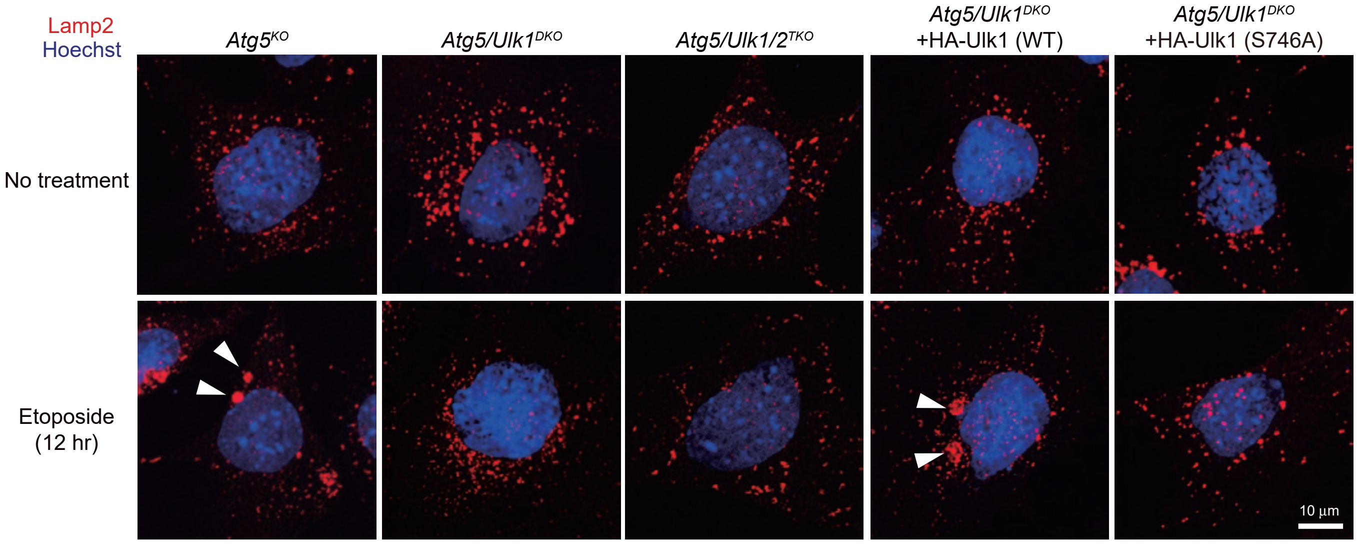

Supplementary Figure 6. Crucial role of Ulk1 phosphorylation at Ser^{746} in alternative autophagy. The indicated MEFs were treated with or without etoposide (10 µM) for 12 hr, and immunostained with an anti-Lamp2 antibody. Nuclei were counterstained with Hoechst 33342. Representative images are shown. Arrowheads indicate large Lamp2 puncta.

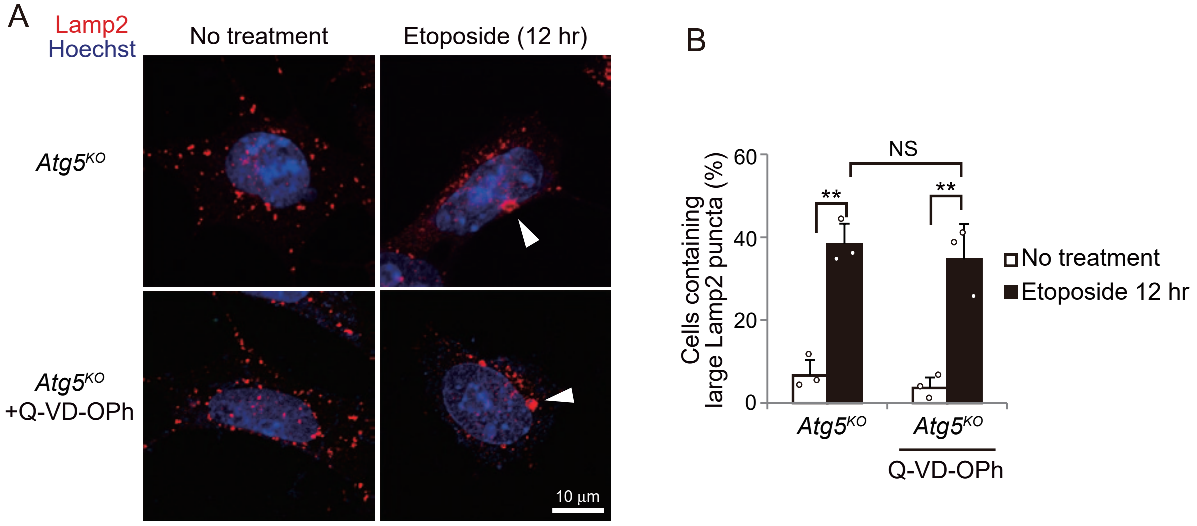

Supplementary Figure 7. Apoptosis is not involved in etoposide-induced alternative autophagy. *Atg5*^{KO} MEFs were treated with or without etoposide (10 µM) and Q-VD-OPh (50 µM) for 12 hr, and immunostained with an anti-Lamp2 antibody. Nuclei were counterstained with Hoechst 33342. Representative images are shown in **(A)**. In **(B)**, the population of cells with large Lamp2 puncta was calculated ($n \geq 100$ cells). Data are shown as the mean ± SD (n = 3). *Atg5*^{KO} no treatment vs. Etoposide: p=0.0004, *Atg5*^{KO} (Q-VD-OPh) no treatment vs. Etoposide: p=0.0005, *Atg5*^{KO} vs. *Atg5*^{KO} (Q-VD-OPh), Etoposide: p=0.8394. Comparisons were performed using one-way ANOVA followed by the Tukey post-hoc test. **$p < 0.01$; NS: not significant.

Torii_Supplementary Figure 8

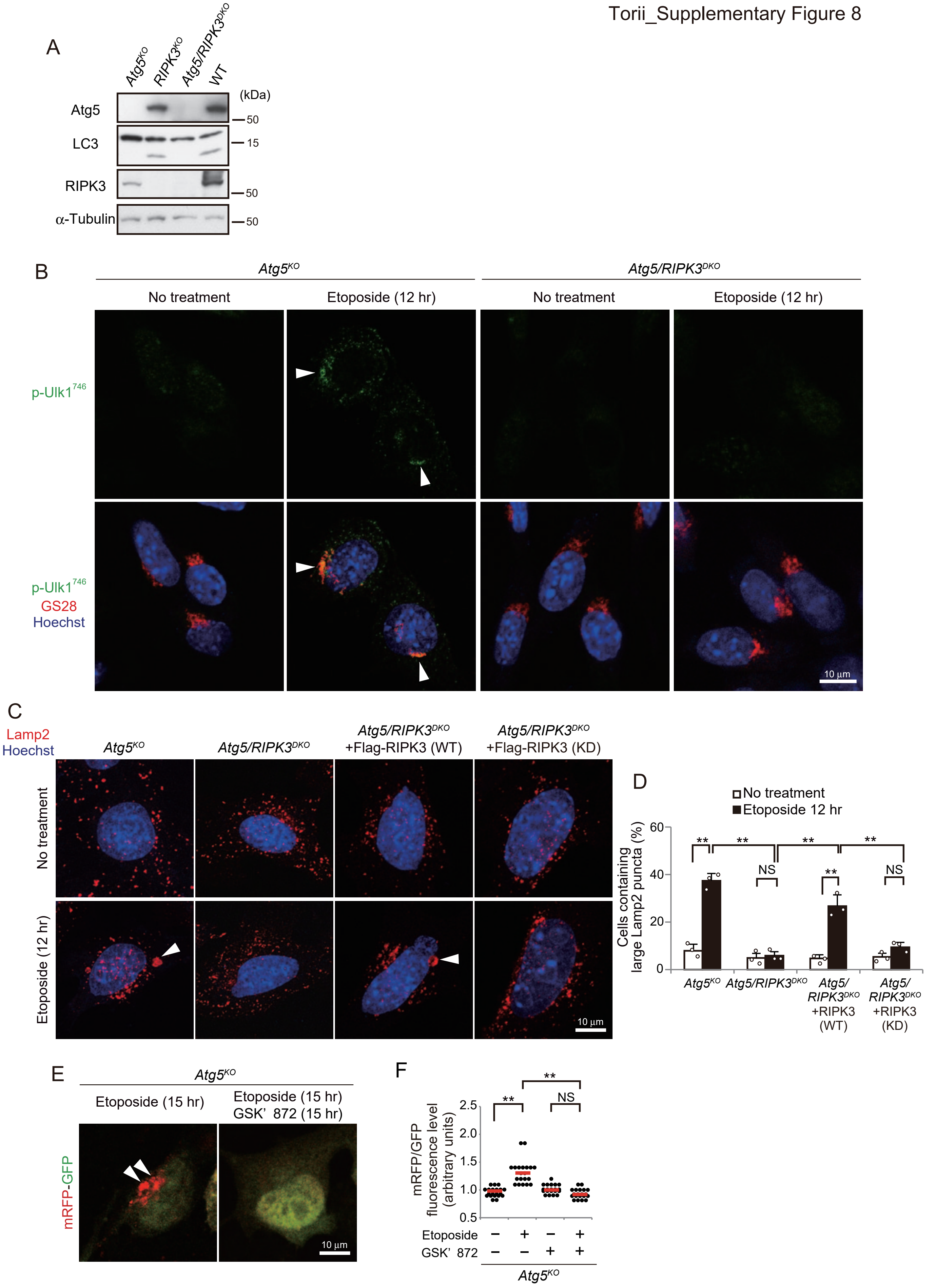

Supplementary Figure 8. Involvement of RIPK3 in etoposide-induced alternative autophagy. (A) The indicated untreated MEFs were subjected to western blotting to confirm the absence of each protein. **(B)** Multi-cell image showing the requirement of RIPK3 for etoposide-induced phosphorylation of Ulk1 at Ser^{746}. The indicated MEFs were treated with or without 10 µM of etoposide for 12 hr, and immunostained with anti-p-$Ulk1^{746}$ (green) and anti-GS28 (red) antibodies. Nuclei were counterstained with Hoechst 33342. Representative images of p-$Ulk1^{746}$ (upper panels) and merged images (lower panels) are shown. Arrowheads indicate p-$Ulk1^{746}$ signals. **(C, D)** The indicated MEFs were treated with or without etoposide (10 µM) for 12 hr, and immunostained with an anti-Lamp2 antibody. Nuclei were counterstained with Hoechst 33342. Representative images are shown. Arrowheads indicate large Lamp2 puncta. In **(D)**, the population of cells with large Lamp2 puncta was calculated ($n \geq 100$ cells). Data are shown as the mean ± SD (n = 3 experiments). *Atg5/RIPK3*^{DKO} no treatment vs. Etoposide: p=0.9997, *Atg5/RIPK3*^{DKO}+RIPK3 (KD) no treatment vs. Etoposide: p=0.5919. **(E, F)** The *Atg5*^{KO} MEFs were transiently transfected with a mRFP–GFP plasmid. After 24 hr, cells were treated with etoposide (10 µM) for 15 hr with or without GSK'872 (10 µM). Nuclei were counterstained with Hoechst 33342. Representative images are shown. Arrowheads indicate mRFP–GFP red puncta (acidic compartment). In **(F)**, the extent of red puncta was indicated by the RFP/GFP ratio per cell (n = 20 cells). Red lines indicate the mean value. Data are shown as the mean ± SD (n = 3 experiments). *Atg5*^{KO} GSK'872 vs. Etoposide/GSK'872: p=0.1246. Comparisons were performed using one-way ANOVA followed by the Tukey post-hoc test. **p < 0.01; NS: not significant.

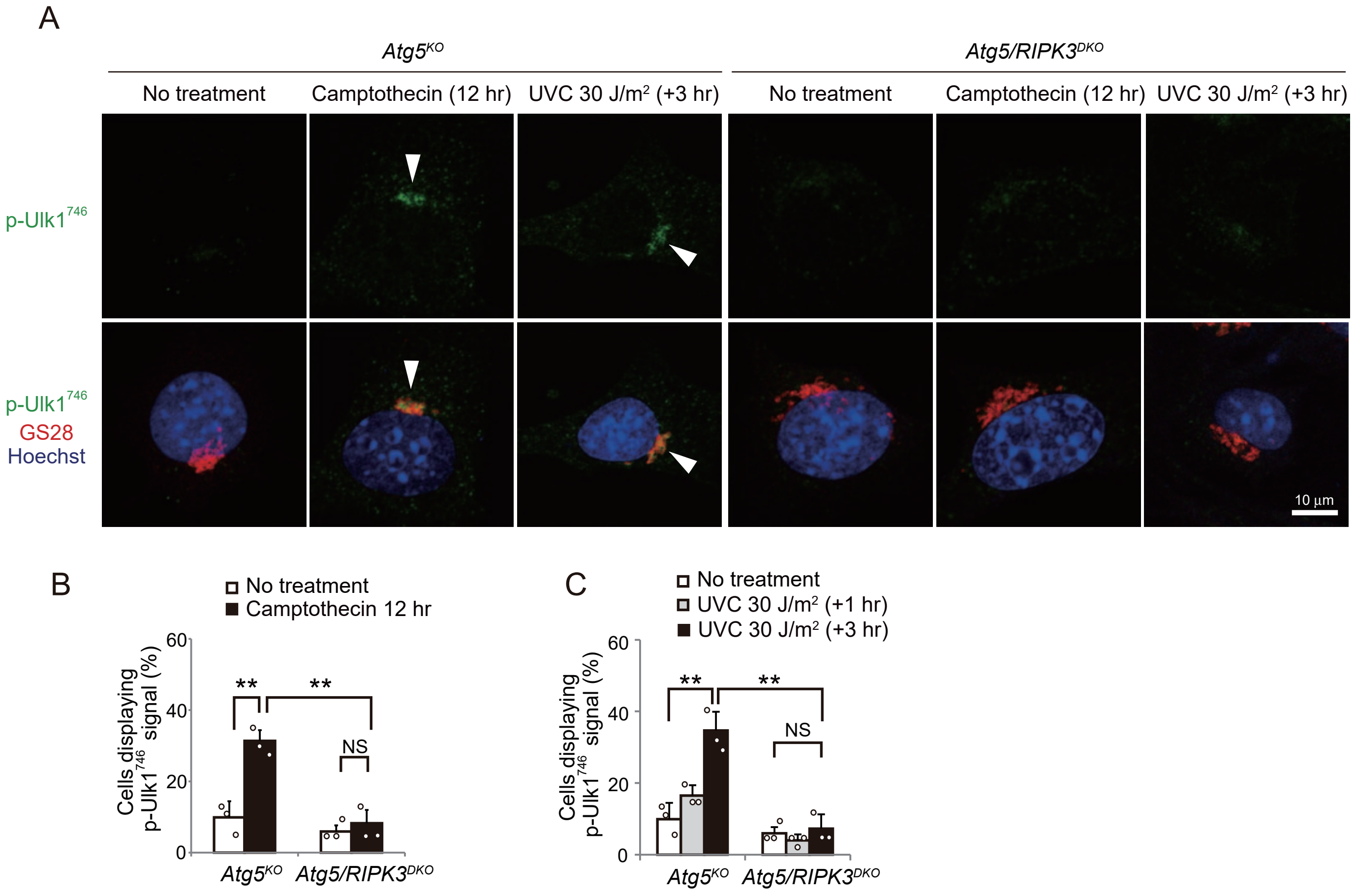

Supplementary Figure 9. Induction of the localization of p-Ulk1^{746} on the Golgi by camptothecin and UVC treatment. The indicated MEFs were treated with or without 20 µM of camptothecin for 12 hr or 30 J m^{2} $^{-1}$ UVC exposure for 3 hr, and immunostained with anti-p-Ulk1^{746} (green) and anti-GS28 (red) antibodies. Nuclei were counterstained with Hoechst 33342. **(A)** Representative images of p-Ulk1^{746} (upper panels) and merged images (lower panels) are shown. Arrowheads indicate p-Ulk1^{746} signals. **(B, C)** Quantification of cells displaying p-Ulk1^{746} signals upon camptothecin **(B)** and UVC treatment **(C)**. The population of cells with p-Ulk1^{746} signals was calculated ($n \geq 100$ cells in each experiment). Data are shown as the mean ± SD ($n = 3$). In (B), *Atg5^{KO}* no treatment vs. Camptothecin: p=0.0003, *Atg5/RIPK3^{DKO}* no treatment vs. Camptothecin: p=0.8409, *Atg5^{KO}* vs. *Atg5/RIPK3^{DKO}* Camptothecin: p=0.0002. In (C), *Atg5/RIPK3^{DKO}* no treatment vs. UVC (+3 hr): p=0.9969. Comparisons were performed using one-way ANOVA followed by the Tukey post-hoc test. **$p < 0.01$; NS: not significant.

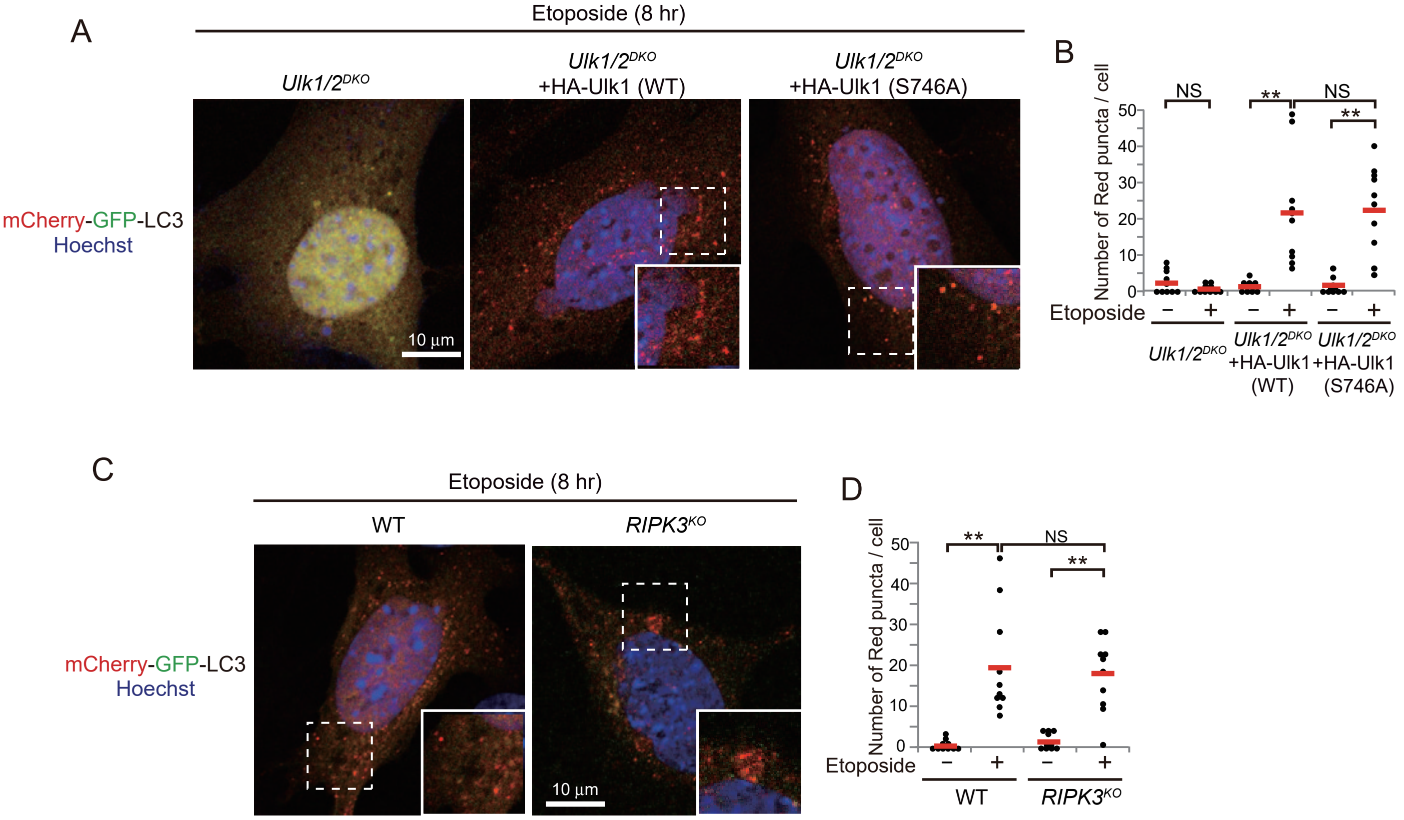

Supplementary Figure 10. No involvement of Ulk1 Ser^{746} phosphorylation and RIPK3 in etoposide-induced canonical autophagy. The indicated MEFs were transiently transfected with the mCherry-GFP-LC3 plasmid. After 24 hr, cells were treated with or without etoposide (10 μM) for 8 hr. Nuclei were counterstained with Hoechst 33342. **(A, C)** Representative images are shown. Magnified images of the areas within the dashed squares are shown in the insets. Yellow and red puncta indicate isolation membranes/autophagosomes and autolysosomes, respectively. **(B, D)** The number of red puncta (autolysosome) per cell was calculated ($n = 10$ cells in each experiment). Red lines indicate mean values. In (B), *Ulk1/2^{DKO}* no treatment vs. Etoposide: p=0.9971. In (D), *RIPK3^{KO}* no treatment vs. Etoposide: p=0.0003, WT vs. *RIPK3^{KO}*, Etoposide: p=0.9418. Comparisons were performed using one-way ANOVA followed by the Tukey post-hoc test. **$p < 0.01$. NS: not significant.

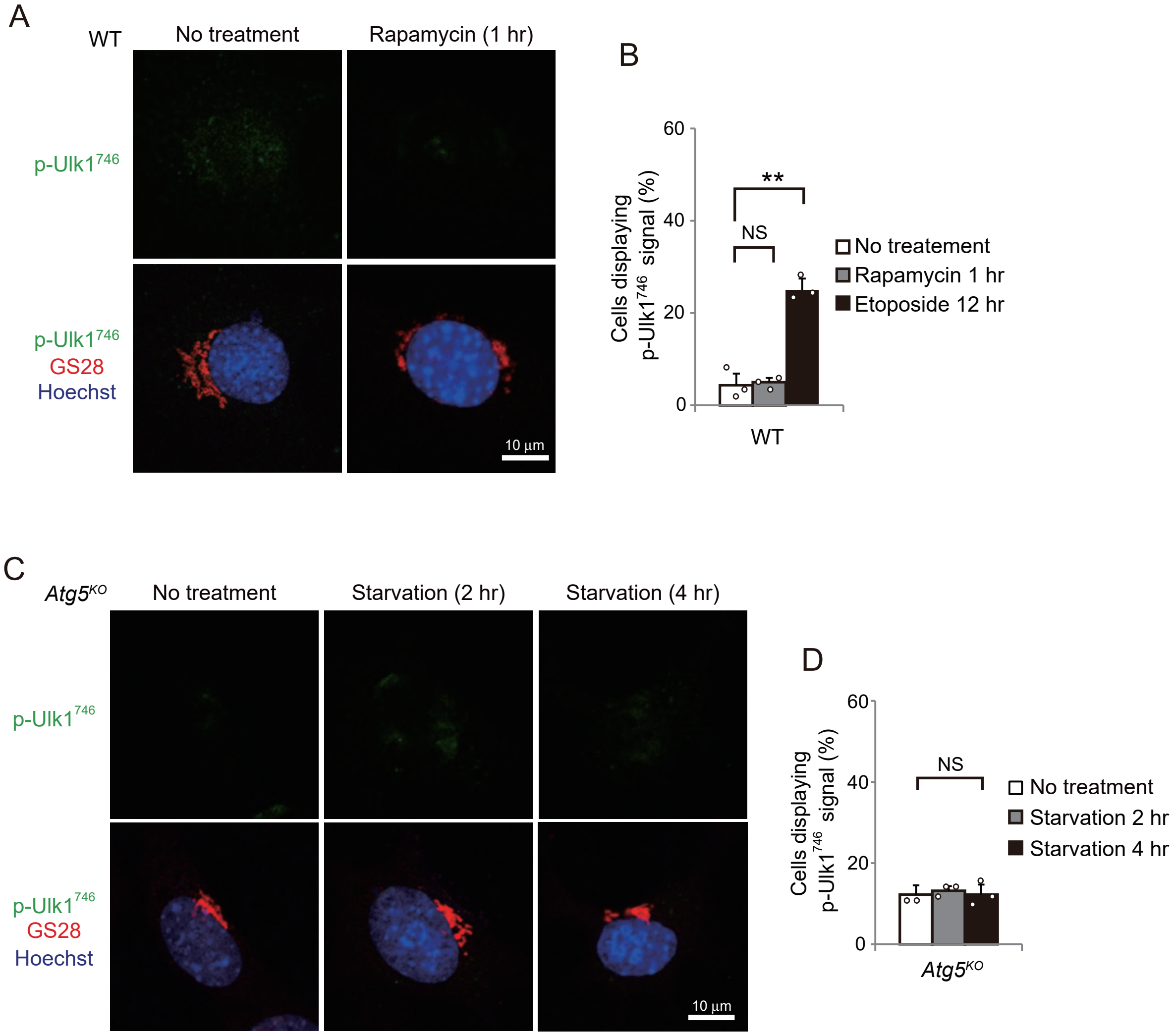

Supplementary Figure 11. Rapamycin and starvation do not induce the phosphorylation of Ulk1 at Ser^{746}. **(A)** WT MEFs were treated with or without rapamycin (0.5 µM) for 1 hr, and immunostained with anti-p-Ulk1^{746} (green) and anti-GS28 (red) antibodies. Nuclei were counterstained with Hoechst 33342 (blue). Representative images of p-Ulk1^{746} (upper panels) and merged images (lower panels) are shown. **(B)** The population of cells displaying p-Ulk1^{746} signals was calculated ($n \geq 100$ cells). Data of cells treated with etoposide are shown as a positive control. WT no treatment vs. Rapamycin: p=0.9279. **(C, D)** Similar experiments to **(A, B)** were performed in starvation-treated *Atg5^{KO}* MEFs. In **(B, D)**, data are shown as the mean ± SD (n = 3). Exact p values cannot be described, since the value is too large (p>0.9999) or small (p<0.0001). Comparisons were performed using one-way ANOVA followed by the Tukey post-hoc test. **$p < 0.01$; NS: not significant.

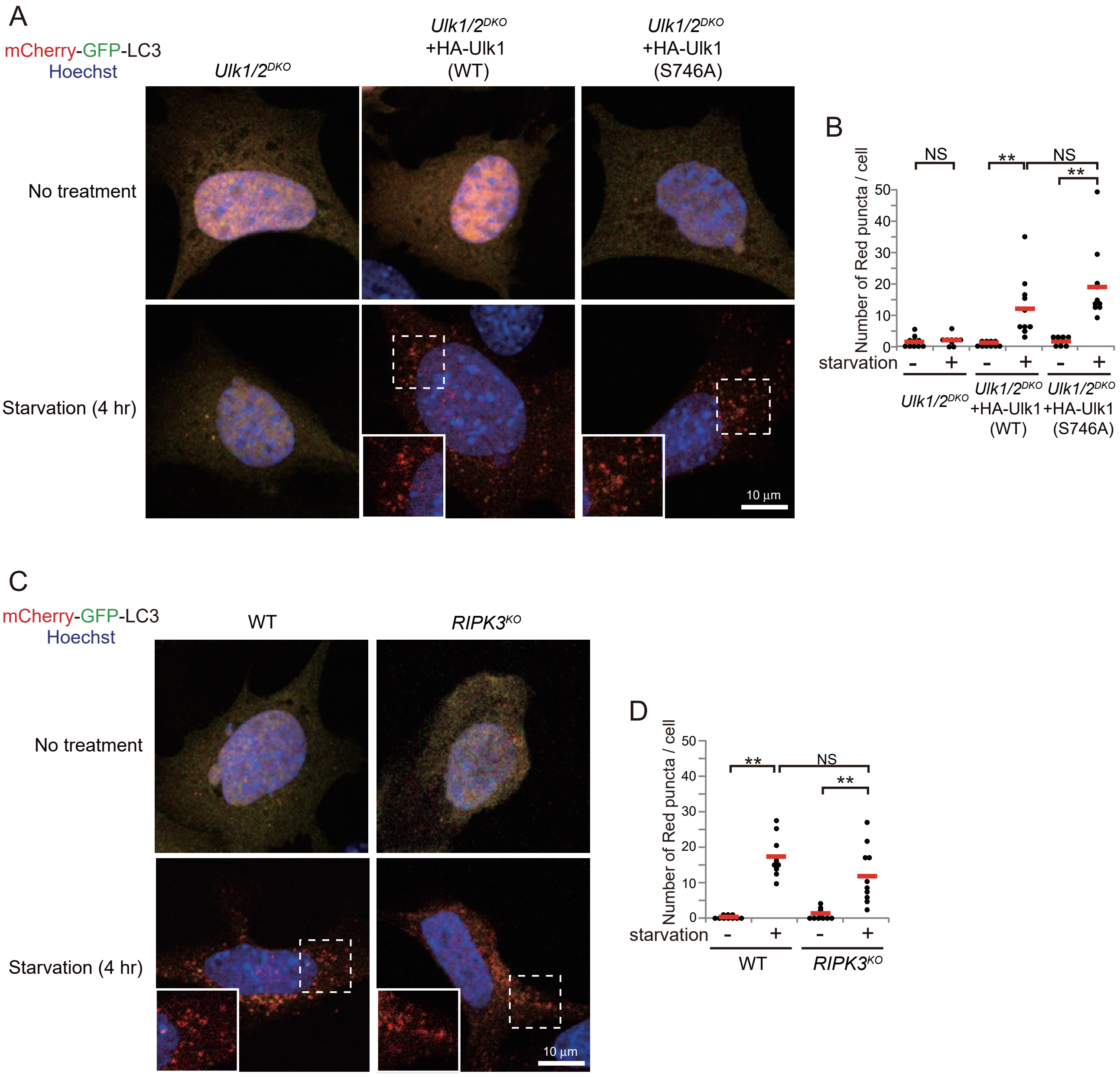

Supplementary Figure 12. Ulk1 Ser^{746} phosphorylation and RIPK3 are not involved in starvation-induced canonical autophagy. The indicated MEFs were transiently transfected with the mCherry-GFP-LC3 plasmid. After 24 hr, cells were treated with or without starvation for 4 hr. Nuclei were counterstained with Hoechst 33342. **(A, C)** Representative images are shown. Magnified images of the areas within the dashed squares are shown in the insets. Red puncta indicate autolysosomes. **(B, D)** The number of red puncta per cell was calculated ($n = 10$ cells in each experiment). Red lines indicate mean values. In (B), *Ulk1/2*^{DKO}+HA-Ulk1 (WT) no treatment vs. starvation: p=0.0011. *Ulk1/2*^{DKO}+HA-Ulk1 (WT) vs. *Ulk1/2*^{DKO}+HA-Ulk1 (S746A) starvation: p=0.2649. In (D), WT vs. *RIPK3*^{KO} starvation: p=0.128. Comparisons were performed using one-way ANOVA followed by the Tukey post-hoc test. $^{**}p < 0.01$. NS: not significant.

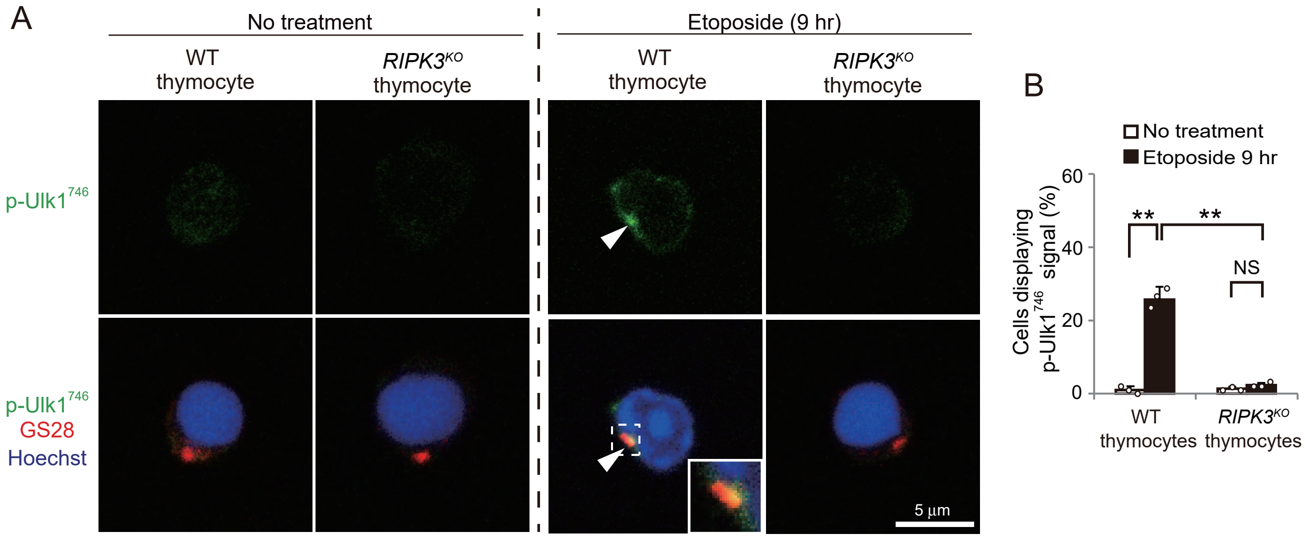

Supplementary Figure 13. Involvement of RIPK3 in the phosphorylation of Ulk1 Ser^{746} in etoposide-treated primary thymocytes. (A) WT and *RIPK3*^{KO} primary thymocytes were treated with or without etoposide (10 μM) for 9 hr, and immunostained with anti-p-Ulk1^{746} (green) and anti-GS28 (red) antibodies. Nuclei were counterstained with Hoechst 33342 (blue). Representative images of p-Ulk1^{746} (upper panels) and merged images (lower panels) are shown. Arrowheads indicate p-Ulk1^{746} signals. A magnified image of the area within the dashed square is shown in the inset. **(B)** The population of cells displaying p-Ulk1^{746} signals was calculated ($n \geq 100$ cells). Data are shown as the mean ± SD ($n = 3$). *RIPK3*^{KO} thymocytes no treatment vs. Etoposide: p=0.911. Comparisons were performed using one-way ANOVA followed by the Tukey post-hoc test. **$p < 0.01$. NS: not significant.

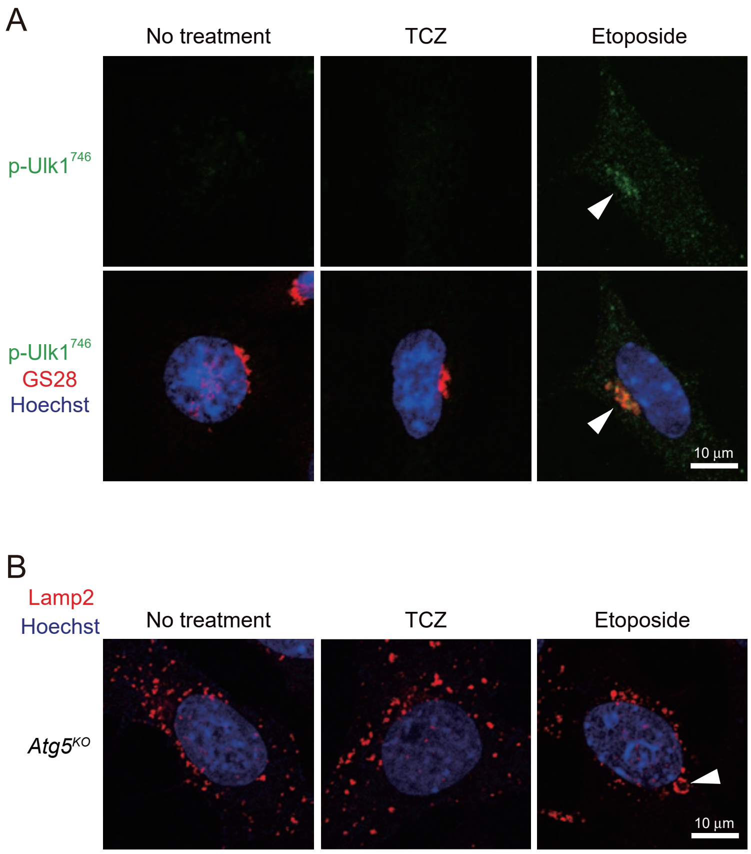

Supplementary Figure 14. TCZ does not induce the phosphorylation of Ulk1 at Ser^{746} and alternative autophagy. *Atg5^{KO}* MEFs were treated with or without the necroptosis-inducing TCZ solution for 9 hr, and then immunostained with anti-p-Ulk1^{746} (green) and anti-GS28 (red) antibodies **(A)** or an anti-Lamp2 antibody **(B)**. Nuclei were counterstained with Hoechst 33342. In **(A)**, representative images of p-Ulk1^{746} (upper panels) and merged images (lower panels) are shown. In **(B)**, representative images of Lamp2 puncta are shown. The image of cells treated with etoposide is shown as a positive control. Arrowheads indicate p-Ulk1^{746} signals **(A)** and large Lamp2 puncta **(B)**. From these images, cells undergoing alternative autophagy were calculated, and shown in Fig. 5B, C.

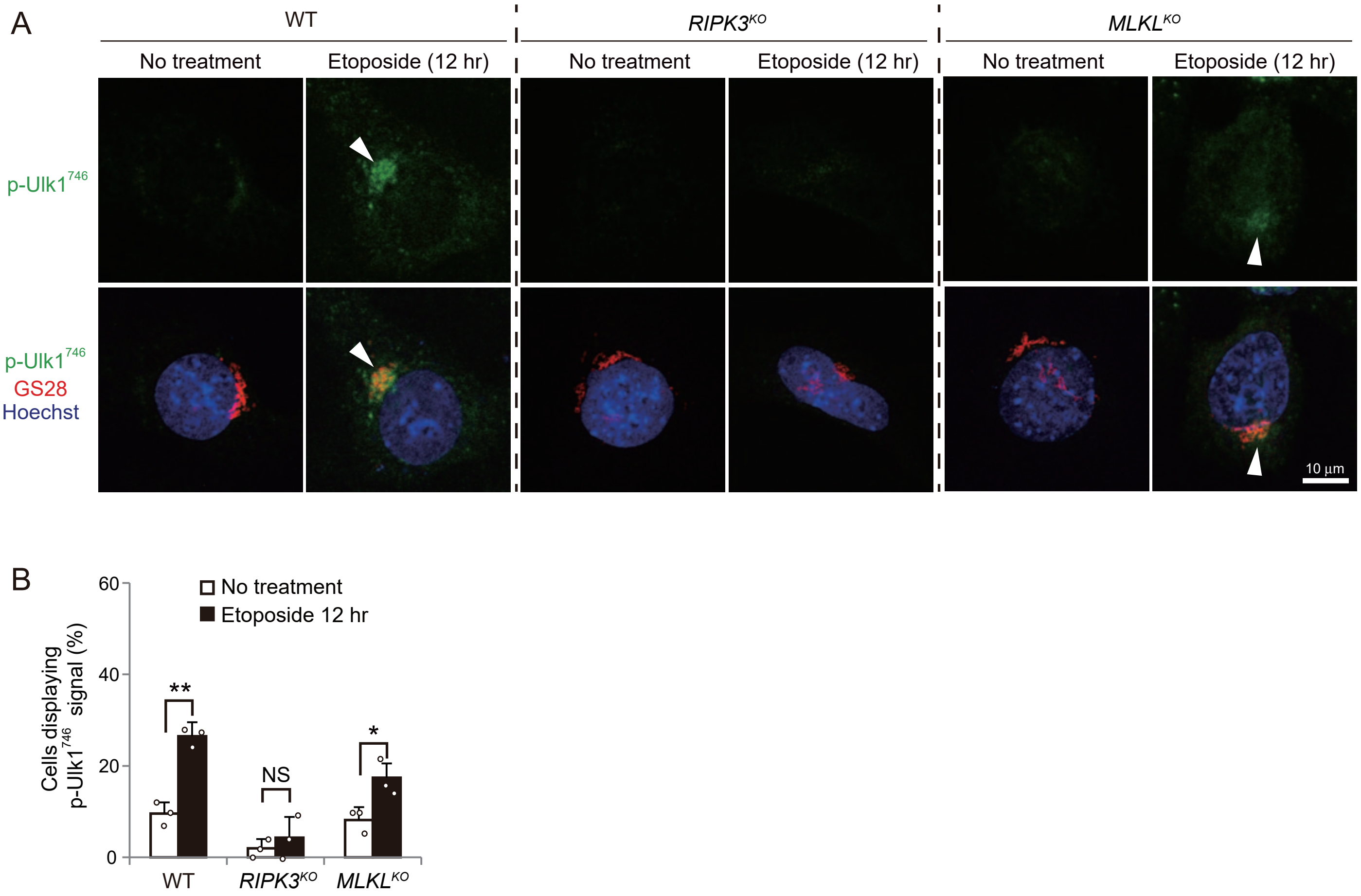

Supplementary Figure 15. Involvement of RIPK3, but not MLKL, in alternative autophagy. (A) The indicated MEFs were treated with or without etoposide (10 μM) for 12 hr, and immunostained with anti-p-Ulk1^{746} (green) and anti-GS28 (red) antibodies. Nuclei were counterstained with Hoechst 33342. Representative images of p-Ulk1^{746} (upper panels) and merged images (lower panels) are shown. Arrowheads indicate p-Ulk1^{746} signals. **(B)** The population of cells displaying p-Ulk1^{746} signals was calculated ($n \geq 100$ cells). Data are shown as the mean ± SD ($n = 3$). WT no treatment vs. Etoposide: p=0.0002, *RIPK3^{KO}* no treatment vs. Etoposide: p=0.9316, *MLKL^{KO}* no treatment vs. Etoposide: p=0.0295. Comparisons were performed using one-way ANOVA followed by the Tukey post-hoc test. *$p < 0.05$, **$p < 0.01$; NS: not significant.

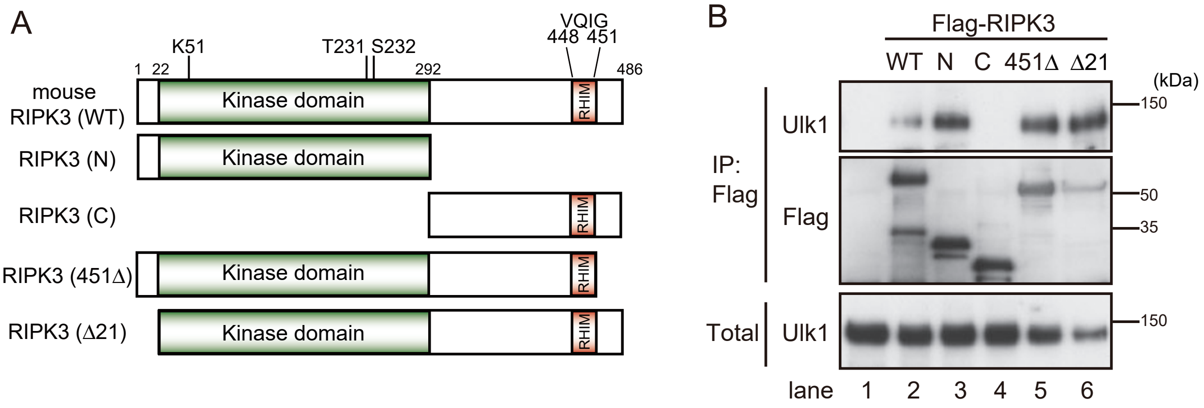

Supplementary Figure 16. Analysis of RIPK3 deletion mutants. (A) Diagram of the various RIPK3 deletion mutants designed and analyzed. **(B)** Flag-tagged RIPK3 deletion mutants were expressed in MEFs and immunoprecipitated using an anti-Flag antibody. Coimmunoprecipitation of Ulk1 was analyzed by western botting using an anti-Ulk1 antibody. The N-terminus region with kinase domain is required (lane 4), but the RHIM domain is not required (lane 3) for the RIPK3-Ulk1 interaction.

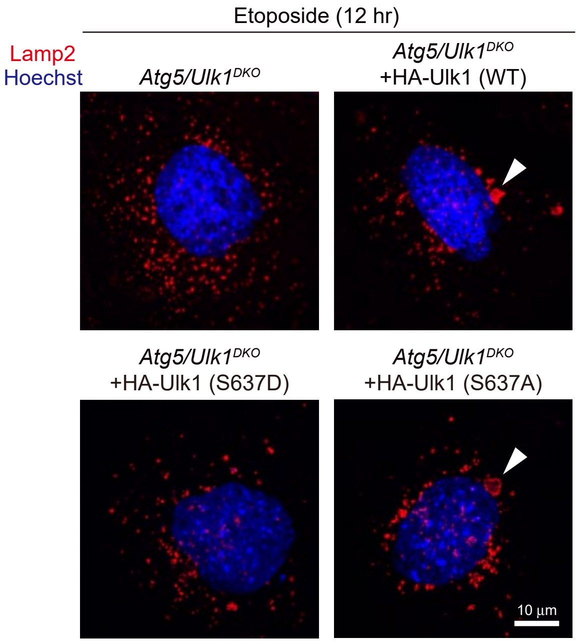

Supplementary Figure 17. Requirement of Ulk1 dephosphorylation at Ser^{637} for alternative autophagy. The indicated MEFs were treated with etoposide (10 µM) for 12 hr, and immunostained with an anti-Lamp2 antibody. Nuclei were counterstained with Hoechst 33342. Representative images are shown. Arrowheads indicate a large Lamp2 punctum. From these images, cells undergoing alternative autophagy were calculated, and shown in Fig. 7D.

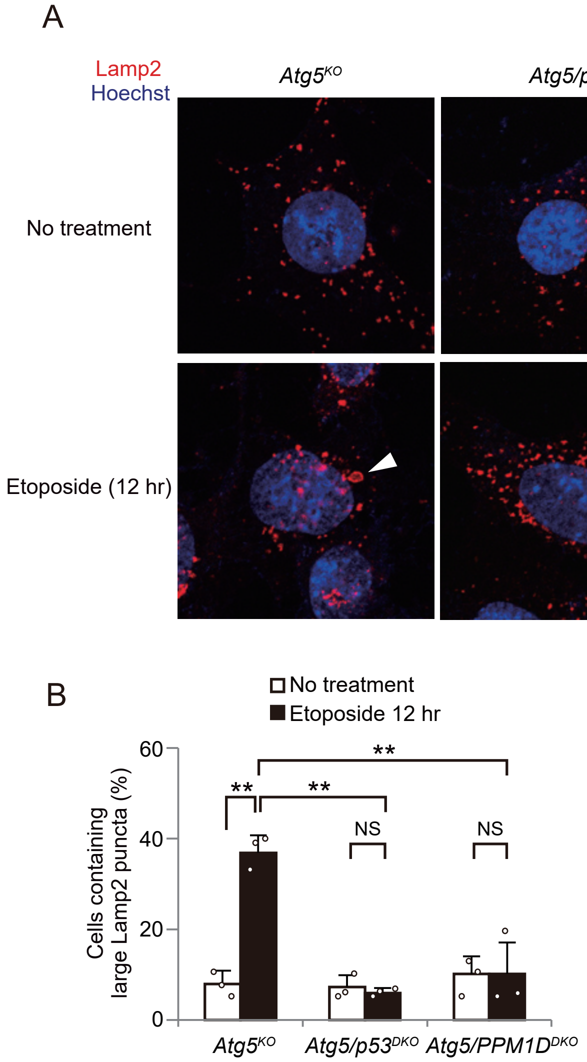

Supplementary Figure 18. Involvement of p53 and PPM1D in etoposide-induced alternative autophagy. The indicated MEFs were treated with or without etoposide (10 μM) for 12 hr, and immunostained with an anti-Lamp2 antibody. Nuclei were counterstained with Hoechst 33342. Representative images are shown in **(A)**. The arrowhead indicates a large Lamp2 punctum. **(B)** The percentage of cells containing large Lamp2 puncta was calculated ($n \geq 100$ cells). Data are shown as the mean ± SD ($n = 3$). $^{**}p < 0.01$. *Atg5/p53^{DKO}* no treatment vs. Etoposide: p=0.9991, *Atg5^{KO}* vs. *Atg5/PPM1D^{DKO}*, Etoposide: p=0.0001. Comparisons were performed using one-way ANOVA followed by the Tukey post-hoc test. NS: not significant.

Torii_Supplementary Figure 19

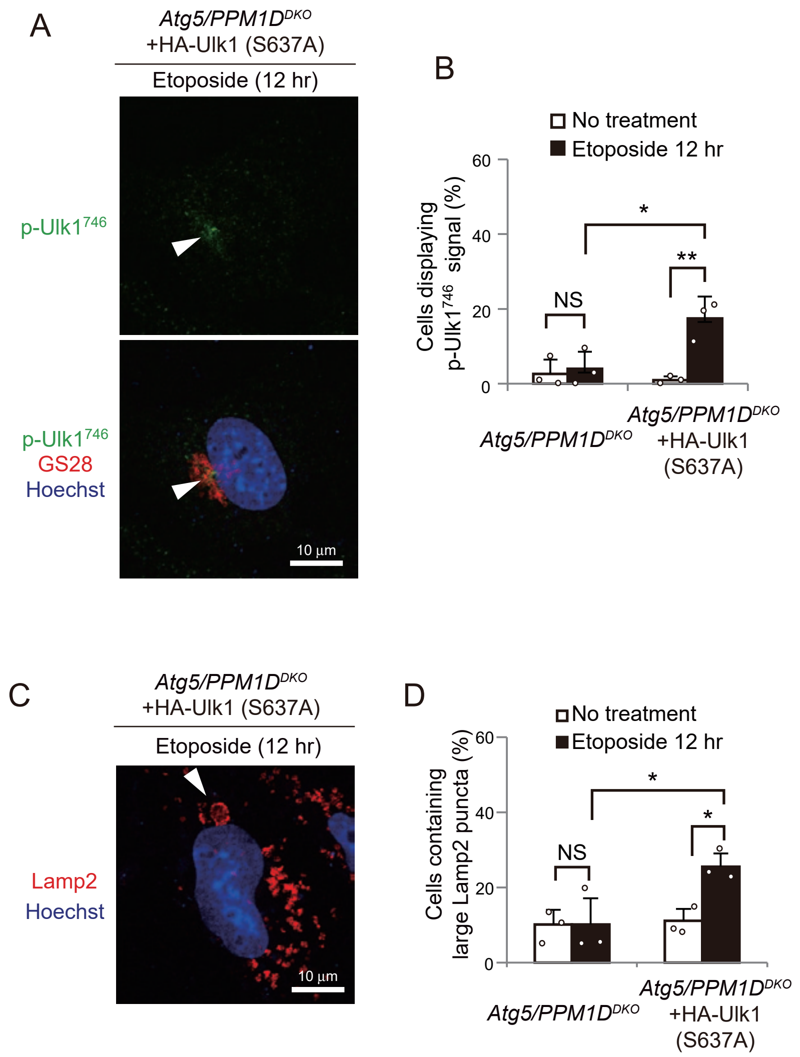

Supplementary Figure 19. No requirement of PPM1D for the phosphorylation of Ulk1 Ser^{746} and alternative autophagy in cells expressing dephosphorylated Ulk1^{637}. **(A)** *Atg5/PPM1D*^{DKO} MEFs stably expressing the HA-Ulk1 (S637A) mutant were treated with etoposide (10 μM) for 12 hr, and immunostained with anti-p-Ulk1^{746} (green) and anti-GS28 (red) antibodies. Representative images of p-Ulk1^{746} (upper panel) and merged images (lower panel) are shown. Arrowheads indicate p-Ulk1^{746} signals. **(B)** The population of cells displaying p-Ulk1^{746} signals was calculated ($n \geq 100$ cells). *Atg5/PPM1D*^{DKO} no treatment vs. Etoposide: p=0.9784, *Atg5/PPM1D*^{DKO}+HA-Ulk1 (S637A) no treatment vs. Etoposide: p=0.0057, *Atg5/PPM1D*^{DKO} vs. *Atg5/PPM1D*^{DKO}+HA-Ulk1 (S637A), Etoposide: p=0.0178. **(C)** *Atg5/PPM1D*^{DKO} MEFs stably expressing HA-Ulk1 (S637A) were treated with etoposide (10 μM) for 12 hr, and immunostained with an anti-Lamp2 antibody. The arrowhead indicates a large Lamp2 punctum. **(D)** The population of cells containing large Lamp2 puncta was calculated ($n \geq 100$ cells). *Atg5/PPM1D*^{DKO}+HA-Ulk1 (S637A) no treatment vs. Etoposide: p=0.0476, *Atg5/PPM1D*^{DKO} vs. *Atg5/PPM1D*^{DKO}+HA-Ulk1 (S637A), Etoposide: p=0.0355. In **(B)** and **(D)**, data are shown as the mean ± SD ($n = 3$). Comparisons were performed using one-way ANOVA followed by the Tukey post-hoc test. $^{**}p < 0.01$, $^{*}p < 0.05$; NS: not significant.

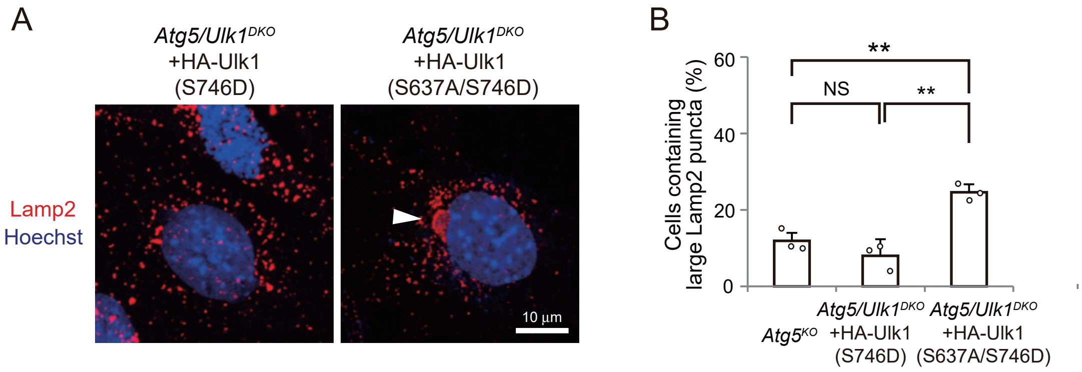

Supplementary Figure 20. Simple expression of Ulk1 (S637A/S746D) is sufficient to induce alternative autophagy. The indicated MEFs were generated and immunostained with an anti-Lamp2 antibody without any stimuli. Nuclei were counterstained with Hoechst 33342. **(A)** Representative images are shown. Arrowhead indicates large Lamp2 punctum. **(B)** The proportion of cells with large Lamp2 puncta was calculated ($n \geq 100$ cells). Data are shown as the mean ± SD ($n = 3$). $Atg5^{KO}$ vs. $Atg5/Ulk1^{DKO}$+HA-Ulk1 (S746D): p=0.312, $Atg5/Ulk1^{DKO}$+HA-Ulk1 (S746D) vs. $Atg5/Ulk1^{DKO}$+HA-Ulk1 (S637A/S746D): p=0.0013, $Atg5^{KO}$ vs. $Atg5/Ulk1^{DKO}$+HA-Ulk1 (S637A/S746D): p=0.0051. Comparisons were performed using one-way ANOVA followed by the Tukey post-hoc test. **$p < 0.01$; NS: not significant. Note that large Lamp2 puncta were generated by the simple expression of HA-Ulk1 (S637A/S746D) in $Atg5/Ulk1^{DKO}$ MEFs.

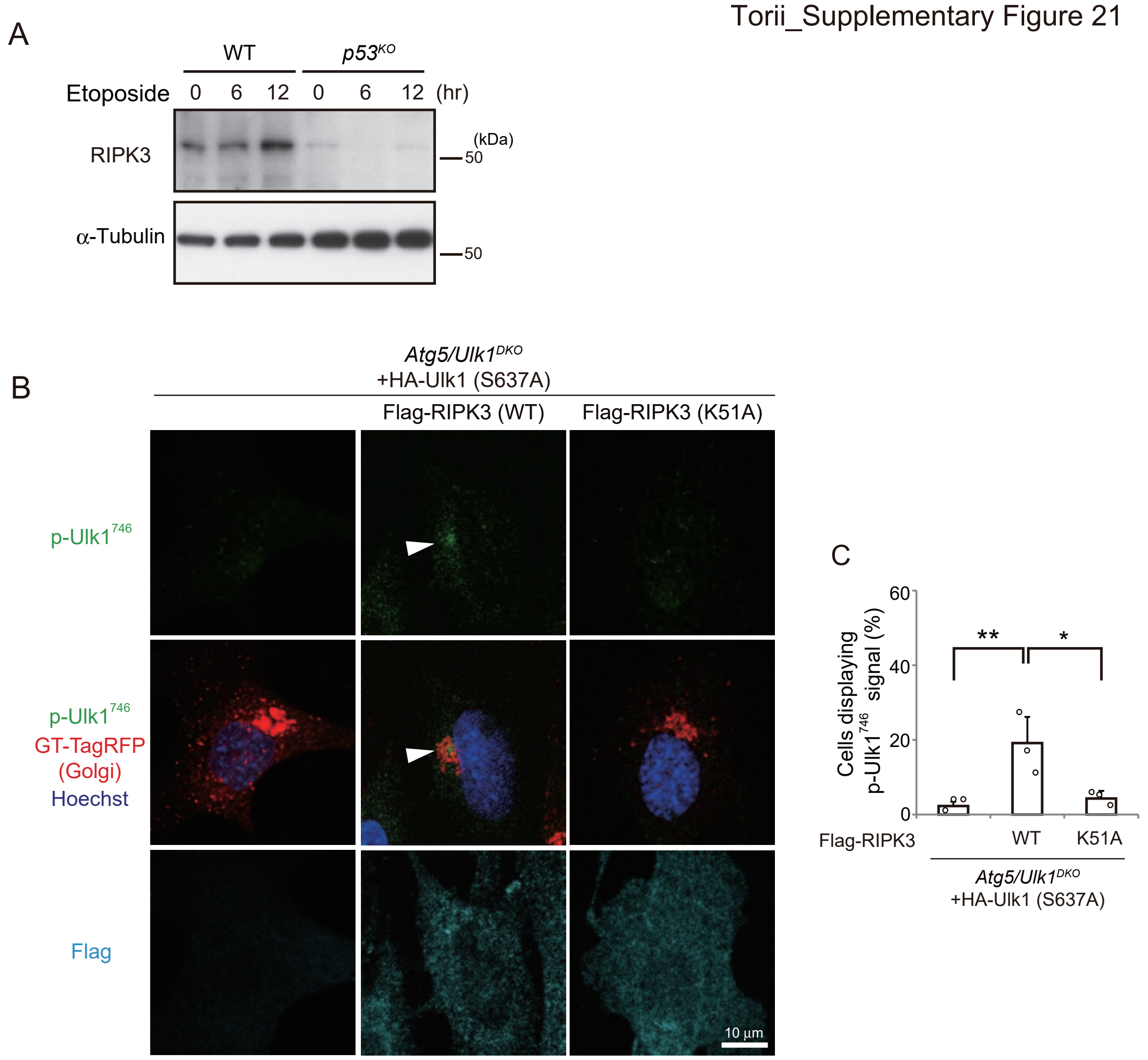

Supplementary Figure 21. Crucial role of p53 in RIPK3 expression. (A) The indicated MEFs were treated with etoposide (10 µM) for the indicated times. The expression of RIPK3 was analyzed by western blotting. α-Tubulin was included as a loading control. **(B)** HA-Ulk1 (S637A)-expressing *Atg5/Ulk1*^{DKO} MEFs were transfected with an expression vector for GT-TagRFP, together with expression vectors encoding Flag-RIPK3 (WT) or mutant Flag-RIPK3 (K51A). After 24 hr, cells were immunostained with anti-p-Ulk1^{746} (green) and anti-Flag (cyan) antibodies. Representative images of p-Ulk1^{746} (upper panels) and Flag (bottom panels) staining, and merged images (middle panels) are shown. Arrowheads indicate p-Ulk1^{746} signals. **(C)** The population of cells displaying p-Ulk1^{746} signals was calculated ($n \geq 100$ cells). Data are shown as the mean ± SD ($n = 3$). *Atg5/Ulk1*^{DKO}+HA-Ulk1 (S637A) mock vs. Flag-RIPK3 (WT): p=0.007, *Atg5/Ulk1*^{DKO}+HA-Ulk1 (S637A) Flag-RIPK3 (WT) vs. (K51A): p=0.0125, Comparisons were performed using one-way ANOVA followed by the Tukey post-hoc test. **$p < 0.01$, *$p < 0.05$.

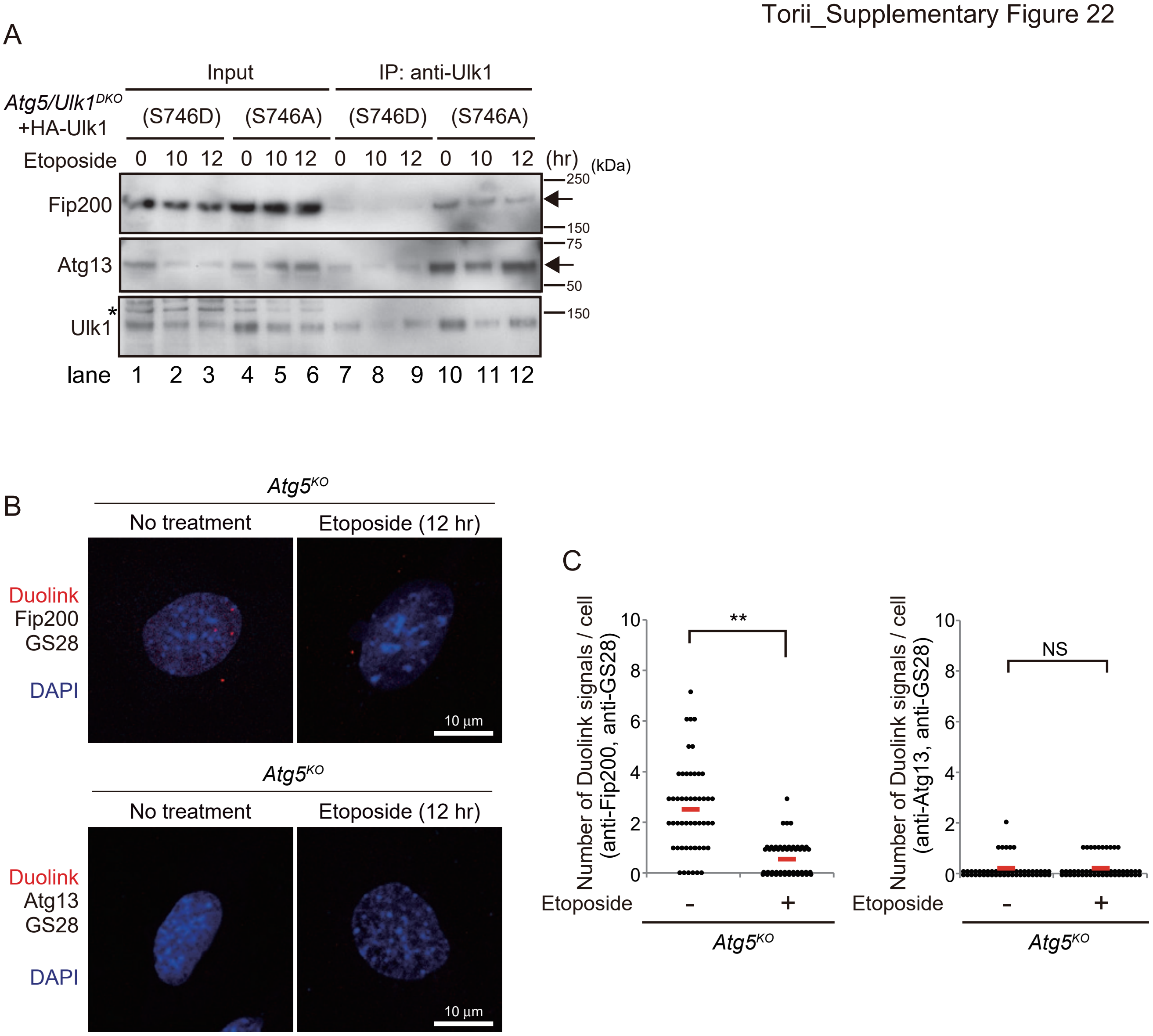

Supplementary Figure 22. Effects of Ulk1 phosphorylation at Ser^{746} on the interaction of Ulk1 with Fip200 and Atg13. (A) The indicated MEFs were treated with 10 µM of etoposide for the indicated times. Cells were then lysed and immunoprecipitated with an anti-Ulk1 antibody. Immune complexes and total lysates (5.6% input) were analyzed by western blotting using anti-Ulk1, anti-Fip200, and anti-Atg13 antibodies. The EasyBlot anti-rabbit IgG kit (GeneTex) was used to avoid the detection of nonspecific IgG bands. The asterisk indicates the non-specific band. **(B)** *Atg5^{KO}* MEFs were treated with or without etoposide (10 µM), and assayed with Duolink reagents using anti-Fip200/anti-GS28 antibodies (upper panels) or anti-Atg13/anti-GS28 antibodies (lower panels). Representative images are shown. In **(C)**, the number of Duolink-red signals were counted (n = 50 cells in each experiment). Red bars indicate mean values. p=5.1×10^{-10} (left), p=0.62 (right). Comparisons were performed using unpaired two-tailed Student t-tests. **$p < 0.01$; NS: not significant. Source data are provided as a Source Data file.

Torii_Supplementary Figure 23

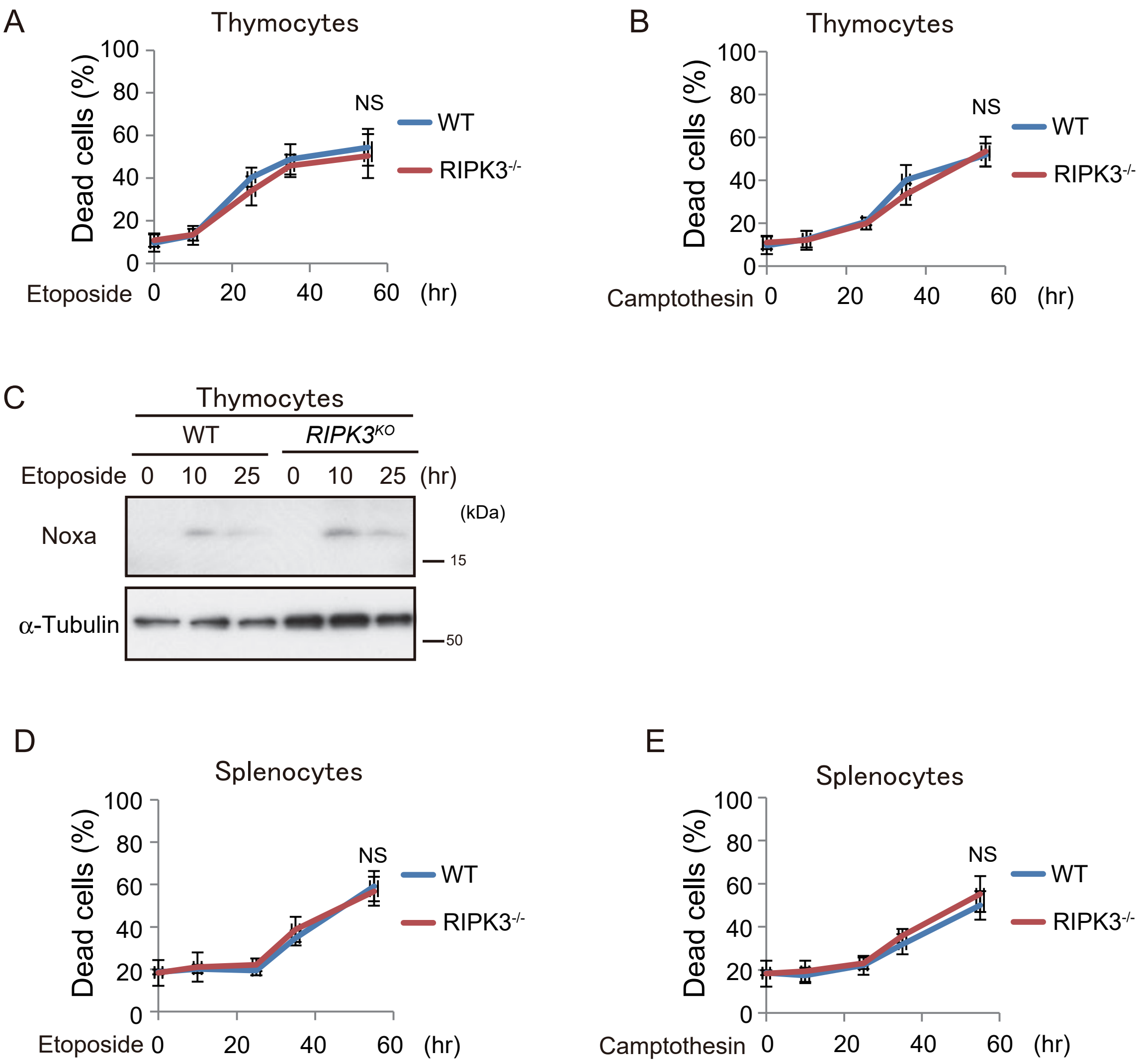

Supplementary Figure 23. No involvement of RIPK3-induced alternative autophagy in genotoxic stress-induced apoptosis. (A, B) The indicated thymocytes were treated with 10 μM of etoposide **(A)** or 20 μM of camptothecin **(B)**, and cell death was determined using the PI assay. **(C)** The indicated thymocytes were treated with 10 μM of etoposide**,** and lysates were collected at the indicated times. Noxa expression was analyzed by western blot analysis. Although canonical autophagy degrades Noxa and inhibits apoptosis, alternative autophagy did not affect Noxa expression. **(D, E)** Similar experiments with **(A, B)** were performed using splenocytes, instead of thymocytes. Data represent the mean ± SD ($n = 3$). In (A), WT vs.RIPK3 etoposide 55 hr: p=0.9978. In (C), WT vs.RIPK3 Etoposide 55 hr: p=0.9998. In (D), WT vs.RIPK3 Camptothecin 55 hr: p=0.9419, Comparisons were performed using one-way ANOVA followed by the Tukey post-hoc test. NS: not significant.

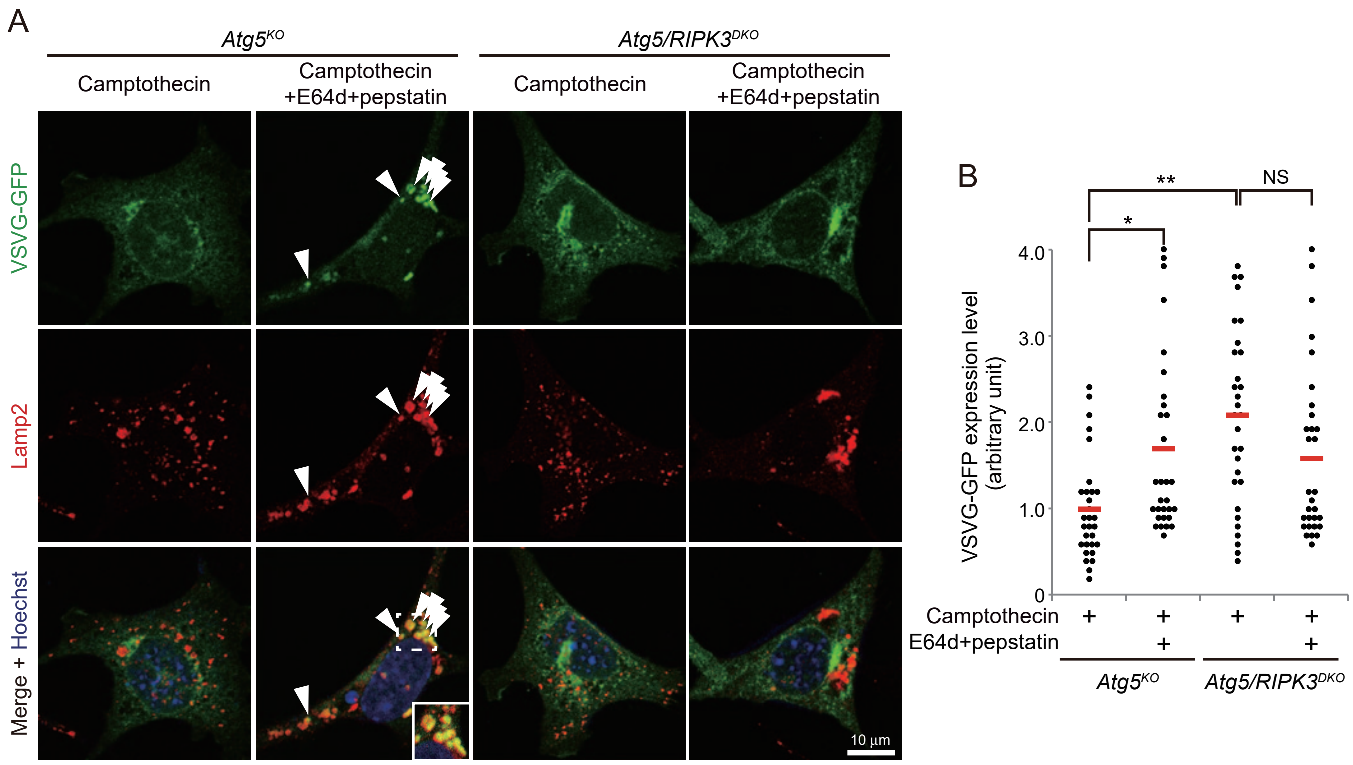

Supplementary Figure 24. Effects of RIPK3 on VSVG trafficking upon camptothecin treatment. Similar experiments to Fig. 9D and 9E were performed using camptothecin, instead of etoposide. VSVG–GFP-expressing indicted MEFs were treated with camptothecin (20 μM) in the presence of E64d/pepstatin. At 60 min after the temperature shift from the restrictive temperature (40 °C) to the permissive temperature (32 °C), cells were immunostained with an anti-Lamp2 antibody. In **(A)**, representative images are shown. Arrowheads indicate autolysosomes containing VSVG–GFP. A magnified image of the areas within the dashed squares is shown in the insets. In **(B)**, the amount of total VSVG–GFP (the level of fluorescence intensity in each cell) was measured using Image J (n = 30 cells in each experiment). Red bars indicate mean values. *$Atg5^{KO}$* Camptothecin vs. Camptothecin with E64d/pepstatin: p=0.0297, *$Atg5/RIPK3^{DKO}$* Camptothecin vs. Camptothecin with E64d/pepstatin: p=0.2407, *$Atg5^{KO}$* vs. *$Atg5/RIPK3^{DKO}$*, Camptothecin: p=0.0001. Comparisons were performed using one-way ANOVA followed by the Tukey post-hoc test. *$p < 0.05$, **$p < 0.01$; NS: not significant.

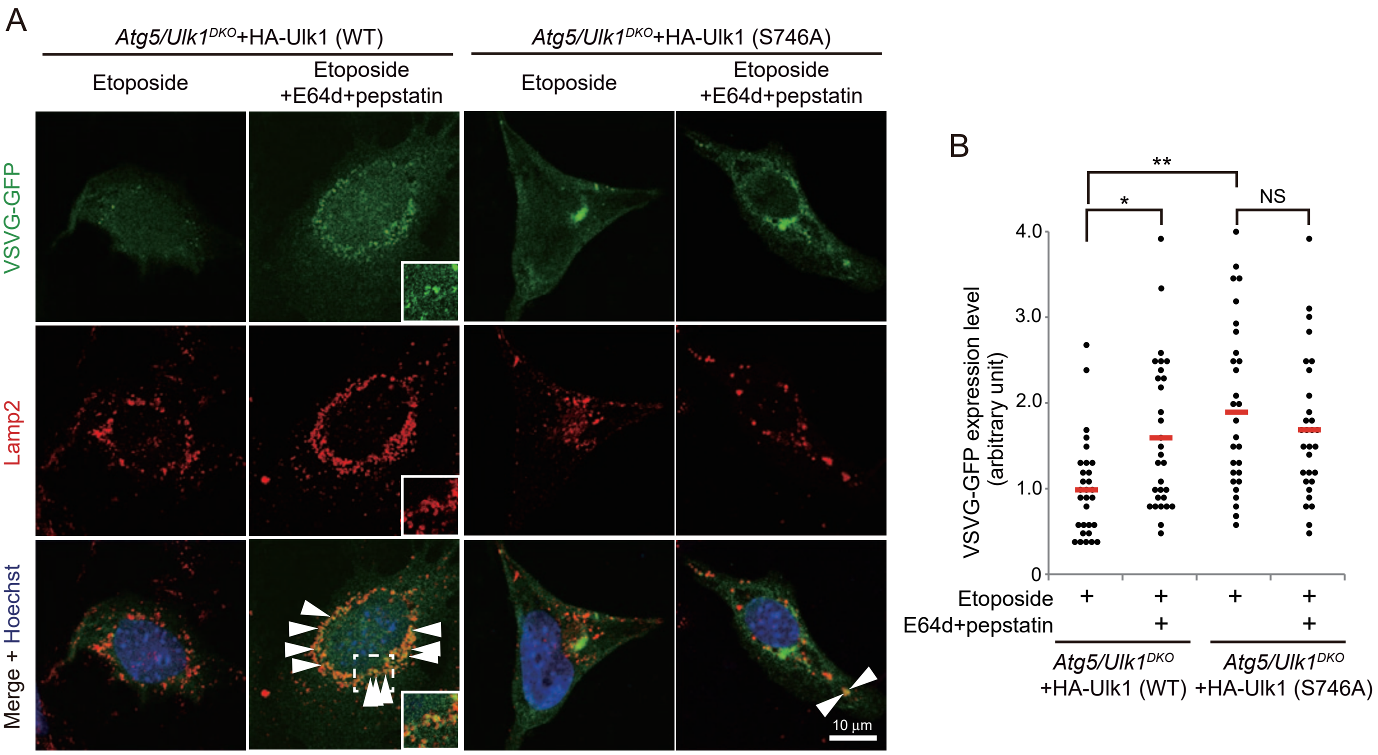

Supplementary Figure 25 Effects of Ulk1 phosphorylation on the degradation of undelivered VSVG upon etoposide treatment. Similar experiments to Fig. 9D and 9E were performed using *Atg5/Ulk1^{DKO}* MEF derivatives. In **(A)**, representative images are shown. Arrowheads indicate autolysosomes containing VSVG–GFP. A magnified image of the areas within the dashed squares is shown in the insets. In **(B)**, the amount of total VSVG–GFP (the level of fluorescence intensity in each cell) was measured using Image J (n = 30 cells in each experiment). Red bars indicate mean values. *Atg5/Ulk1^{DKO}*+HA-Ulk1 (WT) etoposide vs. etoposide with E64d/pepstatin: p=0.0268, *Atg5/Ulk1^{DKO}*+HA-Ulk1 (S746A) etoposide vs. etoposide with E64d/pepstatin: p=0.7063, *Atg5/Ulk1^{DKO}*+HA-Ulk1 (WT) vs. *Atg5/Ulk1^{DKO}*+HA-Ulk1 (S746A), Etoposide: p=0.0002. Comparisons were performed using one-way ANOVA followed by the Tukey post-hoc test. *$p < 0.05$, **$p < 0.01$; NS: not significant.

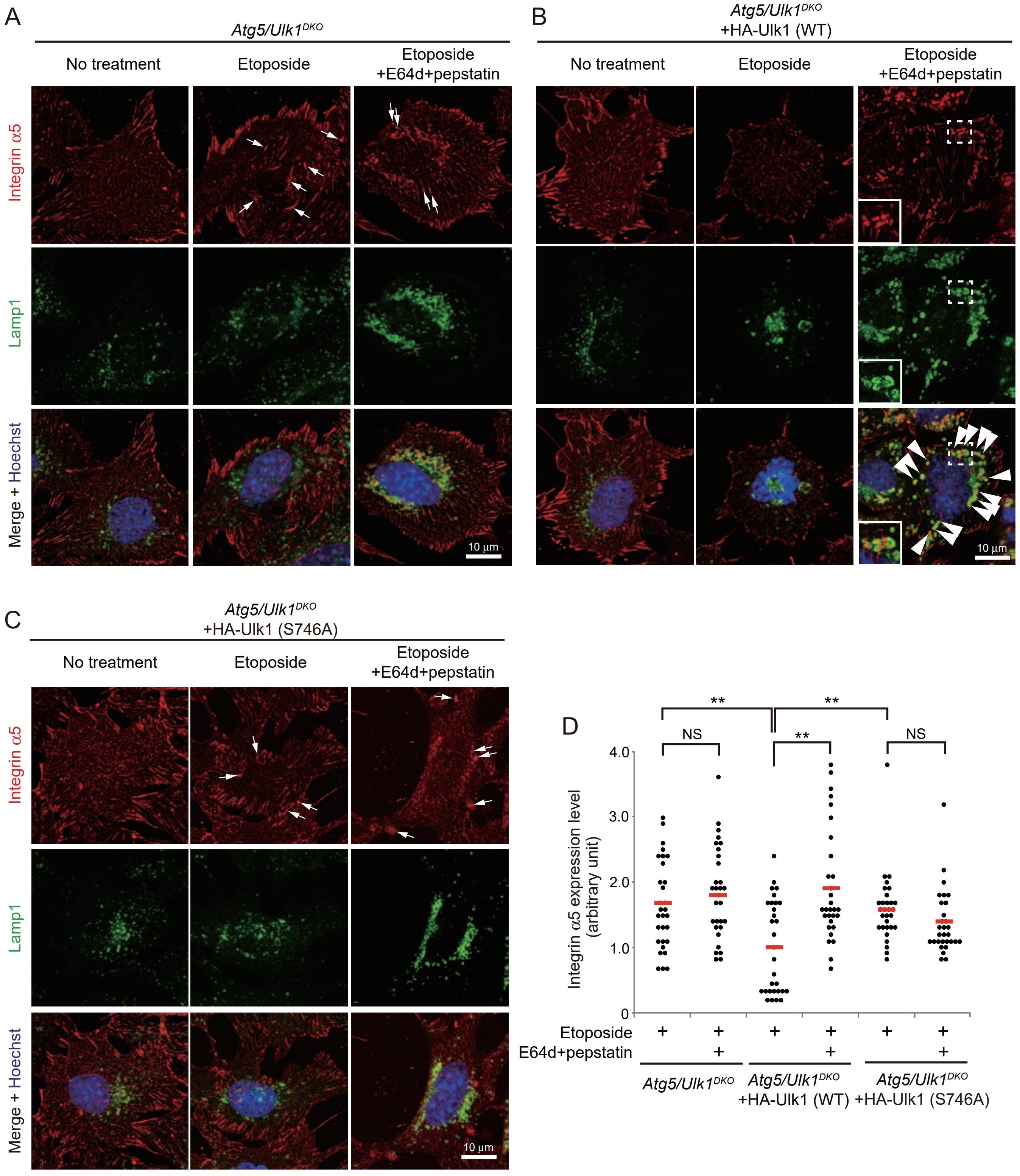
A
Atg5/Ulk1DKO
No treatment
Etoposide
Etoposide +E64d+pepstatin
Integrin α5
Lamp1
Merge + Hoechst
10 μm
B
Atg5/Ulk1DKO +HA-Ulk1 (WT)
C
Atg5/Ulk1DKO +HA-Ulk1 (S746A)
D
Integrin α5 expression level (arbitrary unit)
4.0
3.0
2.0
1.0
0
**
NS
Etoposide
E64d+pepstatin
+
Atg5/Ulk1DKO
Atg5/Ulk1DKO +HA-Ulk1 (WT)
Atg5/Ulk1DKO +HA-Ulk1 (S746A)

Supplementary Figure 26. Requirement of Ulk1 phosphorylation in undelivered integrin α5 degradation upon etoposide treatment. Similar experiments to Fig. 10 were performed using *Atg5/Ulk1*^{DKO} MEFs and their derivatives. In **(A-C)**, representative images are shown. Arrows and arrowheads indicate unusual cytoplasmic integrin alpha5 puncta and integrin α5 engulfed in autolysosomes, respectively. Magnified images of the areas within the dashed squares are shown in the insets. Note that etoposide generated unusual cytoplasmic integrin alpha5 puncta in *Atg5/Ulk1*^{DKO} MEFs and *Atg5/Ulk1*^{DKO} MEFs expressing HA-Ulk1 (S746A). In **(D)**, the extent of total integrin α5 (the level of fluorescence intensity per cell) was measured (n = 30 cells in each experiment). Red bars indicate mean values. *Atg5/Ulk1*^{DKO} etoposide vs. etoposide with E64d/pepstatin: p=0.9063, *Atg5/Ulk1*^{DKO}+HA-Ulk1 (S746A) etoposide vs. etoposide with E64d/pepstatin: p=0.805, *Atg5/Ulk1*^{DKO} vs. *Atg5/Ulk1*^{DKO}+HA-Ulk1 (WT), Etoposide: p=0.0033, *Atg5/Ulk1*^{DKO}+HA-Ulk1 (WT) vs. *Atg5/Ulk1*^{DKO}+HA-Ulk1 (S746A) Etoposide: p=0.0099. Comparisons were performed using one-way ANOVA followed by the Tukey post-hoc test. **p < 0.01; NS: not significant. Source data are provided as a Source Data file.

Torii_Supplementary Figure 27

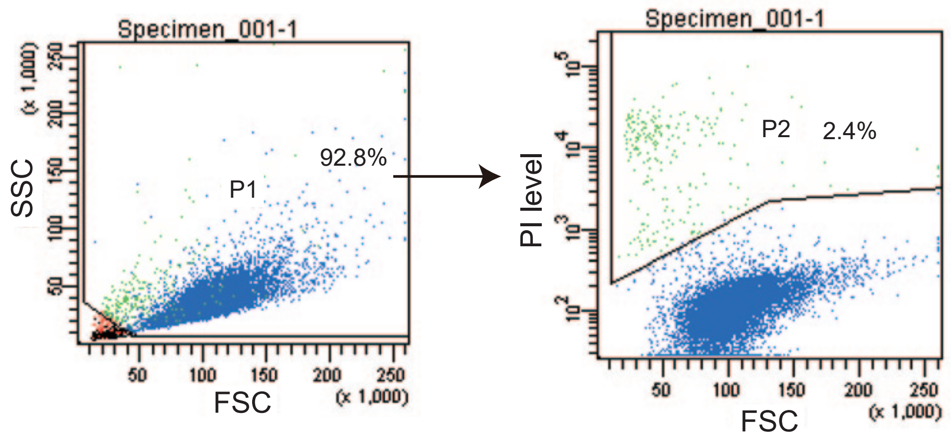

Supplementary Figure 27. Flow Cytometry gating strategies. For cell viability assay, cellular debris was removed using FSC/SSC (left panel). Then, PI-positive dead cells were detected using FSC/PI (right panel).

Supplementary Table 1. Antibody list

name	Campany	Cat. Number	method	dilution
phospho-Ulk1 (S746)	self-produced in this paper (Eurofins)		IP, IF	1:100, 1:400
Ulk1	Sigma-Aldrich	A7481	WB, Duolink	1:2,000, 1:100
GS28 clone11	BD Biosciences	611184	IF, Duolink	1:200, 1:200
Lamp2 GL2A7	Abcam	ab13524	IF	1:200
RIPK3	Cell Signaling Technology	95702	WB, Duolink	1:1000, 1:100
phospho-RIPK3 (T231/S232)	Cell Signaling Technology	57220	WB	1:1000
PPM1D (Wip1)	Cell Signaling Technology	11901	WB	1:200
phospho-Ulk1 (S638)	Cell Signaling Technology	12097	WB	1:500
Atg5	Sigma-Aldrich	A0731	WB	1:1,000
p53 PAB421	Calbiochem	OP03-20	WB	1:200
α-Tubulin DM1A	Sigma-Aldrich	T9026	WB	1:3,000
LC3 5F10	nanotools	5F10	WB	1:1,000
LC3 1703	Cosmobio	CTB-LC3-2-IC	IF	1:100
p62	MBL	PM045	WB	1:1000
HA F-7	Santa Cruz	sc-7392	WB	1:200
phopho-MLKL	Cell Signaling Technology	62233	WB	1:1,000
VSVG 8G5F11	KeraFAS	EB0010	FACS	1:100
FLAG M2	Sigma-Aldrich	F1804	IP, IF	1:60, 1:100
DDDDK-tag	MBL	PM020	WB	1:500
Fip200 D10D11	Cell Signaling Technology	12436	WB, Duolink	1:1000, 1:100
Atg13 D4P1K	Cell Signaling Technology	13273	WB, Duolink	1:1000, 1:100
Noxa 114C307.1	Novus	NB600-1159	WB	1:500
Integrin alpha 5 antibody [5H10-27]	Abcam	ab25461	IF	1:100
Lamp1	Abcam	ab24170	IF	1:200

Supplementary Table 2. Used primers

Names	Sequences
mouse Ulk1 S317A-Fw	GGCCTCTCCACCGGCCCTGGGGGAGATGCC
mouse Ulk1 S317A-Rv	GGCATCTCCCCCAGGGCCGGTGGAGAGGCC
mouse Ulk1 S494A-Fw	CTGGCCAGGAAGCTGGCACTTGGAGGTGGC
mouse Ulk1 S494A-Rv	GCCACCTCCAAGTGCCAGCTTCCTGGCCAG
mouse Ulk1 S746A-Fw	GTGGTGGAGGGGCCGCCAGCCCAGCACCTG
mouse Ulk1 S746A-Rv	CAGGTGCTGGGCTGGCGGCCCCTCCACCAC
mouse Ulk1 S746D-Fw	GTGGTGGAGGGGCCGACAGCCCAGCACCTG
mouse Ulk1 S746D-Rv	CAGGTGCTGGGCTGTCGGCCCCTCCACCAC
mouse Ulk1 S637A-Fw	CAAAACCCCCGCCTCTCAGAATTTGCTGAC
mouse Ulk1 S637A-Rv	GTCAGCAAATTCTGAGAGGCGGGGGTTTTG
mouse Ulk1 S637D-Fw	CAAAACCCCCGACTCTCAGAATTTGCTGAC
mouse Ulk1 S637D-Rv	GTCAGCAAATTCTGAGAGTCGGGGGTTTTG
mouse RIPK3-Fw-EcoRI	GATCGAATTCATGTCTTCTGTCAAGTTATG
mouse RIPK3-292aa(876)-Rv-stop-XhoI	GATCCTCGAGGTAAACTTCATTGGTTTTTG
mouse RIPK3-293aa(877)-C-Fw-EcoRI	GATCGAATTCAATCTGGTAAAGGACAAGGT
mouse RIPK3-451aa(1353)-Rv-stop-XhoI	GATCCTCGAGCCCAATCTGCACTTCAGAAC
mouse RIPK3-22aa(64)-Fw-EcoRI	GATCGAATTCCTGAAGAAGCTGGAGTTTGT
mouse RIPK3-Rv-stop-XhoI	GATCCTCGAGCTTGTGGAAGGGCTGCCAGC
Ulk1 Genotyping 1	CCTCCTACGTGCCCAGATG
Ulk1 Genotyping 2	AGCTTTCCCATCATCACAGG
Ulk1 Genotyping 3	ATAAACCCTCTTGCAGTTGCATC
PPM1D Genotyping 1	GTGGAGCTATGATTTCTTCAGTGG
PPM1D Genotyping 2	GATACGACACAAGACAAACCTCC
PPM1D Genotyping 3	ACAAGCTTGCAGGGCTGTTTGTGG
PPM1D Genotyping 4	CTTCCCAGCCTCTGAGCCCAGAAAGC
RIPK3 Genotyping 1	CGCTTTAGAAGCCTTCAGGTTGAC
RIPK3 Genotyping 2	GCCTGCCCATCAGCAACTC
RIPK3 Genotyping 3	CCAGAGGCCACTTGTGTAGCG
Atg5 Genotyping 1	GAATATGAAGGCACACCCCTGAAATG
Atg5 Genotyping 2	ACAACGTCGAGCACAGCTGCGCAAGG
Atg5 Genotyping 3	GTACTGCATAATGGTTTAACTCTTGC
p53 Genotyping 1	ACACACCTGTAGCTCCAGCAC
p53 Genotyping 2	GTGTTCCGGCTGTCAGCGCA
p53 Genotyping 3	AGCGTCTCACGAACCTCCGTC
RIPK3-RT-PCR-Fw	TGTCAAGTTATGGCCTACTGGTGCG
RIPK3-RT-PCR-Rv	AACCATAGCCTTCACCTCCCAGGAT
18 S rRNA-RT-PCR-Fw	GGTCTGTGATGCCCTTAGATGTCC
18 S rRNA-RT-PCR-Rv	GTTCGACCGTCTTCTCAGCGCT
