# Supplementary material for: Identification of a phosphorylation site on Ulk1 required for genotoxic stress-induced alternative autophagy
Source: Nat Commun. 2020 Apr 9;11:1754. doi: 10.1038/s41467-020-15577-2 (PMC7145817; doi:10.1038/s41467-020-15577-2)
Supplement: Supplementary file 3 — Reporting Summary [file 41467_2020_15577_MOESM3_ESM.pdf]

nature research

Corresponding author(s): Satoru Torii, Shigeomi Shimizu

Last updated by author(s): Mar 11, 2020

Reporting Summary

Nature Research wishes to improve the reproducibility of the work that we publish. This form provides structure for consistency and transparency in reporting. For further information on Nature Research policies, see Authors & Referees and the Editorial Policy Checklist.

Statistics

For all statistical analyses, confirm that the following items are present in the figure legend, table legend, main text, or Methods section.

n/a	Confirmed	
☐	☒	The exact sample size (*n*) for each experimental group/condition, given as a discrete number and unit of measurement
☐	☒	A statement on whether measurements were taken from distinct samples or whether the same sample was measured repeatedly
☐	☒	The statistical test(s) used AND whether they are one- or two-sided<br>*Only common tests should be described solely by name; describe more complex techniques in the Methods section.*
☒	☐	A description of all covariates tested
☒	☐	A description of any assumptions or corrections, such as tests of normality and adjustment for multiple comparisons
☐	☒	A full description of the statistical parameters including central tendency (e.g. means) or other basic estimates (e.g. regression coefficient) AND variation (e.g. standard deviation) or associated estimates of uncertainty (e.g. confidence intervals)
☐	☒	For null hypothesis testing, the test statistic (e.g. *F*, *t*, *r*) with confidence intervals, effect sizes, degrees of freedom and *P* value noted<br>*Give P values as exact values whenever suitable.*
☒	☐	For Bayesian analysis, information on the choice of priors and Markov chain Monte Carlo settings
☒	☐	For hierarchical and complex designs, identification of the appropriate level for tests and full reporting of outcomes
☒	☐	Estimates of effect sizes (e.g. Cohen's *d*, Pearson's *r*), indicating how they were calculated

Our web collection on statistics for biologists contains articles on many of the points above.

Software and code

Policy information about availability of computer code

Data collection	Zen software 2012 (Zeiss), CFX manager 3.1 (Bio-Rad), BD FACSDiva
Data analysis	Zen software 2012 (Zeiss), Adobe photoshop CS5.1, Adobe Illustrator CS5.1, ImageJ, Excel, Prism 8 (GraphPad), BD FACSDiva, FlowJo software

For manuscripts utilizing custom algorithms or software that are central to the research but not yet described in published literature, software must be made available to editors/reviewers. We strongly encourage code deposition in a community repository (e.g. GitHub). See the Nature Research guidelines for submitting code & software for further information.

Data

Policy information about availability of data

All manuscripts must include a data availability statement. This statement should provide the following information, where applicable:

- Accession codes, unique identifiers, or web links for publicly available datasets
- A list of figures that have associated raw data
- A description of any restrictions on data availability

The data that support the findings of this study are available from the corresponding author upon request

Field-specific reporting

Please select the one below that is the best fit for your research. If you are not sure, read the appropriate sections before making your selection.

☒ Life sciences ☐ Behavioural & social sciences ☐ Ecological, evolutionary & environmental sciences

Life sciences study design

All studies must disclose on these points even when the disclosure is negative.

Sample size	We did not perform statistical analyses to predetermine sample sizes. Sample sizes were based on empirical data from pilot experiments and similar published our studies (Torii et al., 2016, doi: 10.15252/embr.201642565, Yamaguchi et al., 2016, doi: 10.15252/embj.201593191, Watanabe et al., 2016, doi: 10.1038/ncomms13508). Sample sizes are described in Methods and figure legends.
Data exclusions	No data was excluded from analysis.
Replication	Immunoblot analyses were conducted at least in duplicate. For each representative image, each experiment was repeated at least three times under similar condition. The number of replicates for each experiments is clearly presented in the figure legends.
Randomization	A batch of identical cultured cells were randomly allocated to treatment groups and control groups. Animals were randomly put into cages and randomly allocated to experimental groups.
Blinding	Blinding was not possible because the experiments were performed by a single researcher.

Behavioural & social sciences study design

All studies must disclose on these points even when the disclosure is negative.

Study description	*Briefly describe the study type including whether data are quantitative, qualitative, or mixed-methods (e.g. qualitative cross-sectional, quantitative experimental, mixed-methods case study).*
Research sample	*State the research sample (e.g. Harvard university undergraduates, villagers in rural India) and provide relevant demographic information (e.g. age, sex) and indicate whether the sample is representative. Provide a rationale for the study sample chosen. For studies involving existing datasets, please describe the dataset and source.*
Sampling strategy	*Describe the sampling procedure (e.g. random, snowball, stratified, convenience). Describe the statistical methods that were used to predetermine sample size OR if no sample-size calculation was performed, describe how sample sizes were chosen and provide a rationale for why these sample sizes are sufficient. For qualitative data, please indicate whether data saturation was considered, and what criteria were used to decide that no further sampling was needed.*
Data collection	*Provide details about the data collection procedure, including the instruments or devices used to record the data (e.g. pen and paper, computer, eye tracker, video or audio equipment) whether anyone was present besides the participant(s) and the researcher, and whether the researcher was blind to experimental condition and/or the study hypothesis during data collection.*
Timing	*Indicate the start and stop dates of data collection. If there is a gap between collection periods, state the dates for each sample cohort.*
Data exclusions	*If no data were excluded from the analyses, state so OR if data were excluded, provide the exact number of exclusions and the rationale behind them, indicating whether exclusion criteria were pre-established.*
Non-participation	*State how many participants dropped out/declined participation and the reason(s) given OR provide response rate OR state that no participants dropped out/declined participation.*
Randomization	*If participants were not allocated into experimental groups, state so OR describe how participants were allocated to groups, and if allocation was not random, describe how covariates were controlled.*

Ecological, evolutionary & environmental sciences study design

All studies must disclose on these points even when the disclosure is negative.

Study description	*Briefly describe the study. For quantitative data include treatment factors and interactions, design structure (e.g. factorial, nested, hierarchical), nature and number of experimental units and replicates.*
Research sample	*Describe the research sample (e.g. a group of tagged Passer domesticus, all Stenocereus thurberi within Organ Pipe Cactus National Monument), and provide a rationale for the sample choice. When relevant, describe the organism taxa, source, sex, age range and any manipulations. State what population the sample is meant to represent when applicable. For studies involving existing datasets, describe the data and its source.*
Sampling strategy	*Note the sampling procedure. Describe the statistical methods that were used to predetermine sample size OR if no sample-size calculation was performed, describe how sample sizes were chosen and provide a rationale for why these sample sizes are sufficient.*
Data collection	*Describe the data collection procedure, including who recorded the data and how.*

Timing and spatial scale	*Indicate the start and stop dates of data collection, noting the frequency and periodicity of sampling and providing a rationale for these choices. If there is a gap between collection periods, state the dates for each sample cohort. Specify the spatial scale from which the data are taken*
Data exclusions	*If no data were excluded from the analyses, state so OR if data were excluded, describe the exclusions and the rationale behind them, indicating whether exclusion criteria were pre-established.*
Reproducibility	*Describe the measures taken to verify the reproducibility of experimental findings. For each experiment, note whether any attempts to repeat the experiment failed OR state that all attempts to repeat the experiment were successful.*
Randomization	*Describe how samples/organisms/participants were allocated into groups. If allocation was not random, describe how covariates were controlled. If this is not relevant to your study, explain why.*
Blinding	*Describe the extent of blinding used during data acquisition and analysis. If blinding was not possible, describe why OR explain why blinding was not relevant to your study.*

Did the study involve field work? ☐ Yes ☐ No

Field work, collection and transport

Field conditions	*Describe the study conditions for field work, providing relevant parameters (e.g. temperature, rainfall).*
Location	*State the location of the sampling or experiment, providing relevant parameters (e.g. latitude and longitude, elevation, water depth).*
Access and import/export	*Describe the efforts you have made to access habitats and to collect and import/export your samples in a responsible manner and in compliance with local, national and international laws, noting any permits that were obtained (give the name of the issuing authority, the date of issue, and any identifying information).*
Disturbance	*Describe any disturbance caused by the study and how it was minimized.*

Reporting for specific materials, systems and methods

We require information from authors about some types of materials, experimental systems and methods used in many studies. Here, indicate whether each material, system or method listed is relevant to your study. If you are not sure if a list item applies to your research, read the appropriate section before selecting a response.

Materials & experimental systems

n/a	Involved in the study	
☐	☒	Antibodies
☐	☒	Eukaryotic cell lines
☒	☐	Palaeontology
☐	☒	Animals and other organisms
☒	☐	Human research participants
☒	☐	Clinical data

Methods

n/a	Involved in the study	
☒	☐	ChIP-seq
☐	☒	Flow cytometry
☒	☐	MRI-based neuroimaging

Antibodies

Antibodies used

The antibodies used are listed in Suppl. Table 1.
Here we state in order: Antibody name; clone (Company; Catalogue number; Source; Dilution)
phospho-Ulk1 (S746) (self-produced in this paper (Eurofins), rabbit, 1:100 for immunoprecipitation, 1:400 for immunostaining)
Ulk1 (Sigma-Aldrich, A7481, rabbit, 1:2000 for immunoblotting, 1:100 for immunostaining)
GS28 Clone 1 (BD Biosciences, 611184, mouse, 1:200 for immunostaining, 1:200 for Duolink)
Lamp2 GL2A7 (Abcam, ab13524, rat, 1:200 for immunostaining)
RIPK3 (Cell Signaling Technology, 95702, rabbit, 1:1000 for immunoblotting, 1:100 for Duolink)
phospho-RIPK3 (T231/S232) (Cell Signaling Technology, 57220, rabbit, 1:1000 for immunoblotting)
PPM1D (Wip1) (Cell Signaling Technology, 11901, rabbit, 1:200 for immunoblotting)
phospho-Ulk1 (S638) (Cell Signaling Technology, 12097, rabbit, 1:500 for immunoblotting)
Atg5 (Sigma-Aldrich, A0731, rabbit, , 1:1000 for immunoblotting)
p53 PAb421 (Calbiochem, OP03-20, mouse, 1:200 for immunoblotting)
alpha-Tubulin DM1A (Sigma-Aldrich, T9026, mouse, 1:3000 for immunoblotting)
LC3 5F10 (nanotools, 5F10, mouse, 1:1000 for immunoblotting)
LC3 1703 (Cosmobio, CTB-LC3-2-IC, mouse, 1:100 for immunostaining)
p62 (MBL, PM045, rabbit, 1:1000 for immunoblotting)
HA F-7 (Santa Cruz, sc-7392, mouse, 1:1000 for immunoblotting)
phopho-MLKL (Cell Signaling Technology, 62233, rabbit, 1:1000 for immunoblotting)
VSVG 8G5F11 (KeraFAS, EB0010, mouse, 1:100 for flow cytometry)

	FLAG M2 (Sigma-Aldrich, F1804, mouse, 1:60 for immunoprecipitation, 1:100 for immunostaining)<br>DDDDK-tag (MBL, PM020, rabbit, 1:500 for immunoblotting)<br>Fip200 D10D11 (Cell Signaling Technology, 12436, rabbit, 1:1000 for immunoblotting, 1:100 for Duolink)<br>Atg13 D4P1K (Cell Signaling Technology, 13273, rabbit, 1:1000 for immunoblotting, 1:100 for Duolink)<br>Noxa 114C307.1 (Novus, NB600-1159, mouse, 1:500 for immunoblotting)<br>Integrin alpha 5 antibody 5H10-27 (Phycoerythrin) (Abcam, ab25461, rat, 1:100 for immunostaining)<br>Lamp1 (Abcam, ab24170, rabbit, 1:200 for immunostaining)
Validation	Anti-phospho-Ulk1 antibody produced in this paper were validated by ELISA and using Ulk1 KO cells. The antibodies that have been validated by the suppliers for specific purpose (for example, mouse samples using western blot and/or immunofluorescence) were purchased for our experiments. In addition, we confirmed antibodies (Ulk1 (Sigma-Aldrich, A7481), RIPK3 (Cell Signaling Technology, 95702), phospho-RIPK3 (T231/S232) (Cell Signaling Technology, 57220), PPM1D (Wip1) (Cell Signaling Technology, 11901), Atg5 (Sigma-Aldrich, A0731), phospho-Ulk1 (S638) (Cell Signaling Technology, 12097), p53 PAb421 (Calbiochem, OP03-20), and phopho-MLKL (Cell Signaling Technology, 62233))by immunoblotting using lysate from gene-specific deficient MEFs in this study. We confirmed antibodies (FLAG M2 (Sigma-Aldrich, F1804, mouse), DDDDK-tag (MBL, PM020), and HA F-7 (Santa Cruz, sc-7392) antibodies) by immunoblotting with non-transfected cell lysate and FLAG or HA-tagged proteins-transfected cell lysate. We confirmed antibodies (GS28 Clone 1 (BD Biosciences, 611184, mouse), Lamp2 GL2A7 (Abcam, ab13524), LC3 5F10 (nanotools, 5F10, mouse) by western blot and/or immunofluorescence in previous paper (Yamaguchi et al., 2016, doi: 10.15252/embj.201593191). We confirmed antibodies (alpha-Tubulin DM1A (Sigma-Aldrich, T9026), LC3 1703 (Cosmobio, CTB-LC3-2-IC), p62 (MBL, PM045, rabbit), Fip200 D10D11 (Cell Signaling Technology, 12436), Atg13 D4P1K (Cell Signaling Technology, 13273), and Noxa 114C307.1 (Novus, NB600-1159) by western blot and/or immunofluorescence in previous paper (Torii et al., 2016, doi: 10.15252/embr.201642565). We confirmed antibodies (VSVG 8G5F11 (KeraFAS, EB0010), Integrin alpha 5 antibody 5H10-27 (Phycoerythrin) (Abcam, ab25461), and Lamp1 (Abcam, ab24170) by immunofluorescence in aspect of protein subcellular localization, such as cell surface, focal adhesion and lysosome, respectively.

Eukaryotic cell lines

Policy information about cell lines

Cell line source(s)	MEFs were generated from embryos on embryonic day 14.5 by immortalization with the SV40 T antigen.
Authentication	MEFs were analyzed by PCR using specific primers to detect genome-editing.
Mycoplasma contamination	We confirm that all cell lines tested were negative for mycoplasma contamination. All cell lines are checked by staining with Hoechst and obsevation. In addition, some cells are checked by electron microscopy.
Commonly misidentified lines<br>(See ICLAC register)	No commonly misidentified cell lines were used.

Palaeontology

Specimen provenance	*Provide provenance information for specimens and describe permits that were obtained for the work (including the name of the issuing authority, the date of issue, and any identifying information).*
Specimen deposition	*Indicate where the specimens have been deposited to permit free access by other researchers.*
Dating methods	*If new dates are provided, describe how they were obtained (e.g. collection, storage, sample pretreatment and measurement), where they were obtained (i.e. lab name), the calibration program and the protocol for quality assurance OR state that no new dates are provided.*

☐ Tick this box to confirm that the raw and calibrated dates are available in the paper or in Supplementary Information.

Animals and other organisms

Policy information about studies involving animals; ARRIVE guidelines recommended for reporting animal research

Laboratory animals	Mice were bred in a 12 hr light /12 hr dark cycle at approximately 23 °C and 40% relative humidityat the Laboratory for Recombinant Animals, Tokyo Medical and Dental University, Tokyo, Japan. 5-6 weeks old WT and RIPK3 KO male mice (C57BL/6J) were used. For WT, Atg5 KO, Atg5/Ulk1 DKO, RIPK3 KO, Atg5/p53 DKO , Atg5/PPM1D DKO and Mlkl KO MEFs generation, both male and female embryos of mice (C57BL/6J) on embryonic day 14.5 were used.
Wild animals	The study did not involve wild animals.
Field-collected samples	The study did not involve samples collected from the field.
Ethics oversight	The Tokyo Medical and Dental University Ethics Committee for Animal Experiments approved all experiments in this study.

Note that full information on the approval of the study protocol must also be provided in the manuscript.

Human research participants

Policy information about studies involving human research participants

Population characteristics	*Describe the covariate-relevant population characteristics of the human research participants (e.g. age, gender, genotypic information, past and current diagnosis and treatment categories). If you filled out the behavioural & social sciences study design questions and have nothing to add here, write "See above."*
Recruitment	*Describe how participants were recruited. Outline any potential self-selection bias or other biases that may be present and how these are likely to impact results.*
Ethics oversight	*Identify the organization(s) that approved the study protocol.*

Note that full information on the approval of the study protocol must also be provided in the manuscript.

Clinical data

Policy information about clinical studies

All manuscripts should comply with the ICMJE guidelines for publication of clinical research and a completed CONSORT checklist must be included with all submissions.

Clinical trial registration	*Provide the trial registration number from ClinicalTrials.gov or an equivalent agency.*
Study protocol	*Note where the full trial protocol can be accessed OR if not available, explain why.*
Data collection	*Describe the settings and locales of data collection, noting the time periods of recruitment and data collection.*
Outcomes	*Describe how you pre-defined primary and secondary outcome measures and how you assessed these measures.*

ChIP-seq

Data deposition

☐ Confirm that both raw and final processed data have been deposited in a public database such as GEO.

☐ Confirm that you have deposited or provided access to graph files (e.g. BED files) for the called peaks.

Data access links<br>*May remain private before publication.*	*For "Initial submission" or "Revised version" documents, provide reviewer access links. For your "Final submission" document, provide a link to the deposited data.*
Files in database submission	*Provide a list of all files available in the database submission.*
Genome browser session<br>(e.g. UCSC)	*Provide a link to an anonymized genome browser session for "Initial submission" and "Revised version" documents only, to enable peer review. Write "no longer applicable" for "Final submission" documents.*

Methodology

Replicates	*Describe the experimental replicates, specifying number, type and replicate agreement.*
Sequencing depth	*Describe the sequencing depth for each experiment, providing the total number of reads, uniquely mapped reads, length of reads and whether they were paired- or single-end.*
Antibodies	*Describe the antibodies used for the ChIP-seq experiments; as applicable, provide supplier name, catalog number, clone name, and lot number.*
Peak calling parameters	*Specify the command line program and parameters used for read mapping and peak calling, including the ChIP, control and index files used.*
Data quality	*Describe the methods used to ensure data quality in full detail, including how many peaks are at FDR 5% and above 5-fold enrichment.*
Software	*Describe the software used to collect and analyze the ChIP-seq data. For custom code that has been deposited into a community repository, provide accession details.*

Flow Cytometry

Plots

Confirm that:

☒ The axis labels state the marker and fluorochrome used (e.g. CD4-FITC).

☒ The axis scales are clearly visible. Include numbers along axes only for bottom left plot of group (a 'group' is an analysis of identical markers).

☒ All plots are contour plots with outliers or pseudocolor plots.

☒ A numerical value for number of cells or percentage (with statistics) is provided.

Methodology

Sample preparation	For cell viability assay, cells were treated with reagents and stained with propidium iodide (PI) and cell viability was detected by flow cytometry. At least 10,000 events were analyzed per sample.<br>For VSVG trafficking assay, cells were transfected with VSVG–GFP, treated with or without etoposide (10 μM) in the presence of Q-VD-OPh (50 μM) at the permissive temperature (32 °C) for 60 min. Cells were then stained with an anti-VSVG antibody for 30 min at 4 °C without membrane permeabilization. After washing, the cells were stained with an Alexa fluor 633-conjugated secondary antibody for 30 min. After washing, cells were analyzed by flow cytometry. At least 10,000 events were analyzed per sample.<br>All information is as described in the methods and Figure legends section.
Instrument	BD FACS Canto II
Software	BD FACSDiva and FlowJo 10.0.7
Cell population abundance	Cells were not sorted during the experiments.
Gating strategy	For cell viability assay, cell populations were separated from cellular debris using FSC/SSC. PI-positive dead cells were detected using FSC/PI level.

☒ Tick this box to confirm that a figure exemplifying the gating strategy is provided in the Supplementary Information.

Magnetic resonance imaging

Experimental design

Design type	*Indicate task or resting state; event-related or block design.*
Design specifications	*Specify the number of blocks, trials or experimental units per session and/or subject, and specify the length of each trial or block (if trials are blocked) and interval between trials.*
Behavioral performance measures	*State number and/or type of variables recorded (e.g. correct button press, response time) and what statistics were used to establish that the subjects were performing the task as expected (e.g. mean, range, and/or standard deviation across subjects).*

Acquisition

Imaging type(s)	*Specify: functional, structural, diffusion, perfusion.*
Field strength	*Specify in Tesla*
Sequence & imaging parameters	*Specify the pulse sequence type (gradient echo, spin echo, etc.), imaging type (EPI, spiral, etc.), field of view, matrix size, slice thickness, orientation and TE/TR/flip angle.*
Area of acquisition	*State whether a whole brain scan was used OR define the area of acquisition, describing how the region was determined.*

Diffusion MRI ☐ Used ☐ Not used

Preprocessing

Preprocessing software	*Provide detail on software version and revision number and on specific parameters (model/functions, brain extraction, segmentation, smoothing kernel size, etc.).*
Normalization	*If data were normalized/standardized, describe the approach(es): specify linear or non-linear and define image types used for transformation OR indicate that data were not normalized and explain rationale for lack of normalization.*
Normalization template	*Describe the template used for normalization/transformation, specifying subject space or group standardized space (e.g. original Talairach, MNI305, ICBM152) OR indicate that the data were not normalized.*

Noise and artifact removal	*Describe your procedure(s) for artifact and structured noise removal, specifying motion parameters, tissue signals and physiological signals (heart rate, respiration).*
Volume censoring	*Define your software and/or method and criteria for volume censoring, and state the extent of such censoring.*

Statistical modeling & inference

Model type and settings	*Specify type (mass univariate, multivariate, RSA, predictive, etc.) and describe essential details of the model at the first and second levels (e.g. fixed, random or mixed effects; drift or auto-correlation).*
Effect(s) tested	*Define precise effect in terms of the task or stimulus conditions instead of psychological concepts and indicate whether ANOVA or factorial designs were used.*

Specify type of analysis: ☐ Whole brain ☐ ROI-based ☐ Both

Statistic type for inference (See Eklund et al. 2016)	*Specify voxel-wise or cluster-wise and report all relevant parameters for cluster-wise methods.*
Correction	*Describe the type of correction and how it is obtained for multiple comparisons (e.g. FWE, FDR, permutation or Monte Carlo).*

Models & analysis

n/a	Involved in the study
☐	☐ Functional and/or effective connectivity
☐	☐ Graph analysis
☐	☐ Multivariate modeling or predictive analysis

Functional and/or effective connectivity	*Report the measures of dependence used and the model details (e.g. Pearson correlation, partial correlation, mutual information).*
Graph analysis	*Report the dependent variable and connectivity measure, specifying weighted graph or binarized graph, subject- or group-level, and the global and/or node summaries used (e.g. clustering coefficient, efficiency, etc.).*
Multivariate modeling and predictive analysis	*Specify independent variables, features extraction and dimension reduction, model, training and evaluation metrics.*
