# Supplementary material for: Identification of a phosphorylation site on Ulk1 required for genotoxic stress-induced alternative autophagy
Source: Nat Commun. 2020 Apr 9;11:1754. doi: 10.1038/s41467-020-15577-2 (PMC7145817; doi:10.1038/s41467-020-15577-2)

Source data for Fig. 1

Panel B

Panel C

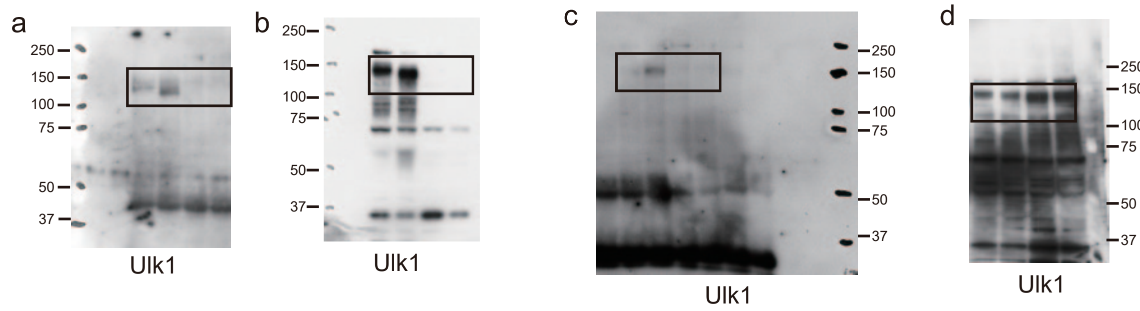

Source data for Fig. 3

Panel A

Panel B

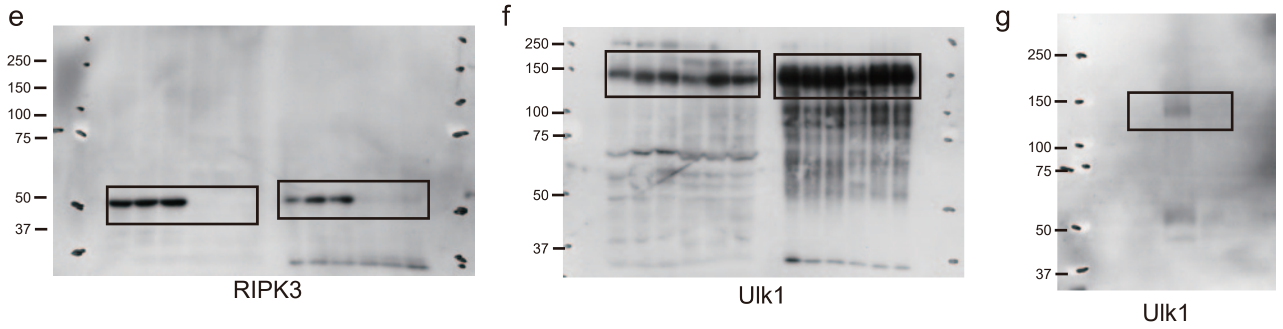

Source data for Fig. 4

Panel C

Panel F

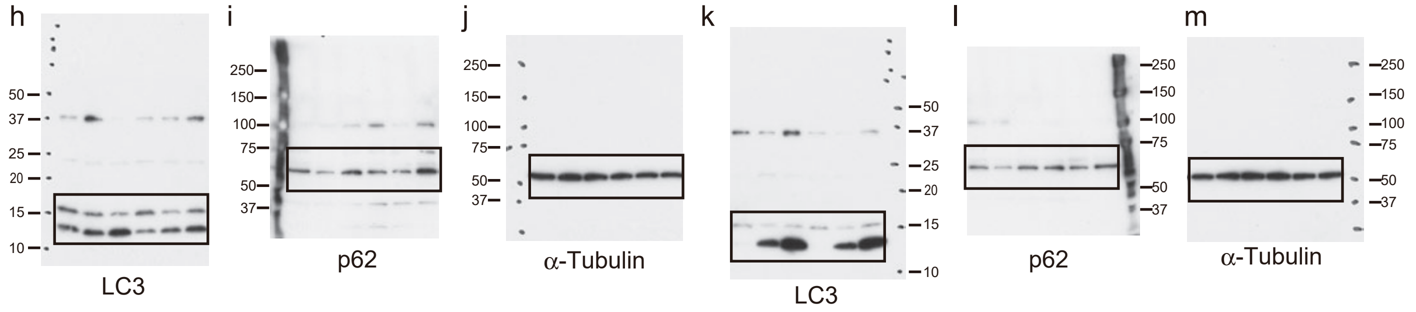

Source data for Fig. 5

Panel D

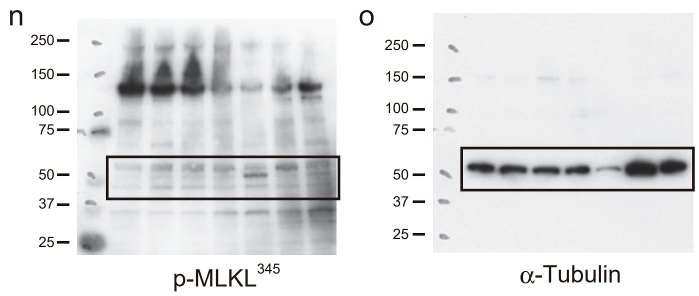

Source data for Fig. 6

Panel C

Panel I

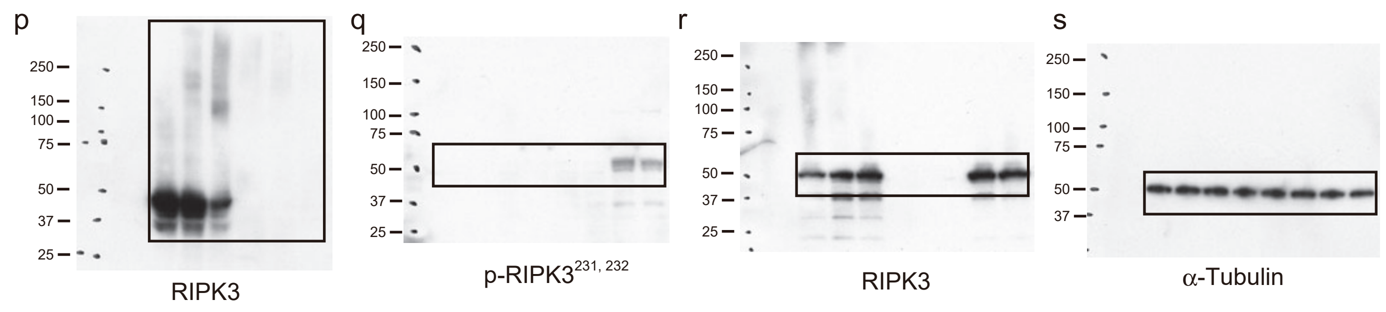

Source data for Fig. 7

Panel A   Panel E

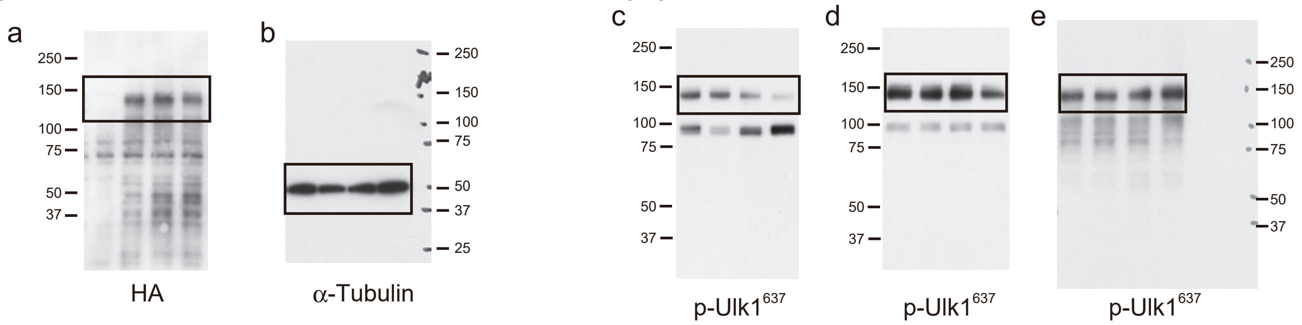

Panel E

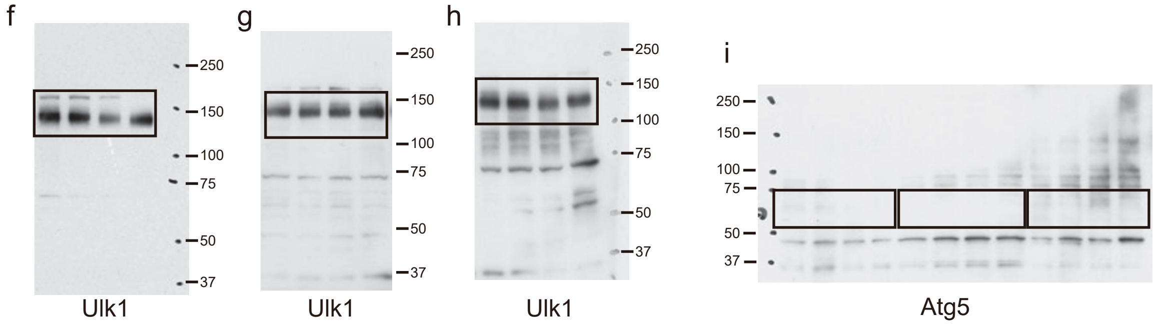

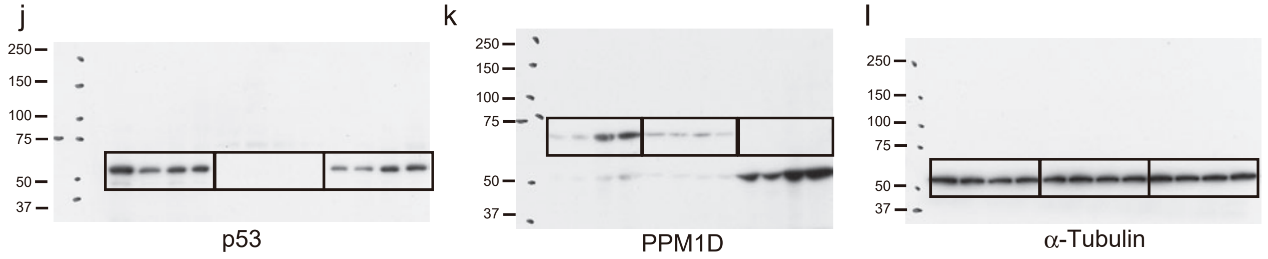

Source data for Fig. 8

Panel A

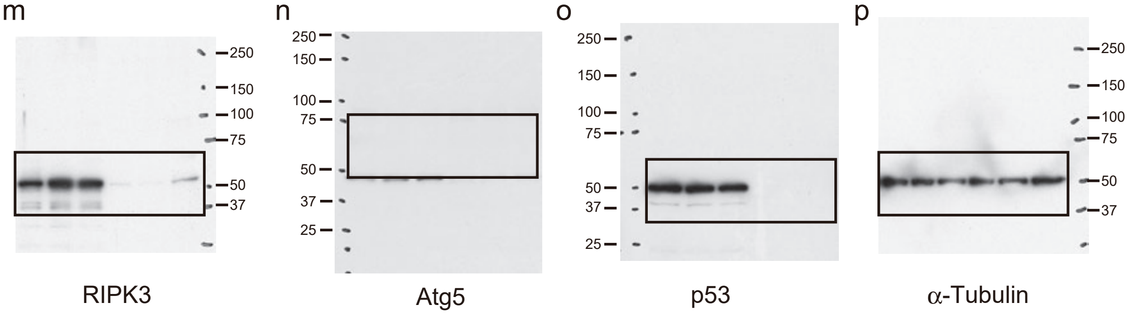

Panel H

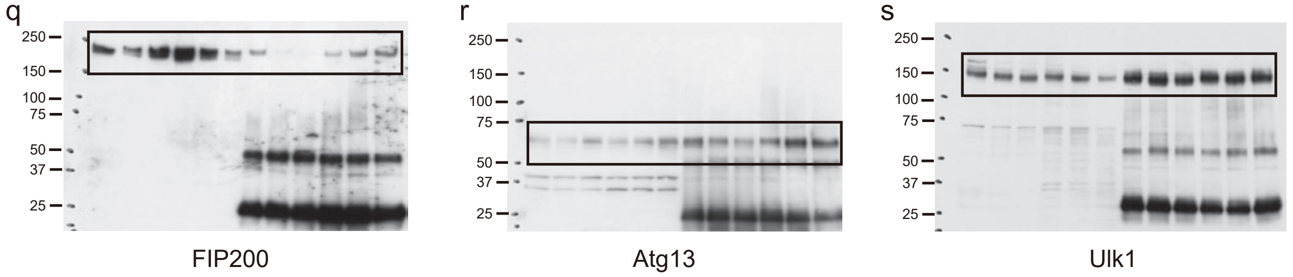

Source data for Suppl. Fig. 1

Panel A

Panel B

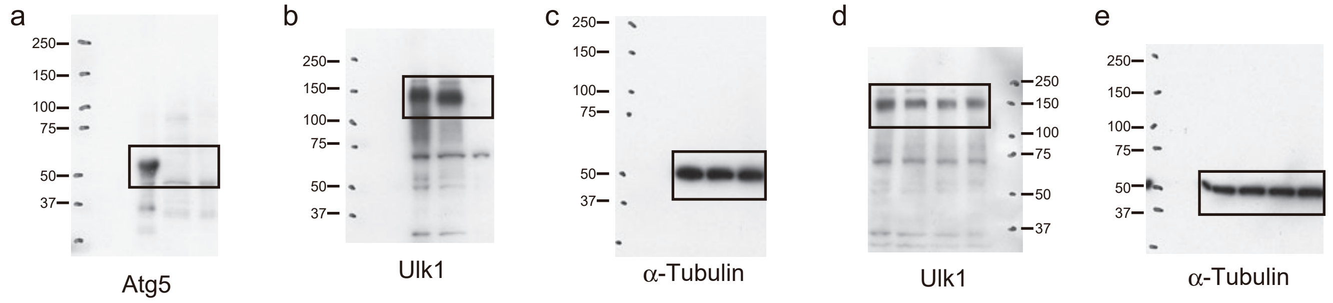

Source data for Suppl. Fig. 2

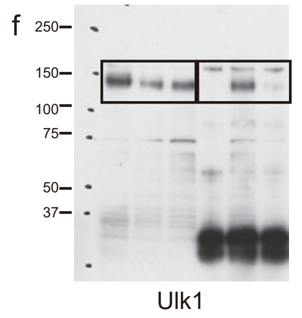

Source data for Suppl. Fig. 4

Panel B

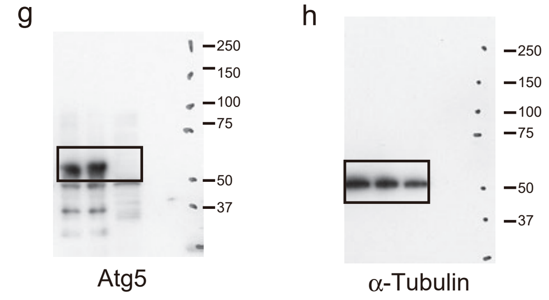

Source data for Suppl. Fig. 8

Panel A

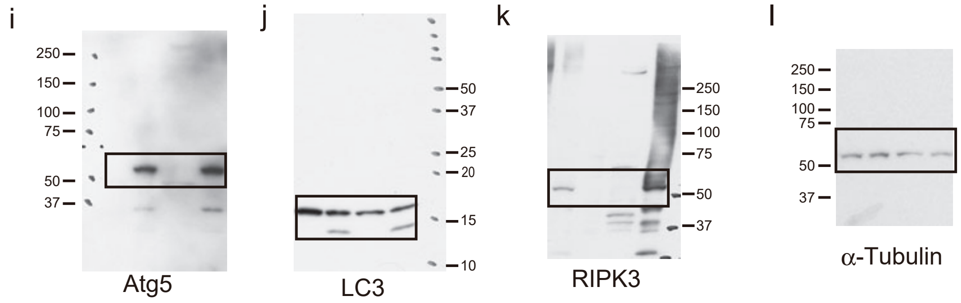

Source data for Suppl. Fig. 16

Panel B

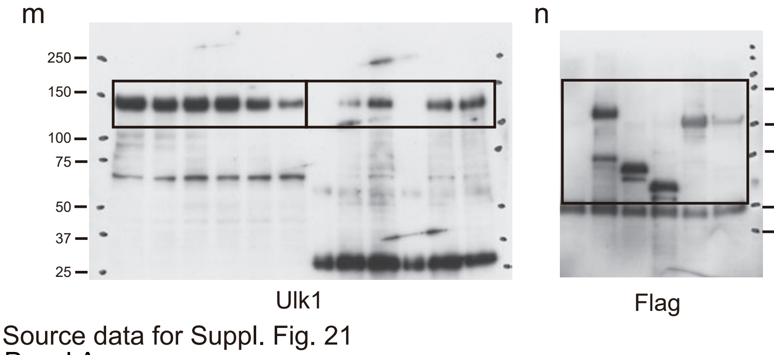

Source data for Suppl. Fig. 21

Panel A

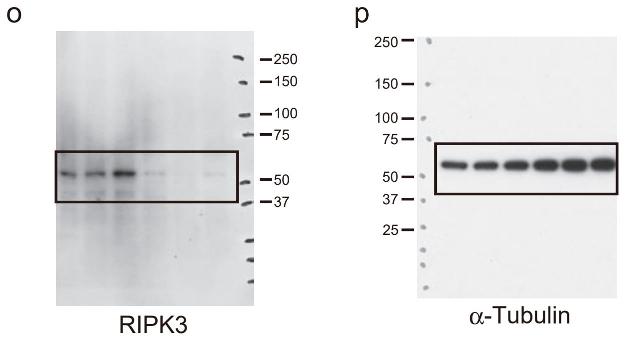

Source data for Suppl. Fig. 22

Panel A

a

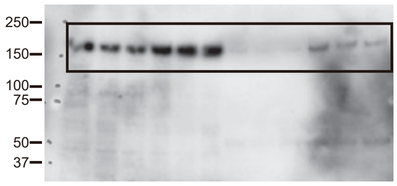

Fip200

b

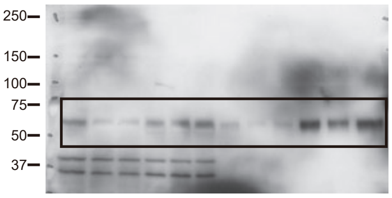

Atg13

c

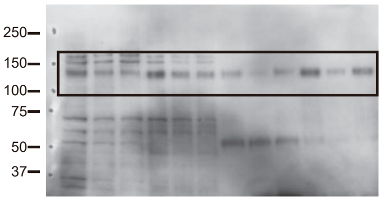

Ulk1

Source data for Suppl. Fig. 23

Panel C

d

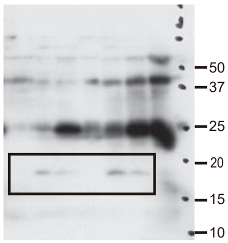

Noxa

e

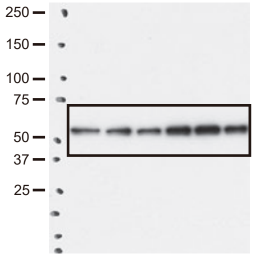

α-Tubulin

Figure 1

Panel F

	etoposide	1	2	3	Average
Atg5 KO	No treatment	0	10	13	7.7
Atg5 KO	12 hr	41	45	38	41.4
Atg5/Ulk1 DKO	No treatment	1	0	0	0.3
Atg5/Ulk1 DKO	12 hr	1	0	0	0.3
Atg5/Ulk1/2 TKO	No treatment	1	1	0	0.7
Atg5/Ulk1/2 TKO	12 hr	0	1	1	0.7
Atg5/Ulk1 DKO +WT	No treatment	0	5	2	2.3
Atg5/Ulk1 DKO +WT	12 hr	13	26	19	19.2
Atg5/Ulk1 DKO +S746A	No treatment	0	2	1	1.0
Atg5/Ulk1 DKO +S746A	12 hr	0	3	2	1.7

Figure 2

Panel F		etoposide		etoposide		etoposide		etoposide		etoposide
	A	A	AU	AU	AT	AT	AU+WT	AU+WT	AU+SA	AU+SA
1	1.2	2.3	1.2	1.2	1.0	0.9	1.1	1.8	1.2	1.0
2	1.1	1.8	1.1	1.2	1.0	0.9	1.1	1.6	1.1	1.0
3	1.1	1.8	1.1	1.2	1.0	0.9	1.1	1.4	1.0	1.0
4	1.1	1.8	1.1	1.1	0.9	0.8	1.1	1.3	1.0	0.9
5	1.1	1.4	1.0	1.1	0.9	0.8	1.1	1.3	1.0	0.9
6	1.0	1.4	1.0	1.1	0.9	0.8	1.0	1.2	1.0	0.9
7	1.0	1.4	1.0	1.0	0.9	0.8	1.0	1.2	1.0	0.9
8	1.0	1.4	1.0	1.0	0.8	0.8	1.0	1.2	1.0	0.9
9	1.0	1.4	1.0	1.0	0.8	0.8	1.0	1.2	1.0	0.9
10	0.9	1.4	1.0	1.0	0.8	0.8	1.0	1.2	1.0	0.9
11	0.9	1.4	1.0	1.0	0.8	0.8	1.0	1.2	0.9	0.9
12	0.9	1.3	1.0	1.0	0.8	0.8	1.0	1.2	0.9	0.9
13	0.9	1.3	1.0	1.0	0.8	0.8	1.0	1.2	0.9	0.9
14	0.9	1.2	1.0	0.9	0.8	0.8	1.0	1.1	0.9	0.8
15	0.9	1.2	0.9	0.9	0.8	0.8	1.0	1.1	0.9	0.8
16	0.9	1.2	0.9	0.9	0.8	0.8	0.9	1.1	0.9	0.8
17	0.9	1.2	0.9	0.9	0.7	0.7	0.9	1.1	0.9	0.8
18	0.8	1.1	0.9	0.9	0.7	0.7	0.8	1.1	0.9	0.8
19	0.8	1.1	0.9	0.9	0.7	0.7	0.8	1.1	0.8	0.8
20	0.8	1.1	0.9	0.8	0.7	0.7	0.8	1.1	0.8	0.8
Average	0.95	1.41	0.99	1.00	0.84	0.80	0.98	1.23	0.95	0.88

Panel G					
	etoposide	1	2	3	Average
Atg5 KO	No treatment	10.0	14.3	9.1	11.1
Atg5 KO	12 hr	40.7	38.3	34.1	37.7
Atg5/Ulk1 DKO	No treatment	10.1	17.2	12.8	13.4
Atg5/Ulk1 DKO	12 hr	15.4	13.5	10.5	13.1
Atg5/Ulk1/2 TKO	No treatment	11.9	12.9	9.9	11.6
Atg5/Ulk1/2 TKO	12 hr	8.9	10.9	5.9	8.6
Atg5/Ulk1 DKO+WT	No treatment	14.4	14.0	6.1	11.5
Atg5/Ulk1 DKO+WT	12 hr	30.6	34.7	36.4	33.9
Atg5/Ulk1 DKO+S746A	No treatment	5.7	13.6	6.5	8.6
Atg5/Ulk1 DKO+S746A	12 hr	2.0	9.0	9.1	6.7

Figure 3

Panel D

	etoposide	1	2	3	Average
Atg5 KO	No treatment	5	7	18	10.0
Atg5 KO	12 hr	38	35	52	41.7
AR DKO	No treatment	3	2	2	2.3
AR DKO	12 hr	4	5	3	4.0
AR DKO+R WT	No treatment	2	2	1	1.7
AR DKO+R WT	12 hr	15	25	19	19.7
AR DKO+R KD	No treatment	2	1	1	1.3
AR DKO+R KD	12 hr	3	2	2	2.3

Panel F

		etoposide		etoposide		etoposide		etoposide
	A	A	AR	AR	AR+R	AR+R	AR+R KD	AR+R KD
1	1.2	1.6	1.2	1.2	1.0	1.6	1.1	1.1
2	1.1	1.6	1.1	1.2	1.0	1.4	0.9	1.0
3	1.1	1.5	1.1	1.2	0.9	1.3	0.9	1.0
4	1.0	1.4	1.1	1.1	0.9	1.2	0.9	1.0
5	1.0	1.3	1.1	1.1	0.9	1.2	0.9	0.9
6	1.0	1.3	1.1	1.1	0.9	1.2	0.9	0.9
7	1.0	1.3	1.1	1.1	0.8	1.2	0.9	0.9
8	1.0	1.3	1.0	1.1	0.8	1.2	0.9	0.9
9	1.0	1.3	1.0	1.0	0.8	1.2	0.9	0.9
10	0.9	1.3	1.0	1.0	0.8	1.2	0.9	0.9
11	0.9	1.2	1.0	1.0	0.8	1.1	0.9	0.9
12	0.9	1.2	1.0	1.0	0.8	1.1	0.9	0.9
13	0.9	1.2	1.0	1.0	0.8	1.1	0.8	0.9
14	0.8	1.2	1.0	1.0	0.8	1.1	0.8	0.9
15	0.8	1.2	1.0	1.0	0.8	1.0	0.8	0.9
16	0.8	1.1	1.0	1.0	0.8	1.0	0.8	0.8
17	0.8	1.1	1.0	0.9	0.8	1.0	0.7	0.8
18	0.8	1.1	1.0	0.9	0.8	1.0	0.7	0.7
19	0.7	1.1	0.9	0.9	0.7	1.0	0.6	0.7
20	0.7	1.0	0.9	0.8	0.7	1.0	0.6	0.7
Average	0.9	1.3	1.0	1.0	0.8	1.2	0.8	0.9

Panel H

	etoposide		1	2	3	Average
Atg5 KO	No treatment		5.0	18.0	13.0	12.0
Atg5 KO	12 hr		33.7	52.0	31.0	38.9
Atg5 KO	No treatment	GSK'872	3.0	18.8	10.9	10.9
Atg5 KO	12 hr	GSK'872	14.9	23.8	12.9	17.2

Figure 4

Panel B

	etoposide	1	2	3	Average
Ulk1/2DKO	No treatment	5.0	3.0	1.0	3.0
Ulk1/2DKO	6 hr	10.9	12.9	5.0	9.6
Ulk1/2DKO+WT	No treatment	15.0	11.0	13.0	13.0
Ulk1/2DKO+WT	6 hr	83.0	79.0	75.0	79.0
Ulk1/2DKO+S746A	No treatment	20.0	15.0	11.0	15.3
Ulk1/2DKO+S746A	6 hr	82.0	77.0	79.0	79.3

Panel E

	etoposide	1	2	3	Average
WT	No treatment	6.0	11.0	9.0	8.7
WT	6 hr	78.0	82.0	70.0	76.7
RIP3K	No treatment	4.0	5.0	8.0	5.7
RIP3K	6 hr	66.0	75.0	68.0	69.7

Panel H

	etoposide	1	2	3	Average
Atg5KO	No treatment	10.0	13.0	16.0	13.0
Atg5KO	12 hr	45.0	38.0	57.0	46.7
WT	No treatment	6.9	11.0	13.0	10.3
WT	12 hr	35.0	22.0	42.0	33.0
RIPK3 KO	No treatment	0.0	2.0	4.0	2.0
RIPK3 KO	12 hr	0.0	4.0	9.0	4.3

Figure 5

Panel B

		1	2	3	Average
Atg5 KO	No treatment	10.0	7.0	11.0	9.3
Atg5 KO	TCZ	9.0	11.0	8.0	9.3
Atg5 KO	etoposide	20.0	15.0	28.0	21.0

Panel C

		1	2	3	Average
Atg5 KO	No treatment	11.0	5.0	6.0	7.3
Atg5 KO	TCZ 9 hr	4.0	6.0	1.0	3.7
Atg5 KO	TCZ 12 hr	11.0	10.0	14.0	11.7
Atg5 KO	etoposide	33.9	38.0	40.0	37.3

Panel E

			1	2	3	Average
WT	No treatment		2.4	0.6	0.7	1.2
WT	etoposide		32.0	38.0	42.7	37.6
WT	etoposide	Q-VD-OPh	0.7	0.9	0.7	0.8
WT	etoposide	Nec-1	39.2	44.0	38.2	40.5
MLKL KO	No treatment		1.6	0.6	3.7	2.0
MLKL KO	etoposide		43.3	43.8	45.3	44.1
MLKL KO	etoposide	Q-VD-OPh	0.9	1.0	0.9	0.9
MLKL KO	etoposide	Nec-1	42.8	46.5	49.7	46.3

Figure 6

Panel B

		1	2	3	Average
RIPK1 KO	No treatment	9.8	5.0	5.0	6.6
RIPK1 KO	etoposide	24.8	21.0	14.7	20.2

Panel E

		1	2	3	Average
AR DKO+WT	No treatment	2.0	1.0	1.0	1.3
AR DKO+WT	etoposide	17.0	21.8	13.9	17.5
AR DKO+RHIM m	No treatment	2.0	1.0	2.0	1.7
AR DKO+RHIM m	etoposide	28.0	24.8	20.8	24.5

Panel G

	AR+WT	etoposide AR+WT	AR+RHIM	etoposide AR+RHIM
1	1.0	1.6	0.9	1.7
2	1.0	1.4	0.9	1.6
3	1.0	1.3	0.9	1.5
4	1.0	1.2	0.9	1.4
5	1.0	1.2	0.9	1.4
6	1.0	1.2	0.8	1.3
7	1.0	1.2	0.8	1.2
8	1.0	1.2	0.8	1.1
9	0.9	1.2	0.8	1.1
10	0.9	1.1	0.8	1.1
11	0.9	1.1	0.8	1.1
12	0.9	1.1	0.8	1.0
13	0.9	1.1	0.8	1.0
14	0.9	1.1	0.8	1.0
15	0.8	1.0	0.8	1.0
16	0.8	1.0	0.8	1.0
17	0.8	1.0	0.8	0.9
18	0.8	1.0	0.7	0.9
19	0.8	1.0	0.7	0.9
20	0.8	0.9	0.7	0.9
Average	0.91	1.14	0.81	1.15

Figure 7

Panel C

	etoposide	1	2	3	Average
Atg5/Ulk1 DKO	No treatment	0.0	1.0	0.0	0.3
Atg5/Ulk1 DKO	12 hr	0.0	1.0	0.0	0.3
Atg5/Ulk1 DKO+WT	No treatment	0.0	5.0	2.0	2.3
Atg5/Ulk1 DKO+WT	12 hr	12.6	26.0	19.0	19.2
Atg5/Ulk1 DKO+S637D	No treatment	1.0	0.0	0.0	0.3
Atg5/Ulk1 DKO+S637D	12 hr	1.0	0.0	0.0	0.3
Atg5/Ulk1 DKO+S637A	No treatment	0.0	0.0	0.0	0.0
Atg5/Ulk1 DKO+S637A	12 hr	8.9	12.9	14.9	12.2

Panel D

	etoposide	1	2	3	Average
Atg5/Ulk1 DKO		10.1	17.2	12.8	13.4
Atg5/Ulk1 DKO	12 hr	15.4	13.5	10.5	13.1
Atg5/Ulk1 DKO+WT		14.4	14.0	6.1	11.5
Atg5/Ulk1 DKO+WT	12 hr	30.6	34.7	36.4	33.9
Atg5/Ulk1 DKO+S637D		12.7	6.9	4.9	8.2
Atg5/Ulk1 DKO+S637D	12 hr	11.8	8.8	2.9	7.8
Atg5/Ulk1 DKO+S637A		10.8	12.7	10.8	11.4
Atg5/Ulk1 DKO+S637A	12 hr	19.6	28.4	24.5	24.2

Panel G

	etoposide	1	2	3	Average
Atg5 KO	No treatment	7.7	18.0	15.0	13.6
Atg5 KO	12 hr	44.0	52.0	55.0	50.3
Atg5/p53 DKO	No treatment	3.0	10.0	3.0	5.3
Atg5/p53 DKO	12 hr	4.0	12.0	2.0	6.0
Atg5/PPM1D DKO	No treatment	1.0	7.0	0.0	2.7
Atg5/PPM1D DKO	12 hr	0.0	9.0	3.0	4.0

Panel I

	A	etoposide A	A/p53	etoposide A/p53	A/ppm1d	etoposide A/ppm1d
1	1.2	2.4	1.1	1.1	1.0	1.2
2	1.1	1.8	1.1	1.0	1.0	1.0
3	1.0	1.4	1.0	0.9	1.0	1.0
4	1.0	1.4	1.0	0.9	1.0	1.0
5	1.0	1.4	1.0	0.9	1.0	1.0
6	1.0	1.4	1.0	0.9	0.9	1.0
7	0.9	1.3	1.0	0.9	0.9	1.0
8	0.9	1.3	1.0	0.9	0.9	0.9
9	0.9	1.3	0.9	0.9	0.9	0.9
10	0.9	1.2	0.9	0.9	0.9	0.8
11	0.9	1.2	0.9	0.9	0.9	0.8
12	0.9	1.2	0.9	0.9	0.9	0.8
13	0.9	1.2	0.9	0.9	0.9	0.8
14	0.9	1.1	0.9	0.8	0.9	0.8
15	0.8	1.1	0.9	0.8	0.9	0.8
16	0.8	1.1	0.9	0.8	0.9	0.8
17	0.8	1.1	0.9	0.8	0.9	0.8
18	0.8	1.1	0.8	0.8	0.8	0.8
19	0.8	1.0	0.8	0.8	0.8	0.8
20	0.8	0.9	0.7	0.8	0.7	0.7
Average	0.9	1.3	0.9	0.9	0.9	0.9

Figure 8

Panel B

	etoposide	1	2	3	Average
Atg5 KO	No	1.0	1.0	1.0	1.0
Atg5 KO	8 hr	6.4	3.3	7.5	5.7
Atg5 KO	10 hr	1.9	0.2	0.4	0.8
Atg5/p53DKO	No	1.1	1.6	0.1	0.9
Atg5/p53DKO	8 hr	0.3	2.2	0.1	0.8
Atg5/p53DKO	10 hr	0.3	1.1	0.0	0.5

**Panel D**	Atg5 KO	Atg5 KO+e
1	26	74
2	24	54
3	18	49
4	18	43
5	17	39
6	15	37
7	15	34
8	15	31
9	15	30
10	14	29
11	13	26
12	13	24
13	12	24
14	12	24
15	12	24
16	11	24
17	11	24
18	11	22
19	11	22
20	11	21
21	11	21
22	11	20
23	11	19
24	10	18
25	10	18
26	10	18
27	10	18
28	10	17
29	10	17
30	10	17
31	9	16
32	9	15
33	9	15
34	9	14
35	8	14
36	8	13
37	8	13
38	8	13
39	8	12
40	7	12
41	7	12
42	6	12
43	6	11
44	6	11
45	6	9
46	5	9
47	4	8
48	4	8
49	3	7
50	2	6
Average	10.58	21.36

**Panel F**	AU+WT	AU+WT+e	AU+SA	AU+SA+e
1	4	10	4	7
2	3	9	1	6
3	3	8	0	5
4	3	7	0	5
5	3	7	0	5
6	3	7	0	4
7	2	6	0	4
8	2	6	0	4
9	2	6	0	4
10	2	6	0	3
11	2	6	0	3
12	2	6	0	3
13	2	5	0	2
14	2	5	0	2
15	1	5	0	2
16	1	5	0	2
17	1	5	0	2
18	1	5	0	2
19	1	5	0	2
20	1	5	0	2
21	1	5	0	1
22	1	5	0	1
23	1	5	0	1
24	1	5	0	1
25	1	4	0	1
26	1	4	0	1
27	1	4	0	1
28	1	4	0	1
29	1	4	0	1
30	1	4	0	1
31	0	4	0	1
32	0	4	0	1
33	0	3	0	1
34	0	3	0	0
35	0	3	0	0
36	0	3	0	0
37	0	3	0	0
38	0	3	0	0
39	0	2	0	0
40	0	2	0	0
41	0	2	0	0
42	0	2	0	0
43	0	2	0	0
44	0	2	0	0
45	0	2	0	0
46	0	2	0	0
47	0	1	0	0
48	0	1	0	0
49	0	1	0	0
50	0	1	0	0
Average	1.02	4.28	0.1	1.64

Figure 9

Panel E

	etoposide Atg5 KO	etoposide E64d+pep Atg5 KO	etoposide AR DKO	etoposide E64d+pep AR DKO
1	1.8	3.2	3.9	3.7
2	1.7	3.1	3.9	3.2
3	1.6	2.9	3.6	2.9
4	1.5	2.6	3.5	2.6
5	1.4	2.6	3.1	2.6
6	1.4	2.4	3.1	2.4
7	1.3	2.0	3.1	2.3
8	1.3	2.0	2.9	2.3
9	1.3	1.9	2.9	2.0
10	1.2	1.9	2.2	1.9
11	1.2	1.9	2.2	1.8
12	1.1	1.8	2.0	1.8
13	1.1	1.6	2.0	1.6
14	1.0	1.6	1.9	1.5
15	1.0	1.5	1.9	1.5
16	0.9	1.4	1.6	1.4
17	0.9	1.4	1.4	1.4
18	0.9	1.4	1.4	1.4
19	0.9	1.4	1.4	1.3
20	0.8	1.4	1.3	1.3
21	0.8	1.3	1.1	1.3
22	0.7	1.2	1.0	1.3
23	0.7	1.1	0.9	1.2
24	0.7	1.1	0.9	1.0
25	0.6	1.0	0.9	1.0
26	0.5	1.0	0.8	1.0
27	0.5	1.0	0.8	0.9
28	0.4	0.9	0.8	0.9
29	0.3	0.9	0.7	0.9
30	0.2	0.6	0.6	0.5
Average	1.0	1.7	1.9	1.7

Figure 10

Panel B

	etoposide Atg5 KO	etoposide E64d+pep Atg5 KO	etoposide AR DKO	etoposide E64d+pep AR DKO
1	2.8	3.4	2.2	2.5
2	1.6	2.9	2.1	2.5
3	1.6	2.7	2.1	2.3
4	1.6	2.5	2.1	2.1
5	1.5	2.4	2.1	2.1
6	1.5	2.4	2.0	2.0
7	1.5	2.3	1.9	2.0
8	1.2	2.3	1.8	1.9
9	1.0	2.2	1.6	1.9
10	1.0	2.2	1.6	1.9
11	1.0	2.1	1.6	1.7
12	1.0	2.0	1.5	1.6
13	1.0	1.8	1.5	1.6
14	0.8	1.8	1.5	1.5
15	0.8	1.7	1.4	1.5
16	0.8	1.6	1.4	1.5
17	0.8	1.6	1.4	1.4
18	0.8	1.5	1.4	1.4
19	0.7	1.5	1.4	1.3
20	0.7	1.4	1.3	1.3
21	0.7	1.4	1.2	1.3
22	0.7	1.4	1.1	1.3
23	0.7	1.3	1.1	1.2
24	0.7	1.3	0.9	1.1
25	0.7	1.3	0.9	1.1
26	0.6	1.3	0.9	1.1
27	0.6	1.1	0.8	1.1
28	0.6	1.1	0.8	0.9
29	0.6	1.1	0.7	0.9
30	0.5	1.0	0.5	0.8
Average	1.0	1.8	1.4	1.6

Supplementary Figure 1

Panel D

	etoposide	1
Atg5/Ulk1 DKO+WT	No	14.4
Atg5/Ulk1 DKO+WT	12 hr	30.6
Atg5/Ulk1 DKO+S317A	No	21.2
Atg5/Ulk1 DKO+S317A	12 hr	22.3
Atg5/Ulk1 DKO+S494A	No	10.9
Atg5/Ulk1 DKO+S494A	12 hr	29.5
Atg5/Ulk1 DKO+S746A	No	5.7
Atg5/Ulk1 DKO+S746A	12 hr	2.0

Supplementary Figure 3

Panel A

	etoposide	1	2	3	Average
Atg5 KO		11.0	15.0	5.0	10.3
Atg5 KO	9 hr	20.0	15.0	28.0	21.0
Atg5 KO	12 hr	35.0	52.0	45.0	44.0

Panel B

	etoposide	1	2	3	Average
Atg5 KO		10.9	7.9	6.9	9
Atg5 KO	10 uM	31.7	42.6	40.6	38
Atg5 KO	20 uM	45.0	54.5	52.5	51

Supplementary Figure 7

Panel B

	etoposide		1.0	2.0	3.0	Average
Atg5 KO	No treatment		4.0	11.0	5.0	6.7
Atg5 KO	12 hr		35.0	44.0	36.0	38.3
Atg5 KO	No treatment	Q-VD	4.0	1.0	6.0	3.7
Atg5 KO	12 hr	Q-VD	38.0	41.0	25.0	34.7

Supplementary Figure 8

Panel D

	etoposide	1	2	3	Average
Atg5 KO		10.6	8.0	5.0	7.9
Atg5 KO	12 hr	33.9	38.0	40.0	37.3
AR DKO		4.8	6.9	3.0	4.9
AR DKO	12 hr	7.8	4.9	5.0	5.9
AR DKO+WT		6.0	3.0	5.0	4.7
AR DKO+WT	12 hr	23.0	32.0	25.0	26.7
AR DKO+KD		4.0	7.0	5.0	5.3
AR DKO+KD	12 hr	11.0	10.0	7.0	9.3

Panel F

		etoposide		etoposide
			GSK' 872	GSK' 872
	Atg5 KO	Atg5 KO	Atg5 KO	Atg5 KO
1	1.1	1.8	1.2	1.1
2	1.1	1.8	1.1	1.1
3	1.1	1.4	1.1	1.0
4	1.0	1.4	1.1	1.0
5	1.0	1.4	1.1	1.0
6	1.0	1.4	1.1	1.0
7	1.0	1.4	1.0	1.0
8	1.0	1.4	1.0	0.9
9	1.0	1.4	1.0	0.9
10	0.9	1.3	1.0	0.9
11	0.9	1.3	1.0	0.9
12	0.9	1.2	1.0	0.9
13	0.9	1.2	1.0	0.9
14	0.9	1.2	1.0	0.9
15	0.9	1.2	1.0	0.9
16	0.9	1.1	1.0	0.9
17	0.9	1.1	0.9	0.8
18	0.9	1.1	0.9	0.8
19	0.8	1.1	0.9	0.8
20	0.8	1.1	0.9	0.8
Average	0.9	1.3	1.0	0.9

Supplementary Figure 9

Panel B

	Camptotheci	1	2	3	Average
Atg5 KO		13.9	10.9	5.0	9.9
Atg5 KO	12 hr	34.7	30.7	28.7	31.4
Atg5/RIPK3 DKO		5.0	5.0	7.9	5.9
Atg5/RIPK3 DKO	12 hr	9.9	10.9	4.0	8.3

Panel C

	UVC	1	2	3	Average
Atg5 KO		13.9	10.9	5.0	9.9
Atg5 KO	1hr	19.8	14.9	14.9	16.5
Atg5 KO	3hr	40.6	32.7	30.7	34.7
Atg5/RIPK3 DKO		5.0	5.0	7.9	5.9
Atg5/RIPK3 DKO	1hr	5.0	2.0	5.0	4.0
Atg5/RIPK3 DKO	3hr	11.9	5.0	5.0	7.3

Supplementary Figure 10

Panel B

	Ulk12DKO		Ulk1/2DKO+WT		Ulk1/2DKO+S746A	
		etoposide		etoposide		etoposide
1	5	0	0	50	1	26
2	6	2	0	7	2	24
3	3	2	0	20	0	5
4	7	0	4	49	1	31
5	2	0	1	23	7	32
6	0	1	2	10	0	39
7	0	0	0	22	4	7
8	0	0	1	8	0	33
9	0	0	2	11	0	19
10	0	1	2	25	0	12
Average	2.3	0.6	1.2	22.5	1.5	22.8

Panel D

	WT		RIPK3 KO	
		etoposide		etoposide
1	2	38	4	1
2	3	12	3	11
3	1	15	0	28
4	0	13	0	23
5	0	10	1	15
6	0	46	1	23
7	1	12	0	22
8	0	28	4	18
9	0	18	4	28
10	0	7	0	10
Average	0.7	19.9	1.7	17.9

Supplementary Figure 11

Panel B

		1	2	3	Average
WT	No treatment	7.0	2.0	4.0	4.3
WT	Rapamycin	5.0	4.0	5.9	5.0
WT	etoposide	23.0	27.7	24.3	25.0

Panel D

	Starvation	1	2	3	Average
Atg5 KO	No treatment	10.9	10.9	14.9	12.2
Atg5 KO	2 hr	11.9	13.9	13.9	13.2
Atg5 KO	4 hr	9.9	14.9	11.9	12.2

Supplementary Figure 12

Panel B

	Ulk1/2 DKO		Ulk1/2 DKO+WT		Ulk1/2 DKO+S746A	
		starvation		starvation		starvation
1	0	6	0	6	3	49
2	3	0	0	6	1	29
3	0	2	1	3	2	13
4	6	2	1	17	2	12
5	2	0	0	6	3	19
6	2	2	0	16	3	9
7	0	2	1	5	3	12
8	2	2	0	21	0	20
9	0	3	1	35	0	13
10	0	1	0	12	0	14
Average	1.5	2.0	0.4	12.7	1.7	19.0

Panel D

	WT		RIPK3 KO	
		starvation		starvation
1	0	28	3	27
2	0	16	2	17
3	0	10	0	22
4	0	15	0	8
5	1	14	1	6
6	0	17	0	5
7	1	15	4	17
8	0	26	0	9
9	0	21	0	3
10	1	13	0	11
Average	0.3	17.5	1	12.5

Supplementary Figure 13

Panel B

		1	2	3	Average
WT thymocyte	no treatment	2.0	1.0	0.0	1.0
WT thymocyte	etoposide	22.0	26.0	29.0	25.7
RIPK3 KO thymocyte	no treatment	1.0	2.0	1.0	1.3
RIPK3 KO thymocyte	etoposide	2.0	2.0	3.0	2.3

Supplementary Figure 15

Panel B

	etoposide	1	2	3	Average
WT	no treatment	11.9	7.0	9.8	9.6
WT	12 hr	28.7	23.0	27.7	26.5
RIPK3 KO	no treatment	0.0	2.0	4.0	2.0
RIPK3 KO	12 hr	0.0	4.0	9.0	4.3
MLKL KO	no treatment	9.8	9.8	5.0	8.2
MLKL KO	12 hr	20.8	16.8	14.7	17.4

Supplementary Figure 18

Panel B

	etoposide	1	2	3	Average
Atg5 KO	no treatment	10.9	8.0	5.0	8.0
Atg5 KO	12 hr	32.5	38.0	40.0	36.8
Atg5/p53 DKO	no treatment	4.8	7.0	10.0	7.3
Atg5/p53 DKO	12 hr	4.8	6.0	7.0	5.9
Atg5/PPM1D DKO	no treatment	5.0	14.5	10.9	10.1
Atg5/PPM1D DKO	12 hr	5.0	20.0	5.5	10.2

Supplementary Figure 19

Panel B

	etoposide	1	2	3	Average
Atg5/PPM1D DKO	no treatment	1.0	7.0	0.0	2.7
Atg5/PPM1D DKO	12 hr	0.0	9.0	3.0	4.0
Atg5/PPM1D DKO+S637A	no treatment	0.0	2.0	1.0	1.0
Atg5/PPM1D DKO+S637A	12 hr	10.9	19.8	21.8	17.5

Panel D

	etoposide	1	2	3	Average
Atg5/PPM1D DKO	no treatment	5.0	14.5	10.9	10.1
Atg5/PPM1D DKO	12 hr	5.0	20.0	5.5	10.2
Atg5/PPM1D DKO+S637A	no treatment	9.8	8.8	14.7	11.1
Atg5/PPM1D DKO+S637A	12 hr	24.5	29.4	22.5	25.5

Supplementary Figure 20

Panel B

	etoposide	1	2	3	Average
Atg5 KO	no treatment	14.3	10.9	10.6	11.9
Atg5/Ulk1 DKO+S746D	no treatment	9.9	11.0	3.0	8.0
Atg5/Ulk1 DKO+S637A S746D	no treatment	26.8	22.7	24.3	24.6

Supplementary Figure 21

Panel C

		1	2	3	Average
Atg5/Ulk1 DKO+S637A		1.0	3.0	3.0	2.3
Atg5/Ulk1 DKO+S637A	Flag-RIPK3 (WT)	26.7	17.8	12.9	19.1
Atg5/Ulk1 DKO+S637A	Flag-RIPK3 (K51A)	5.9	5.0	2.0	4.3

Supplementary Figure 22

Panel C

	Atg5KO	etoposide Atg5KO	Atg5KO	etoposide Atg5KO
	Fip200, GS28		Atg13, GS28	
1	7	3	2	1
2	6	2	1	1
3	6	2	1	1
4	6	2	1	1
5	5	1	1	1
6	5	1	1	1
7	4	1	0	1
8	4	1	0	1
9	4	1	0	1
10	4	1	0	0
11	4	1	0	0
12	4	1	0	0
13	4	1	0	0
14	3	1	0	0
15	3	1	0	0
16	3	1	0	0
17	3	1	0	0
18	3	1	0	0
19	3	1	0	0
20	3	1	0	0
21	3	1	0	0
22	3	1	0	0
23	3	1	0	0
24	3	1	0	0
25	2	1	0	0
26	2	0	0	0
27	2	0	0	0
28	2	0	0	0
29	2	0	0	0
30	2	0	0	0
31	2	0	0	0
32	2	0	0	0
33	2	0	0	0
34	2	0	0	0
35	2	0	0	0
36	1	0	0	0
37	1	0	0	0
38	1	0	0	0
39	1	0	0	0
40	1	0	0	0
41	1	0	0	0
42	1	0	0	0
43	1	0	0	0
44	1	0	0	0
45	0	0	0	0
46	0	0	0	0
47	0	0	0	0
48	0	0	0	0
49	0	0	0	0
50	0	0	0	0
Average	2.54	0.6	0.14	0.18

Supplementary Figure 23

Panel A

Thymocytes	WT					
	etopside	0 hr	10 hr	25 hr	35 hr	55 hr
	1	14.2	18.1	40.1	56.9	57.1
	2	8.2	9.6	36.1	43.3	44.8
	3	6.5	11.7	45.1	46.4	61.4
	Average	9.6	13.1	40.4	48.9	54.4
Thymocytes	RIPK3 KO					
	etopside	0 hr	10 hr	25 hr	35 hr	55 hr
	1	14.5	16.1	30	50.2	52.6
	2	9.7	13.9	30.3	40	39.2
	3	8.6	10.6	41.9	47.4	59.5
	Average	10.9	13.5	34.1	45.9	50.4

Panel B

Thymocytes	WT					
	Camptothesin	0 hr	10 hr	25 hr	35 hr	55 hr
	1	14.2	16.4	22.9	47.4	54.6
	2	8.2	8.6	19.4	39.3	45.5
	3	6.5	12.5	20.1	33.6	55.2
	Average	9.6	12.5	20.8	40.1	51.8
Thymocytes	RIPK3 KO					
	Camptothesin	0 hr	10 hr	25 hr	35 hr	55 hr
	1	14.5	17	23	39.2	55.5
	2	9.7	10	17.6	31	45.6
	3	8.6	8.9	18.9	30.2	59
	Average	10.9	12.0	19.8	33.5	53.4

Panel D

Splenocyte	WT					
	etopside	0 hr	10 hr	25 hr	35 hr	55 hr
	1	19.7	22.2	16.8	33.1	64.1
	2	18.8	19.6	20.3	32.3	51.1
	3	17.5	18.4	20.8	38.8	62.4
	Average	18.7	20.1	19.3	34.7	59.2
Splenocyte	RIPK3 KO					
	etopside	0 hr	10 hr	25 hr	35 hr	55 hr
	1	21.5	27.6	24.2	34.9	64.7
	2	22	21.6	18.7	35.8	52.7
	3	11.3	13.9	23.3	45.7	53
	Average	18.3	21.0	22.1	38.8	56.8

Panel E

Splenocyte	WT					
	Camptothesin	0 hr	10 hr	25 hr	35 hr	55 hr
	1	19.7	20.3	19.5	31.7	52.4
	2	18.8	18.5	19.6	27.3	42.4
	3	17.5	13.4	27.2	36.7	54.9
	Average	18.7	17.4	22.1	31.9	49.9
Splenocyte	RIPK3 KO					
	Camptothesin	0 hr	10 hr	25 hr	35 hr	55 hr
	1	21.5	23.7	26	36.8	54.6
	2	22	20.2	20.9	32.2	47.2
	3	11.3	14	22.3	38.4	63.7
	Average	18.3	19.3	23.1	35.8	55.2

Supplementary Figure 24

Panel B

	Camptothecin<br>Atg5 KO	Camptothecin E64d+pep<br>Atg5 KO	Camptothecin<br>AR DKO	Camptothecin E64d+pep<br>AR DKO
1	2.4	4.0	3.8	4.0
2	2.3	3.9	3.7	3.8
3	2.1	3.8	3.7	3.4
4	1.9	3.4	3.6	3.0
5	1.8	2.8	3.2	2.8
6	1.3	2.6	3.2	2.4
7	1.2	2.3	2.9	2.2
8	1.2	2.2	2.8	2.1
9	1.2	2.1	2.8	1.9
10	1.2	2.1	2.5	1.9
11	1.1	1.8	2.4	1.9
12	1.0	1.3	2.4	1.8
13	0.9	1.3	2.3	1.8
14	0.9	1.3	2.2	1.2
15	0.9	1.3	2.1	1.2
16	0.8	1.1	2.1	1.1
17	0.8	1.1	1.9	1.0
18	0.8	1.0	1.7	1.0
19	0.7	1.0	1.7	0.9
20	0.7	1.0	1.6	0.9
21	0.6	1.0	1.4	0.9
22	0.6	1.0	1.3	0.9
23	0.6	0.9	1.3	0.8
24	0.6	0.9	1.0	0.8
25	0.5	0.9	0.9	0.8
26	0.5	0.8	0.8	0.8
27	0.4	0.8	0.7	0.7
28	0.4	0.8	0.6	0.7
29	0.3	0.8	0.5	0.7
30	0.2	0.7	0.4	0.6
Average	1.0	1.7	2.1	1.6

Supplementary Figure 25

Panel B

	etoposide AU DKO+WT	etoposide E64d+pep AU DKO+WT	etoposide AU DKO+SA	etoposide E64d+pep AU DKO+SA
1	2.7	3.9	4.0	3.9
2	2.4	3.3	3.6	3.1
3	1.7	2.6	3.4	3.0
4	1.6	2.5	3.4	2.8
5	1.5	2.5	3.2	2.5
6	1.3	2.5	2.9	2.5
7	1.3	2.4	2.8	2.4
8	1.3	2.3	2.6	2.1
9	1.2	2.3	2.5	1.9
10	1.2	2.2	2.5	1.8
11	1.1	1.9	2.4	1.8
12	1.1	1.8	2.1	1.7
13	1.0	1.6	2.0	1.7
14	1.0	1.5	2.0	1.7
15	1.0	1.4	1.8	1.7
16	0.9	1.3	1.6	1.5
17	0.9	1.3	1.5	1.5
18	0.9	1.1	1.5	1.5
19	0.8	1.0	1.3	1.4
20	0.6	1.0	1.3	1.2
21	0.6	1.0	1.2	1.2
22	0.6	0.9	1.2	1.2
23	0.6	0.9	1.1	1.1
24	0.5	0.8	1.1	1.1
25	0.5	0.8	1.0	1.0
26	0.4	0.8	0.9	0.9
27	0.4	0.8	0.8	0.8
28	0.4	0.8	0.7	0.8
29	0.4	0.6	0.7	0.6
30	0.4	0.5	0.6	0.5
Average	1.0	1.6	1.9	1.7

Supplementary Figure 26

Panel D

	etoposide	etoposide E64d+pep	etoposide	etoposide E64d+pep	etoposide	etoposide E64d+pep
	AUDKO	AU DKO	AU DKO+WT	AU DKO+WT	AU DKO+S/	AU DKO+SA
1	3.0	3.6	2.4	3.8	3.8	3.2
2	2.9	2.9	2.0	3.7	2.1	2.2
3	2.6	2.8	1.9	3.4	2.1	2.0
4	2.5	2.7	1.9	3.3	2.0	1.8
5	2.4	2.6	1.8	3.2	1.9	1.8
6	2.4	2.6	1.7	3.0	1.9	1.8
7	2.4	2.5	1.7	2.7	1.8	1.7
8	2.3	2.4	1.7	2.4	1.7	1.7
9	2.0	2.3	1.7	2.1	1.7	1.5
10	2.0	2.0	1.6	2.1	1.7	1.4
11	1.9	2.0	1.5	1.9	1.7	1.4
12	1.7	1.9	1.4	1.8	1.7	1.3
13	1.7	1.9	1.4	1.7	1.6	1.3
14	1.6	1.9	1.2	1.6	1.6	1.3
15	1.6	1.9	1.0	1.6	1.6	1.2
16	1.5	1.8	0.8	1.6	1.6	1.2
17	1.5	1.7	0.6	1.6	1.5	1.2
18	1.5	1.7	0.4	1.6	1.5	1.1
19	1.3	1.4	0.4	1.5	1.5	1.1
20	1.3	1.4	0.3	1.5	1.4	1.1
21	1.3	1.4	0.3	1.5	1.3	1.1
22	1.1	1.4	0.3	1.5	1.3	1.1
23	1.1	1.3	0.3	1.4	1.3	1.1
24	1.1	1.3	0.3	1.3	1.3	1.1
25	1.0	1.2	0.3	1.3	1.3	1.1
26	0.9	1.0	0.3	1.2	1.2	1.0
27	0.9	0.9	0.2	1.1	1.1	1.0
28	0.7	0.9	0.2	1.1	1.0	0.9
29	0.7	0.8	0.2	0.8	0.9	0.8
30	0.7	0.8	0.2	0.7	0.8	0.8
Average	1.7	1.8	1.0	1.9	1.6	1.4

Torii_Source Image 1

Source data for Fig. 1

Panel D

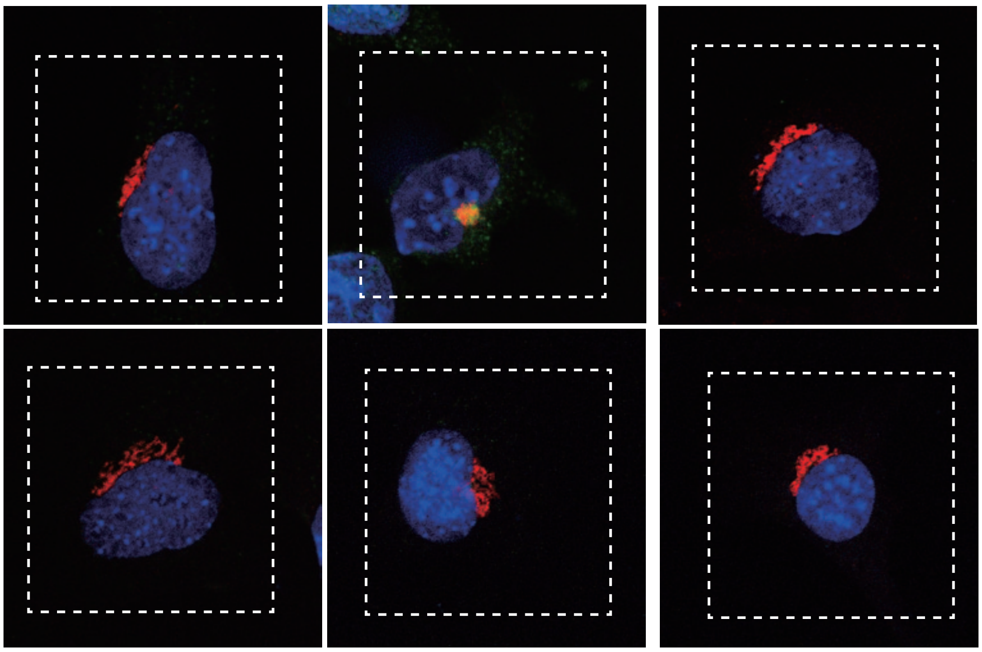

Panel E

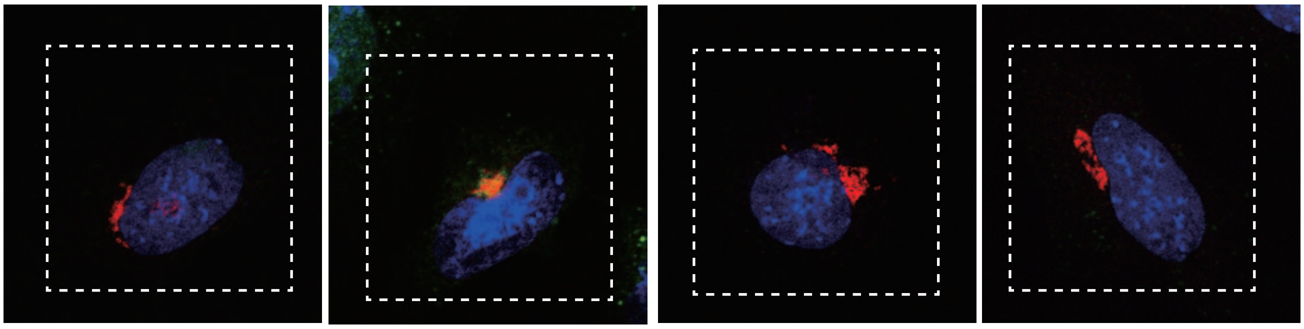

Source data for Fig. 2

Panel B

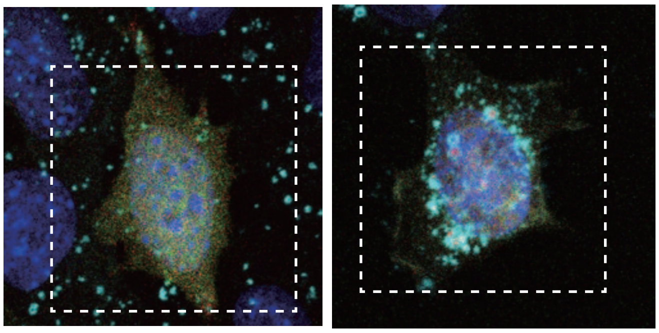

Panel C

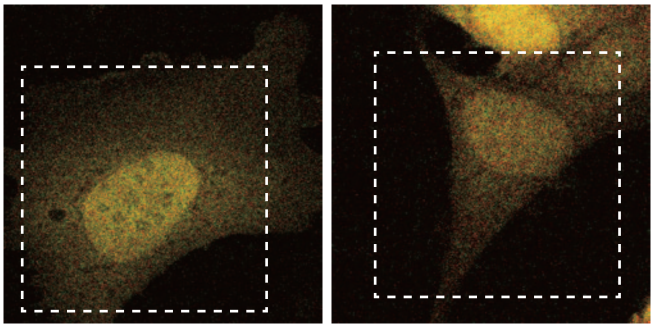

Panel D

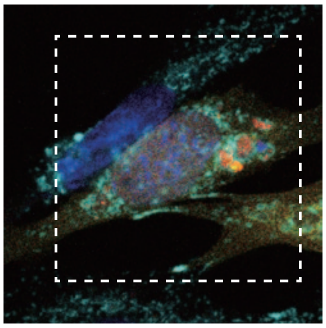

Panel E

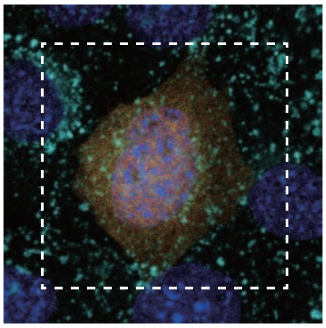

Source data for Fig. 3

Panel C

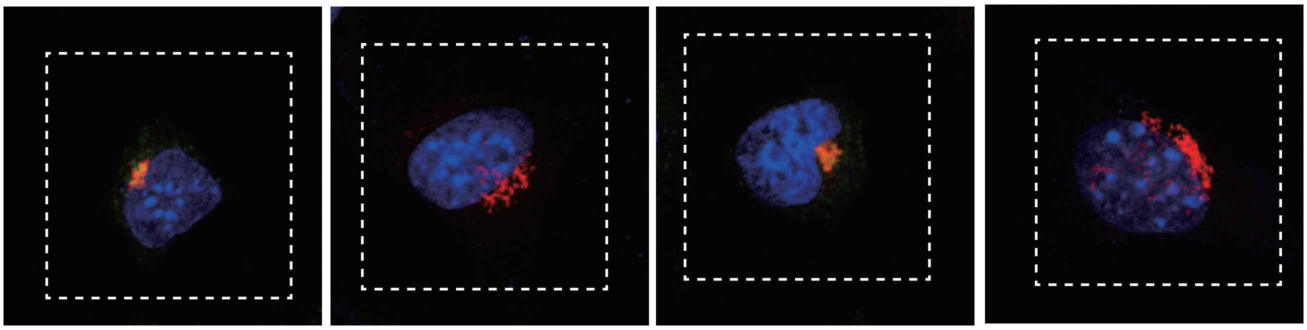

Panel E

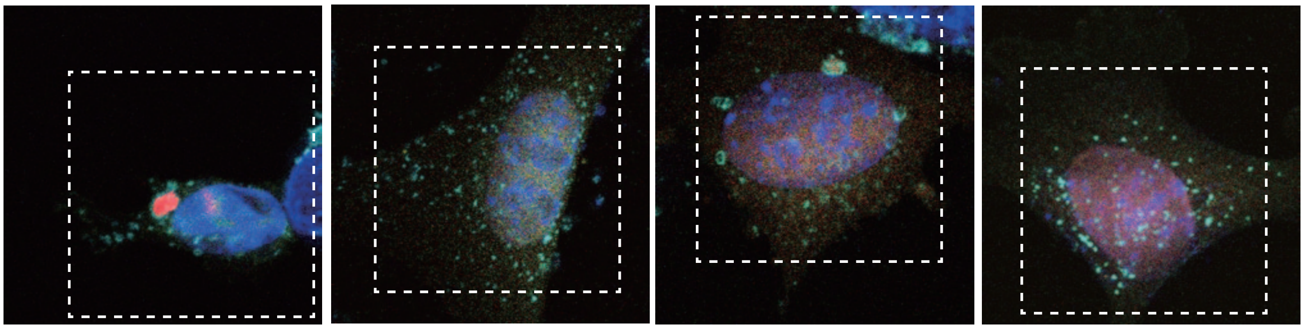

Panel G

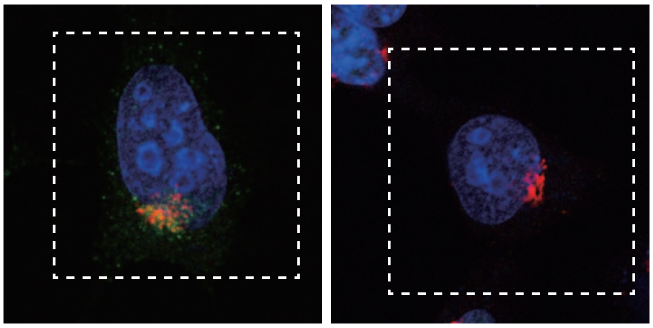

Torii_Source Image 3

Source data for Fig. 4

Panel A

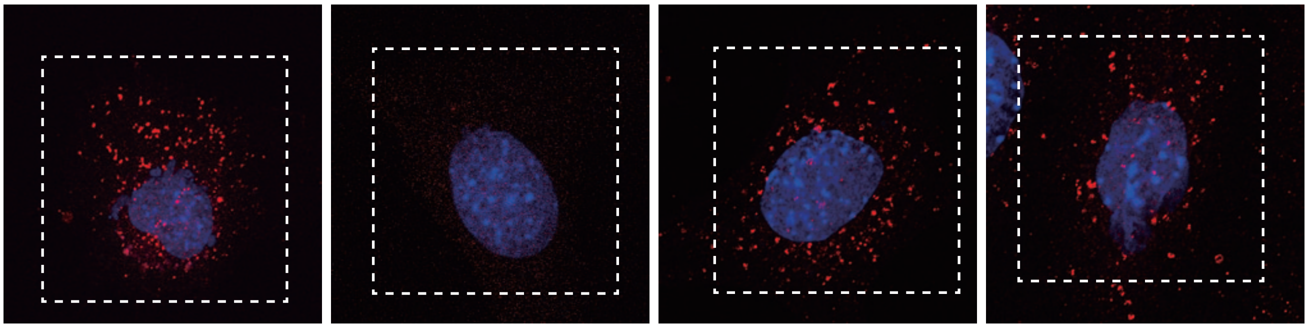

Panel D

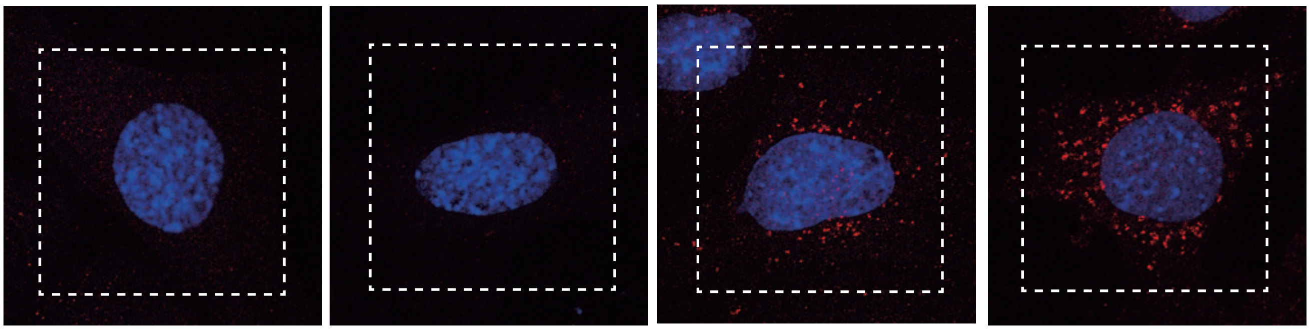

Panel G

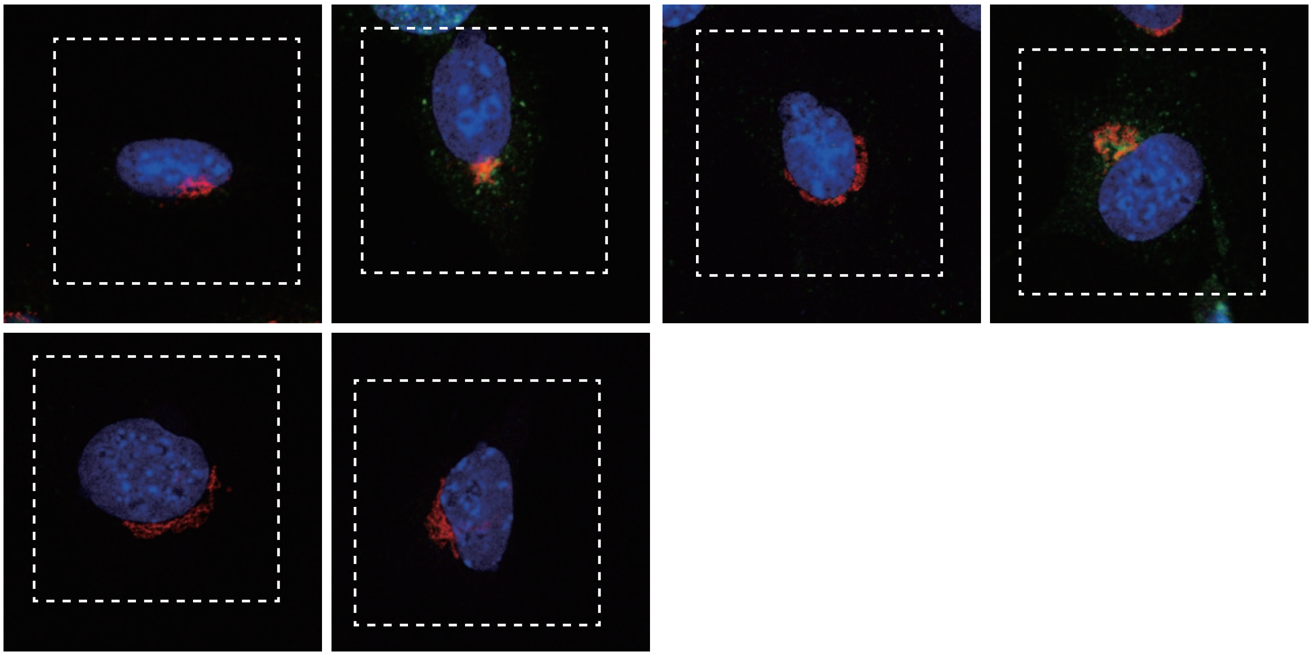

Source data for Fig. 6

Panel A

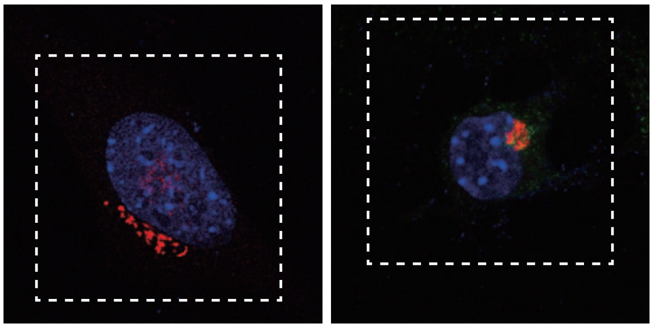

Panel D

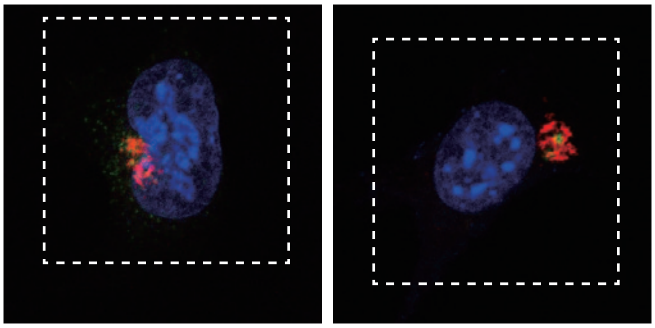

Panel F

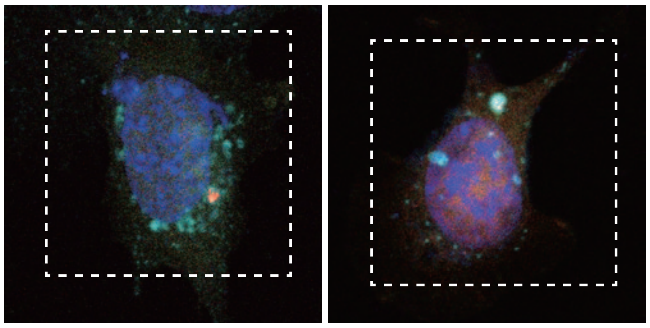

Panel H

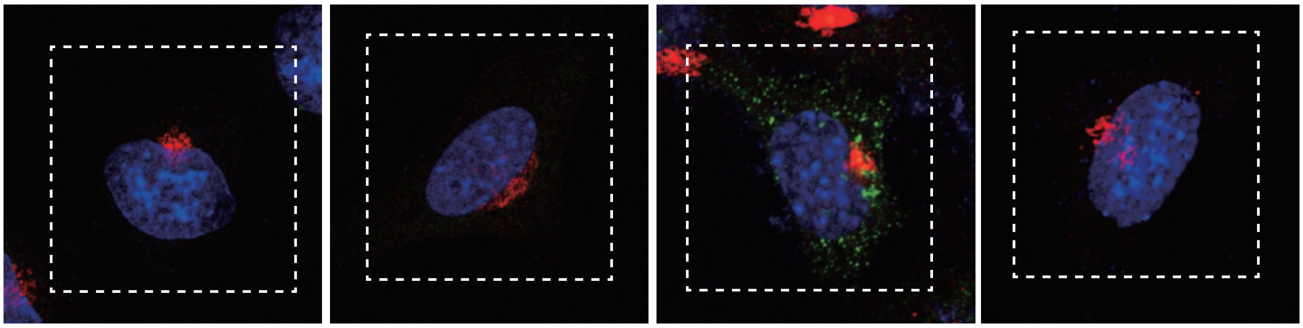

Source data for Fig. 7

Panel B

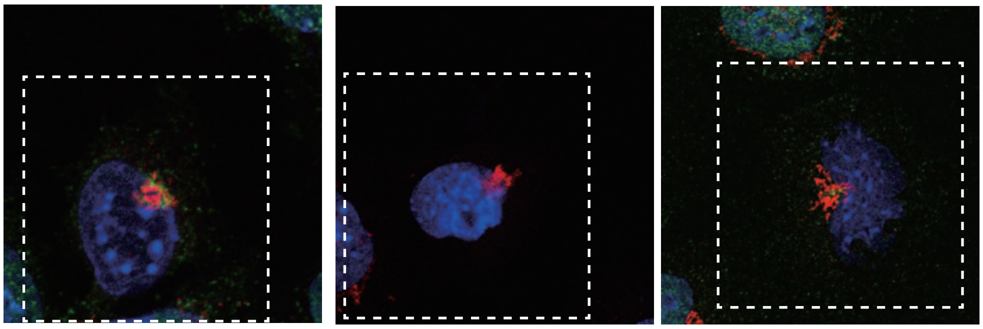

Panel F

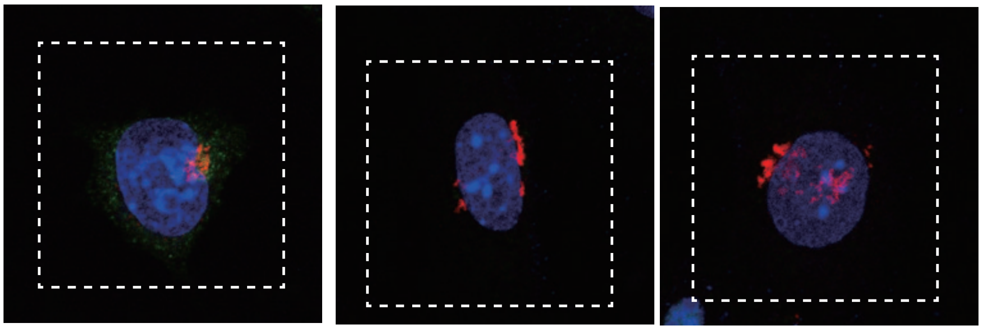

Panel H

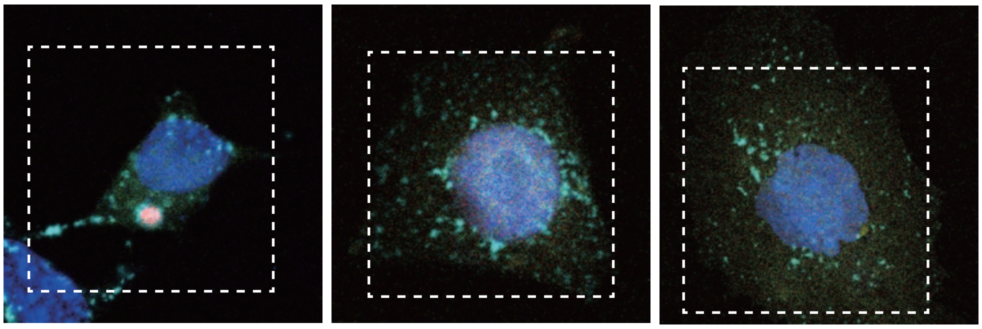

Source data for Fig. 8

Panel C

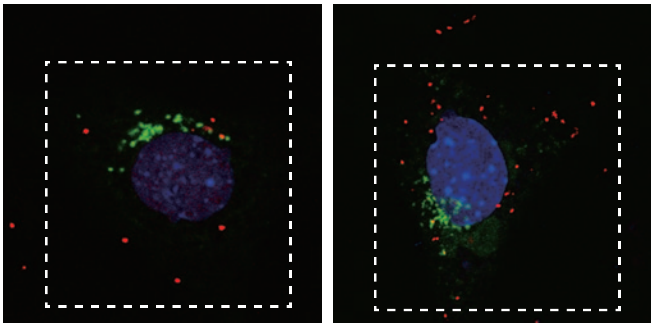

Panel E

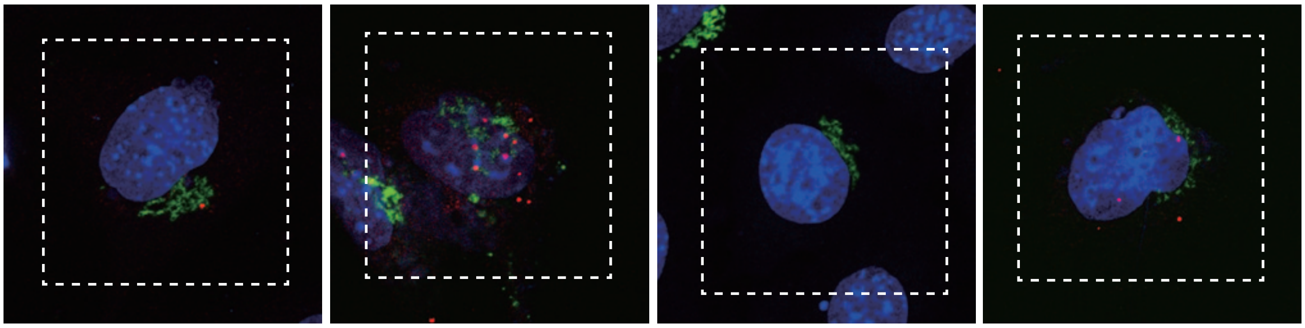

Source data for Fig. 9

Panel B

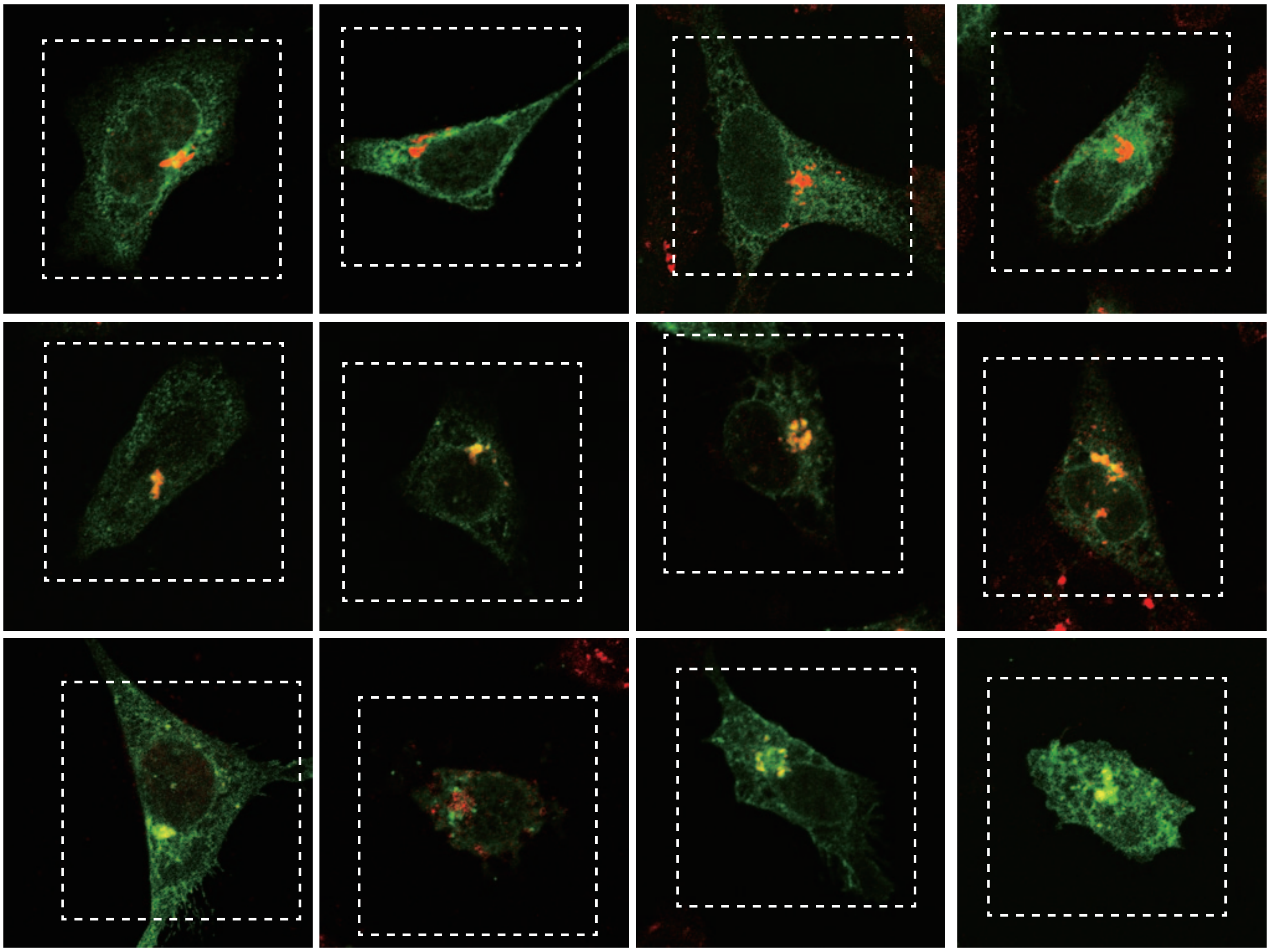

Panel D

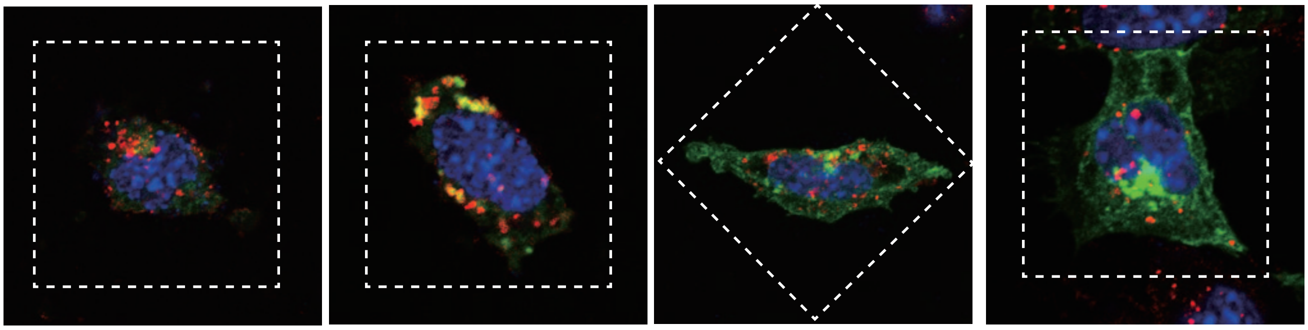

Source data for Fig. 10

Panel A

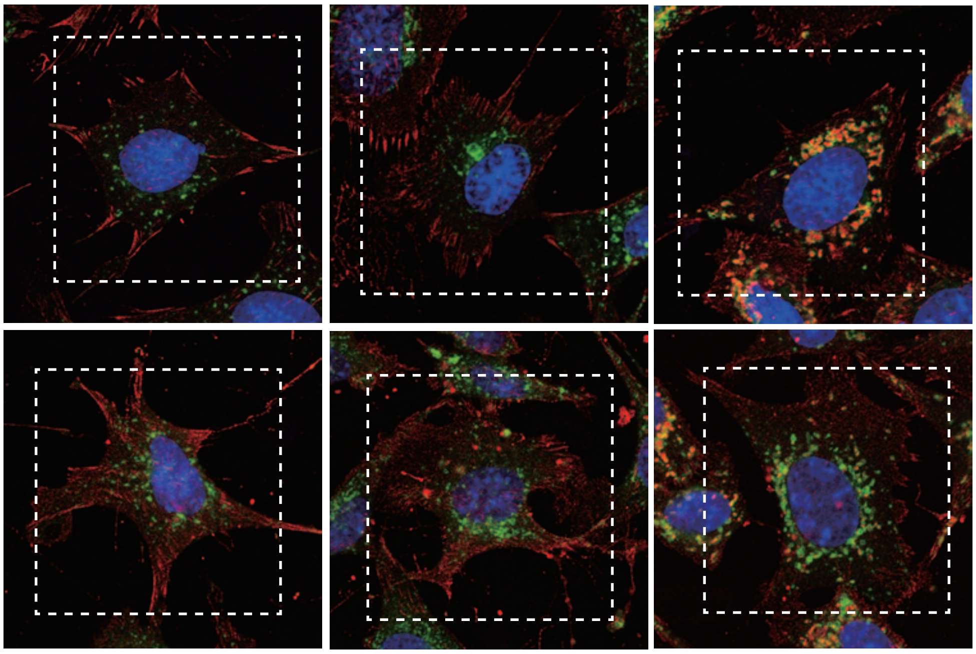

Supplement: Supplementary file 4 — Source Data [file 41467_2020_15577_MOESM4_ESM.pdf]
